# Characterizing the Relation Between Expression QTLs and Complex Traits: Exploring the Role of Tissue Specificity

**DOI:** 10.1007/s10519-018-9914-2

**Published:** 2018-07-20

**Authors:** Hill F. Ip, Rick Jansen, Abdel Abdellaoui, Meike Bartels, UK Brain Expression Consortium, Mina Ryten, John Hardy, Michael E. Weale, Adaikalavan Ramasamy, Paola Forabosco, Mar Matarin, Jana Vandrovcova, Juan A. Botia, Karishma D’Sa, Sebastian Guelfi, Colin Smith, Robert Walker, Regina H. Reynolds, David Zhang, Daniah Trabzuni, Dorret I. Boomsma, Michel G. Nivard

**Affiliations:** 1 Department of Biological Psychology, Vrije Universiteit Amsterdam, Amsterdam, The Netherlands; 2 Department of Psychiatry, VU University Medical Center, Amsterdam, The Netherlands; 3 Amsterdam Public Health Research Institute, Amsterdam, The Netherlands

**Keywords:** Gene expression, Tissue-specificity, Complex human traits, Enrichment, Genome-wide, Brain, Whole blood, Stratified linkage disequilibrium score regression, SLDSR, eQTL discovery

## Abstract

Measurement of gene expression levels and detection of eQTLs (expression quantitative trait loci) are difficult in tissues with limited sample availability, such as the brain. However, eQTL overlap between tissues might be high, which would allow for inference of eQTL functioning in the brain via eQTLs detected in readily accessible tissues, e.g. whole blood. Applying Stratified Linkage Disequilibrium Score Regression (SLDSR), we quantified the enrichment in polygenic signal of blood and brain eQTLs in genome-wide association studies (GWAS) of 11 complex traits. We looked at eQTLs discovered in 44 tissues by the Genotype-Tissue Expression (GTEx) consortium and two other large representative studies, and found no tissue-specific eQTL effects. Next, we integrated the GTEx eQTLs with regions associated with tissue-specific histone modifiers, and interrogated their effect on rheumatoid arthritis and schizophrenia. We observed substantially enriched effects of eQTLs located inside regions bearing modification H3K4me1 on schizophrenia, but not rheumatoid arthritis, and not tissue-specific. Finally, we extracted eQTLs associated with tissue-specific differentially expressed genes and determined their effects on rheumatoid arthritis and schizophrenia, these analysis revealed limited enrichment of eQTLs associated with gene specifically expressed in specific tissues. Our results pointed to strong enrichment of eQTLs in their effect on complex traits, without evidence for tissue-specific effects. Lack of tissue-specificity can be either due to a lack of statistical power or due to the true absence of tissue-specific effects. We conclude that eQTLs are strongly enriched in GWAS signal and that the enrichment is not specific to the eQTL discovery tissue. Until sample sizes for eQTL discovery grow sufficiently large, working with relatively accessible tissues as proxy for eQTL discovery is sensible and restricting lookups for GWAS hits to a specific tissue for which limited samples are available might not be advisable.

**Electronic supplementary material:**

The online version of this article (10.1007/s10519-018-9914-2) contains supplementary material, which is available to authorized users.

## Introduction

The aim of genome-wide association studies (GWAS) is to detect statistically significant associations between single nucleotide polymorphisms (SNPs), and a trait of interest (Hirschhorn and Daly 2005). GWAS have provided insights into the genetic architecture of complex traits (Visscher et al. 2017). However, as a large number of variants identified through GWAS are located outside of coding regions and specific knowledge of regulatory elements is limited, uncovering a relationship between GWAS hits and biological function has proven to be complicated (Lowe and Reddy 2015). Expression quantitative trait loci (eQTLs) are SNPs that influence gene expression, and may aid functional annotation of SNPs that have been identified in a GWAS (Morley et al. 2004; Lowe and Reddy 2015). Previous work has found substantial enrichment of eQTLs among GWAS hits (Nicolae et al. 2010; Torres et al. 2014) and an enrichment in their genome-wide effect on complex traits (Davis et al. 2013). Therefore, eQTLs are viewed as an important tool in moving from genome-wide association to biological interpretation.

As a result of differences in gene expression between cells originating from different tissues, eQTLs are potentially tissue-specific (GTEx Consortium 2015). Tissue-specificity poses no problem if the tissue of interest is readily available for research, such as whole blood. However, discovery of eQTLs gets complicated when measurement of expression levels in a tissue is limited by ethical and practical considerations, for example in brain tissue. Several studies have shown that the overlap between eQTLs from different tissues might actually be larger than initially assumed (Ding et al. 2010; Nica et al. 2011). The Genotype-Tissue Expression (GTEx) consortium identified eQTLs in a wide range of human tissues and showed that 54–90% of the eQTLs identified in one tissue are also designated as an eQTL in at least one other tissue (GTEx Consortium 2015, 2017) and a high average pairwise genetic correlation (r_g_ = 0.738) of local gene expression between tissues was reported by Liu et al. (2017). Therefore, the discovery of eQTLs for tissues such as the brain might be advanced by eQTLs discovered in tissues that are more accessible, such as whole blood. The use of accessible tissues, though, depends on a substantial degree of similarity of eQTL effect across tissue, and the extent to which eQTL differences between tissues are important in complex trait etiology.

An eQTL is commonly viewed as shared between tissues when the same SNP influences a gene in multiple tissues (GTEx Consortium 2015). Alternatively, two eQTLs can be viewed as shared if they influence expression of the same gene in multiple tissues, even though the SNP itself differs between tissues. In this paper, we used a broad and a narrow definition of “tissue-shared eQTL”. In the broad definition, an eQTL was considered shared between two tissues if the SNP tags a gene for which eQTLs were also found in the other tissue (the gene has eQTLs in either tissue). Conversely, an eQTL was tissue-specific if the gene it tagged only had eQTLs in that specific tissue. For the narrow definition of tissue-specific eQTLs, we considered the correlation between the SNP effects on the expression of a gene in one tissue and the SNP effects on expression of the same gene in the second tissue. Where the broad definition of tissue-specificity is based on whether a gene has eQTLs in either tissue at all; the narrow definition is more restrictive, requiring the genetic effects on the expression of a gene to have a positive correlation across tissues (i.e. the same underlying genetic effect on gene expression to be present in two tissues).

To further examine potential tissue-specific eQTL effect on complex traits, we leveraged additional information on the genomic location of eQTLs. Specifically, we extracted eQTLs in regions of the genome where histones have been modified within a specific tissue (i.e. tissue-specific epigenetically changed chromatin states in regulatory regions) (Finucane et al. 2015). We then contrasted the enrichment in GWAS signal for this subset of eQTLs against the enrichment in GWAS signal for all SNPs associated with the tissue-specific epigenetic modification. Finally, we obtained eQTLs associated with the top 10% most strongly differentially expressed genes in each tissue (Finucane et al. 2018) and tested whether these are enriched in their effects on specific complex traits.

For our analyses we leverage large eQTL resources: *cis*-eQTLs per gene discovered in large samples of RNA expression levels assessed in whole blood (N = 4896) (Wright et al. 2014; Jansen et al. 2017) and in brain tissues (N = 134) (Ramasamy et al. 2014). Based on these resources we attempt to detect tissue specific signal in eQTL effects on 11 complex traits. Secondly, we retrieved all eQTLs identified in any of the 44 tissues from the GTEx consortium (N = 70–361, median = 126.5) (GTEx Consortium 2015, 2017). Enrichment is quantified using Stratified Linkage Disequilibrium Score Regression (SLDSR) (Bulik-Sullivan et al. 2015; Finucane et al. 2015).

Our analyses were designed to elucidate the nature of the relation between *cis-*eQTLs and complex traits. We quantified the extent to which this relation is dependent on the tissue used in eQTL discovery. We then considered whether tissue specific information, either epigenetics or the level of gene expression, could help resolve possible tissue-specific eQTL effects on complex traits.

## Materials and methods

### SLDSR method & eQTL annotation definition

A measure of linkage disequilibrium (LD) for each SNP, called an “LD score”, can be computed by taking the sum of correlations between that SNP and all neighboring SNPs (Bulik-Sullivan et al. 2015; Finucane et al. 2015). Under a polygenic model, LD scores are expected to show a linear relationship with GWAS test statistics of corresponding SNPs, where the slope is proportional to h^2^_SNP_. For SLDSR, LD scores are based on only (functional) parts of the genome, called annotations, and used as predictors in a multiple linear regression (Finucane et al. 2015). In this manner, SLDSR is able to partition h^2^_SNP_ into parts that are explained by these annotations (i.e. h^2^_annot_), while accounting for influences of the remaining annotations in the model. The enrichment of an annotation is then obtained by taking the ratio of h^2^_annot_ over the proportion of SNPs that fall within that annotation.

For eQTLs, the number of SNPs to include in the annotation is a complicated quantity: not all significant eQTLs are likely causal; whereas including only lead, or putative causal, eQTLs may result in very narrow annotations located near genes and other regulatory elements, which presents a risk of inflated estimates of the enrichment in GWAS signal. Therefore, we tested the effect of various criteria for inclusion of a SNP into the eQTL annotation. Since eQTLs are essentially discovered in what amounts to a local GWAS, we expected the average LD score of eQTLs to be higher than that of an average SNP, which may influence the results of downstream SLDSR analyses. In order to break the relation between LD score and probability of inclusion, we considered eQTL annotations that were based on a subset of all significant eQTLs for a given probe. First, we included the most strongly associated SNP, a SNP with a high expected LD score, of each probe. Second, we included one SNP per probe with a median *p*-value from the set of significant eQTLs. Third, we included one SNP per probe with a mean *p*-value from the set of significant eQTLs. Fourth, we included the ten most strongly associated SNPs per probe. Finally, we included all SNPs significantly associated with gene expression after FDR correction at α = 0.05. We added each annotation separately to the baseline categories in an SLDSR model, and determined how the various *p* value thresholds influenced the SLDSR coefficient of the eQTL annotation and its corresponding test statistic. For each annotation, we looked up the SNPs in the baseline category, and extracted their baseline LD scores and minor allele frequencies (MAF). We then compared the mean LD score, median LD score and mean MAF between the various eQTL annotations and the entire baseline category. Based on the results (Table S1, Figs. S1 and S2), we considered all significant *cis*-eQTLs as an annotation, and retained additional gene-centric and regulatory annotations in the model.

### Target traits

As outcome for SLDSR, we used summary statistics of GWAS on Crohn’s disease (Jostins et al. 2012), rheumatoid arthritis (Okada et al. 2014), ulcerative colitis (Jostins et al. 2012), BMI (Speliotes et al. 2010), educational attainment (Okbay et al. 2016), schizophrenia (Pardiñas et al. 2018), age at menarche (Perry et al. 2014), coronary artery disease (Schunkert et al. 2011), height (Wood et al. 2014), LDL levels (Teslovich et al. 2010), and smoking behavior (The Tobacco and Genetics Consortium 2010). The first three traits were chosen because they had been related to the immune system and were therefore expected to reveal considerable enrichment of blood eQTL signal (Jostins et al. 2012; Okada et al. 2014). Similarly, brain eQTLs were expected to show substantial enriched effects due to previous reports on the involvement of the central nervous system (CNS) in schizophrenia (Pardiñas et al. 2018), educational attainment (Okbay et al. 2016), and BMI (Vimaleswaran et al. 2012). Of course, these traits did not perfectly align with either tissue, e.g. the immune system has been implicated in the etiology of schizophrenia (Andreassen et al. 2015) and BMI (Karalis et al. 2009). Enrichment of blood and brain eQTL effects on the remaining traits was calculated to contrast the results with traits for which we do not have a strong a priori expectation of the relationship between trait and tissue.

The discovery sample for detection of blood eQTLs (Wright et al. 2014; Jansen et al. 2017) included participants from the Netherlands Twin Register (NTR) (Boomsma et al. 2008) and participants from the Netherlands Study of Depression and Anxiety (NESDA) (Penninx et al. 2008). These two cohorts did not participate in the GWAS for schizophrenia, Crohn’s disease, rheumatoid arthritis, ulcerative colitis, or coronary artery disease. However, participants from these two cohorts, not necessarily the same ones, did participate in the GWAS for height, BMI, LDL levels, smoking behavior, educational attainment, and age at menarche. For educational attainment and smoking behavior, we were able to obtain summary statistics omitting subjects from NTR/NESDA. For both these traits, we looked at trait-specific enrichment of blood and brain eQTL effect in GWAS signal, comparing results from using publicly available datasets with using summary statistics based on the same sample without subjects from the NTR or NESDA. The results did not reveal appreciable differences between the respective datasets for educational attainment, but did show substantial differences for smoking behavior (Fig. S3). This latter finding could conceivably be a function of relatively strong effects of smoking behavior on gene-expression levels (Vink et al. 2015). Therefore, the remaining analyses for smoking behavior were performed using the summary statistics omitting subjects from the NTR and NESDA, whereas analyses for the remaining traits (height, BMI, LDL levels, and educational attainment) were run using publicly available summary statistics. This caveat only applies to eQTL annotations based on NTR/NESDA data (i.e. whole blood). We note that the issue of overlap also applies to other techniques where the error covariance is assumed to be zero [e.g. MetaXcan (Barbeira et al. 2017), Transcriptome-Wide Association Study (TWAS; Gusev et al. 2016), Generalised Summary-data-based Mendelian Randomisation (GSMR; Zhu et al. 2018), etc.]

### Blood and brain eQTL enrichment

Gene expression was quantified by extracting and measuring RNA levels using an array, consisting of several hundreds of thousands of probes (Wright et al. 2014; Ramasamy et al. 2014). Several of these probes (a probe set) were designed to bind to the same RNA sequence, or transcripts, where each transcript represents (a specific form of) a gene. eQTLs were then discovered by running an association analysis between SNP and transcript-level.

A catalog of whole blood *cis*-eQTLs was obtained from Jansen et al. (2017; Wright et al. 2014), where all eQTLs significantly associated with gene expression after FDR correction at α = 0.05 in up to 4896 subjects were included in our whole blood eQTL annotation. A list of brain eQTLs was obtained from the UK brain expression consortium (UKBEC), for which the analyses are described in Ramasamy et al. (2014) and based on brain samples taken from 12 brain regions for 134 Caucasian individuals. We based the brain eQTL annotation on SNPs that were significantly associated with the average gene expression across all 12 brain regions. SLDSR annotations were constructed as per the instructions in Finucane et al. (2015). To guard against upward bias in the eQTL enrichment signal, two extra annotations containing SNPs within a 500 bp (bp) and 100 bp window around any eQTL were constructed for each eQTL set (Finucane et al. 2015). To ensure that the enrichment of eQTL effects in GWAS signal was not in fact caused by their proximity to the genes they influence, an additional gene centric annotation was computed, which contained all SNPs within 1Mbp of all genes for which eQTLs were included. Finally, we performed an inverse-variance weighted meta-analysis across the traits to determine the average effect of blood and brain eQTLs on complex traits in general.

### Tissue-specific eQTL enrichment

To distinguish between the shared and unique effects of eQTLs discovered in whole blood and brain, we used a broad and narrow definition of “tissue-shared eQTL”. For the broad definition of tissue-sharedness, we made a distinction between (a) genes that were only tagged by eQTLs discovered in either tissue and (b) genes for which eQTLs were found in both tissues. Then, the eQTLs were split based on the combination of discovery tissue and genes they tagged. Specifically, the eQTLs were divided into: (1) eQTLs that have been discovered in whole blood and were associated with genes for which only eQTLs were found in whole blood (tissue-specific blood eQTLs), (2) eQTLs that have been discovered in whole blood and were associated with genes for which eQTLs were also found in brain (tissue-shared blood eQTLs), (3) eQTLs that have been discovered in brain tissue and were associated with genes for which only eQTLs have been discovered in brain (tissue-specific brain eQTLs), and (4) eQTLs that have been discovered in brain and were associated with genes for which eQTLs were also found in whole blood (tissue-shared brain eQTLs). Note that, under this definition, the same SNP tagging different genes in different tissues are categorized as tissue-specific.

For the narrow definition of tissue-sharedness, we required a positive correlation in SNP effects on the expression of a gene across tissue. Specifically, we divided all probe sets by the genes they tagged. Then, for each gene, we listed all eQTLs within each probe set and calculated the pairwise correlation in SNP effects on gene expression between all probe sets. Correlations that were based on less than ten overlapping eQTLs were set to missing. Frequently multiple probe sets measure the expression of a single gene, in those cases we computed the average and median correlations between the SNP effects on probe sets which measure gene expression in blood, brain, and across blood and brain probe sets. Finally, we examined the distribution of correlations across genes under various cutoff values for the minimum number of overlapping eQTLs. Based on the various cutoff values we tested, we chose a cutoff of at least 35 overlapping SNPs and a correlation above 0.35. eQTLs were categorized as shared between tissues if they affected a probe set that showed a correlation above the cutoff with at least one other probe set in the other tissue.

### Enrichment of eQTLs obtained in 44 tissues (GTEx)

There are several limitations to above-mentioned analyses of tissue-specific enrichments of eQTL effects in GWAS signal. The eQTLs were obtained from two different projects, which varied in terms of sample size, gene expression array used and their definition of an eQTL. To mitigate the heterogeneity between studies, and to extend to additional tissues, we performed additional analyses using eQTLs obtained by a common pipeline from 44 tissues (see Table S2) (GTEx Consortium 2015, 2017). For each of the 44 tissues, we created annotations for analysis in SLDSR following the previously described procedure. Analogous to the procedure of Finucane et al. (2015) for cell-type-specific analysis using SLDSR, we did not specify windows for the single-tissue GTEx annotations, but included an additional annotation that contained the union of all GTEx eQTLs, i.e. all SNPs that are designated as part of at least one of the 44 single-tissue GTEx annotations, and added a 100 and 500 bp window around this union of GTEx eQTLs. Based on the Z-score of the SNP-heritability (Finucane et al. 2015) and previous reports of substantial influence of either tissue in the etiology of the traits (Okada et al. 2014; Finucane et al. 2015, 2018; Pardiñas et al. 2018), we considered two well-powered traits, one for which we assumed there to be significant enrichment in signal for blood eQTLs (rheumatoid arthritis) and one for brain eQTLs (schizophrenia). For each of these two traits, we ran one SLDSR model containing only the baseline categories and the union of GTEx eQTLs. Furthermore, 44 additional models were fitted to both traits, each model containing the baseline categories, the union of GTEx eQTLs and one of the 44 single-tissue GTEx annotations.

GTEx has relative small sample sizes for the discovery of brain eQTLs (mean = 89, range = 72–103) compared to discovery of eQTLs in other tissues (mean = 160, range = 70–361) (GTEx Consortium 2015, 2017). To investigate the effect of differences in sample size on estimates of enrichments in GWAS signal, we collapsed the union of individual brain eQTL annotations into a shared brain eQTL annotation (i.e. an eQTL found in at least one of the GTEx brain annotations was included in the shared brain eQTL annotation). This annotation was then analyzed as an additional GTEx eQTL annotation in schizophrenia. We further tested the relationship between tissue sample size and tissue eQTL enrichment.

### Enrichment of the intersection between eQTLs and histone marks

Finucane et al. (2015) identified SNPs that were associated with tissue-specific histone marks, a type of epigenetic modification related to enhancers and promoters of actively transcribed genes. Out of the 220 cell-type-specific histone marks that were available, 100 were found in the CNS or in immune tissues (Table S3). For each of the 100 annotations, we extracted its intersection with the union of GTEx eQTLs (i.e. SNPs found in both annotations) and made a new SLDSR annotation. We then applied 100 SLDSR models to summary statistics of schizophrenia and rheumatoid arthritis where each model contained the baseline categories, the union of GTEx eQTLs, one of the 100 cell-type-specific histone marks and its corresponding intersection annotation. Enrichments in GWAS signal of the intersection should be interpreted as enrichment of genome-wide SNP effects on a complex trait beyond the additive effects that work on all SNPs that are a *cis*-eQTL and histone mark in question. In fact, we tested whether the interaction between tissue-specific chromatin state and eQTLs were enriched in their genome-wide effect on complex traits.

### GTEx eQTLs for tissue-specific differentially expressed genes

Finucane et al. (2018) looked at tissue-specific gene expression and determined that the top 10% of these differentially expressed genes are substantially enriched in their effects in GWAS signals for a wide range of traits. Here, we built on these findings by taking the top 10% most strongly differentially expressed genes in the 44 GTEx tissues and extracting the eQTLs for these specific genes, regardless of the discovery tissue. These were separately added as an annotation to an SLDSR model together with the baseline categories and union of GTEx eQTLs. A significant increase in enrichment in GWAS signal in the eQTLs compared to the genes themselves, would indicate that eQTLs explain part of the enrichment seen by Finucane et al.

## Results

### SLDSR eQTL annotation definition

We compared five annotations that included various SNPs based on the *p* value of their associations with gene-expression levels (lead eQTL, median eQTL, mean eQTL, top 10 lead eQTLs, and all eQTLs). Supplementary Table S1 shows various metrics of these annotations. Surprisingly, lead eQTLs had the lowest mean and median LD score amongst the annotations, indicating that the annotation contained less signal (Table S1). However, it was still higher compared to the mean or median LD score of all SNPs in the baseline annotation. Including all significant eQTLs in the annotation resulted in the highest mean and median LD score. All annotations had a mean MAF 0.27–0.28, whereas the mean MAF of the entire baseline category was 0.24. Figure S1 plots the enrichment in GWAS signal for blood eQTLs for one annotation against the other annotations. Smaller annotations had a higher enrichment in GWAS signal; however, the enrichment in GWAS signal did not differ between taking the lead eQTL, eQTLs with a mean *p* value, or eQTLs with a median *p*-value. Figure S2 plots the coefficient Z-score of the various annotations against one another. Coefficient Z-score did not differ much between the annotations. Since including all significant eQTLs did not result in a decrease of the mean or median LD score compared to the other annotations tested here and did result in larger annotations, we selected the annotation based on all significant eQTLs for further analyses.

### Blood and brain eQTL enrichment

We fitted an SLDSR model containing the baseline categories, the complete annotation for both brain and blood eQTL tissues, their 100 and 500 bp windows, and gene-centric annotations to all traits (Crohn’s disease, rheumatoid arthritis, ulcerative colitis, BMI, educational attainment, schizophrenia, age at menarche, coronary artery disease, height, LDL levels, and smoking behavior). We performed one-tailed tests for enrichment for each annotation and corrected for multiple testing across annotations within trait. We found significant effects of brain eQTLs on educational attainment, rheumatoid arthritis, smoking behavior, and schizophrenia (Table S4A–K). Blood eQTLs showed significantly enriched effects on height and smoking behavior. The gene-centric annotation for both blood and brain eQTLs showed no effect on any trait after correction for multiple testing. We then meta-analyzed the results for all annotations, both in the baseline model, and those associated with eQTLs across the 11 traits. Our analysis revealed significant effect of both blood (*p* < 0.001) and brain (*p* < 0.001) eQTL effects (Table S5), exceeding, in terms of significance, all the baseline categories considered by Finucane et al. (2015) except for conserved genomic regions.

### Tissue-specific eQTL enrichment

We used a broad definition of tissue-sharedness in eQTL effects to separate the list of blood eQTLs into a list of tissue-specific blood eQTLs and a list of blood eQTLs with shared effects across tissue. We then modelled the baseline categories together with all blood eQTLs and the tissue-specific blood eQTLs. The same was done for brain eQTLs. We observed no evidence for enrichment of blood-specific eQTLs (relative to all blood eQTLs) on immune-related traits, nor do we find significant enrichment of effect on brain-related traits of eQTLs associated with genes for which eQTLs were solely identified in brain tissue (Tables 1, 2).

**Table 1:** Coefficients of complete blood eQTL annotation and broad tissue-specific blood eQTLs

Trait	Complete blood eQTLs	Unique blood eQTLs (broad)
Crohn’s disease	6.115e−07(3.987e−07)	− 1.459e−07(4.946e−07)
Rheumatoid arthritis	2.352e−07(8.077e−08)***	− 1.291e−07(1.397e−07)
Ulcerative colitis	7.904e−08(2.073e−07)	2.003e−07(2.736e−07)
BMI	1.484e−07(8.701e−08)*	− 5.728e−08(7.924e−08)
Educational attainment	4.386e−08(1.936e−08)*	− 1.806e−08(2.483e−08)
Schizophrenia	1.604e−08(2.167e−07)	5.361e−07(3.039e−07)*
Age at menarche	6.830e−08(6.127e−08)	− 3.606e−08(7.277e−08)
Coronary artery disease	6.874e−09(6.288e−08)	2.397e−08(7.847e−08)
Height	2.863e−07(8.099e−08)***	− 8.241e−08(1.318e−07)
LDL levels	2.161e−07(1.042e−07)*	− 1.908e−07(1.387e−07)
Smoking behavior	1.202e−07(4.854e−08)**	− 1.034e−07(6.523e−08)

Values between brackets indicate standard errors. One asterisk indicates categories reaching nomial significance (*p* < 0.05). Two asterisks indicate categories passing FDR correction for multiple testing (α = 0.05). Three asterisks indicate categories passing Bonferroni correction for multiple testing

**Table 2:** Coefficients of complete brain eQTL annotation and broad tissue-specific brain eQTLs

Trait	Complete brain eQTLs	Unique brain eQTLs (broad)
Crohn’s disease	1.076e−06(4.447e−07)**	− 2.724e−07(5.644e−07)
Rheumatoid arthritis	3.099e−07(1.234e−07)**	− 7.296e−08(1.483e−07)
Ulcerative colitis	1.482e−07(2.250e−07)	6.010e−08(3.236e−07)
BMI	9.540e−08(4.289e−08)**	− 6.726e−08(7.140e−08)
Educational attainment	7.149e−08(2.037e−08)***	− 4.628e−08(3.245e−08)
Schizophrenia	7.849e−07(2.655e−07)***	− 6.929e−08(3.930e−07)
Age at menarche	7.583e−08(4.896e−08)	− 3.136e−08(7.101e−08)
Coronary artery disease	2.820e−08(5.694e−08)	− 5.672e−08(8.192e−08)
Height	2.505e−07(8.777e−08)***	− 7.998e−08(1.261e−07)
LDL levels	1.707e−07(8.394e−08)*	− 3.242e−07(1.019e−07)
Smoking behavior	8.208e−08(6.311e−08)	− 3.084e−08(8.465e−08)

Values between brackets indicate standard errors. One asterisk indicates categories reaching nomial significance (*p* < 0.05). Two asterisks indicate categories passing FDR correction for multiple testing (α = 0.05). Three asterisks indicate categories passing Bonferroni correction for multiple testing

Next, we used a narrow definition of tissue-sharedness to again make the distinction between tissue-specific blood eQTLs and blood eQTLs that show a cross-tissue effect. We then modelled the baseline categories together with all blood eQTLs and the unique blood eQTLs. The same was done for the brain eQTLs. Figures S4 and S5 show the distribution of mean correlations across genes, within respectively blood and brain probe sets. Most probe sets showed a moderate to high, positive correlation, with a long tail to the left. The mean correlation across genes within respectively blood and brain probe sets was 0.63 and 0.67. The mean number of eQTLs that overlapped between probe sets, within tissue was 214 (blood) and 158 (brain). Across tissue, the mass of the distribution of correlations was more spread across the range, although a sharp increase was seen at roughly 0.35 (Fig. S6). Compared to the analyses within tissues, the mean correlation between eQTL effects in expression in brain and blood was 0.25. The average number of number of overlapping eQTLs between brain and blood probes for the same gene was 139. Similar to the analyses using the broad definition of tissue-sharedness, blood-specific eQTLs were not enriched in GWAS signal for immune related traits (Table 3). Likewise, brain-specific eQTLs showed no significant enrichment in their effect on brain-related traits (Table 4).

**Table 3:** Coefficients of complete blood eQTL annotation and narrow tissue-specific blood eQTLs

Trait	Complete blood eQTLs	Unique blood eQTLs (narrow)
Crohn’s disease	9.431e−07(5.744e−07)	− 4.406e−07(6.256e−07)
Rheumatoid arthritis	2.356e−07(1.519e−07)	− 8.019e−08(1.679e−07)
Ulcerative colitis	2.507e−07(4.785e−07)	− 6.476e−08(4.908e−07)
BMI	1.518e−07(8.285e−08)*	− 3.952e−08(8.619e−08)
Educational attainment	4.712e−08(3.128e−08)	− 1.472e−08(3.432e−08)
Schizophrenia	5.029e−07(4.088e−07)	− 1.851e−07(4.406e−07)
Age at menarche	− 2.363e−09(1.289e−07)	5.540e−08(1.281e−07)
Coronary artery disease	− 2.998e−08(1.075e−07)	5.611e−08(1.199e−07)
Height	8.629e−08(1.980e−07)	1.674e−07(2.148e−07)
LDL levels	− 3.215e−08(1.260e−07)	1.557e−07(1.412e−07)
Smoking behavior	1.819e−07(8.704e−08)*	− 1.316e−07(9.085e−08)

Values between brackets indicate standard errors. One asterisk indicates categories reaching nomial significance (*p* < 0.05)

**Table 4:** Coefficients of complete brain eQTL annotation and narrow tissue-specific brain eQTLs

Trait	Complete brain eQTLs	Unique brain eQTLs (narrow)
Crohn’s disease	9.500e−07(1.040e−06)	2.371e−08(1.036e−06)
Rheumatoid arthritis	6.997e−07(3.364e−07)*	− 5.031e−07(3.290e−07)
Ulcerative colitis	− 2.301e−08(3.799e−07)	2.441e−07(4.207e−07)
BMI	7.360e−08(8.037e−08)	− 2.821e−09(8.464e−08)
Educational attainment	7.571e−08(4.264e−08)*	− 3.154e−08(4.577e−08)
Schizophrenia	3.203e−07(3.878e−07)	5.190e−07(4.362e−07)
Age at menarche	1.130e−07(1.170e−07)	− 6.058e−08(1.239e−07)
Coronary artery disease	1.076e−08(9.983e−08)	− 1.084e−08(1.121e−07)
Height	1.300e−07(1.240e−07)	9.491e−08(1.496e−07)
LDL levels	1.216e−07(1.794e−07)	− 1.096e−07(2.002e−07)
Smoking behavior	1.231e−07(7.249e−08)*	− 7.162e−08(8.370e−08)

Values between brackets indicate standard errors. One asterisk indicates categories reaching nomial significance (*p* < 0.05)

### Enrichment of eQTLs obtained in 44 tissues (GTEx)

**Fig. 1:**
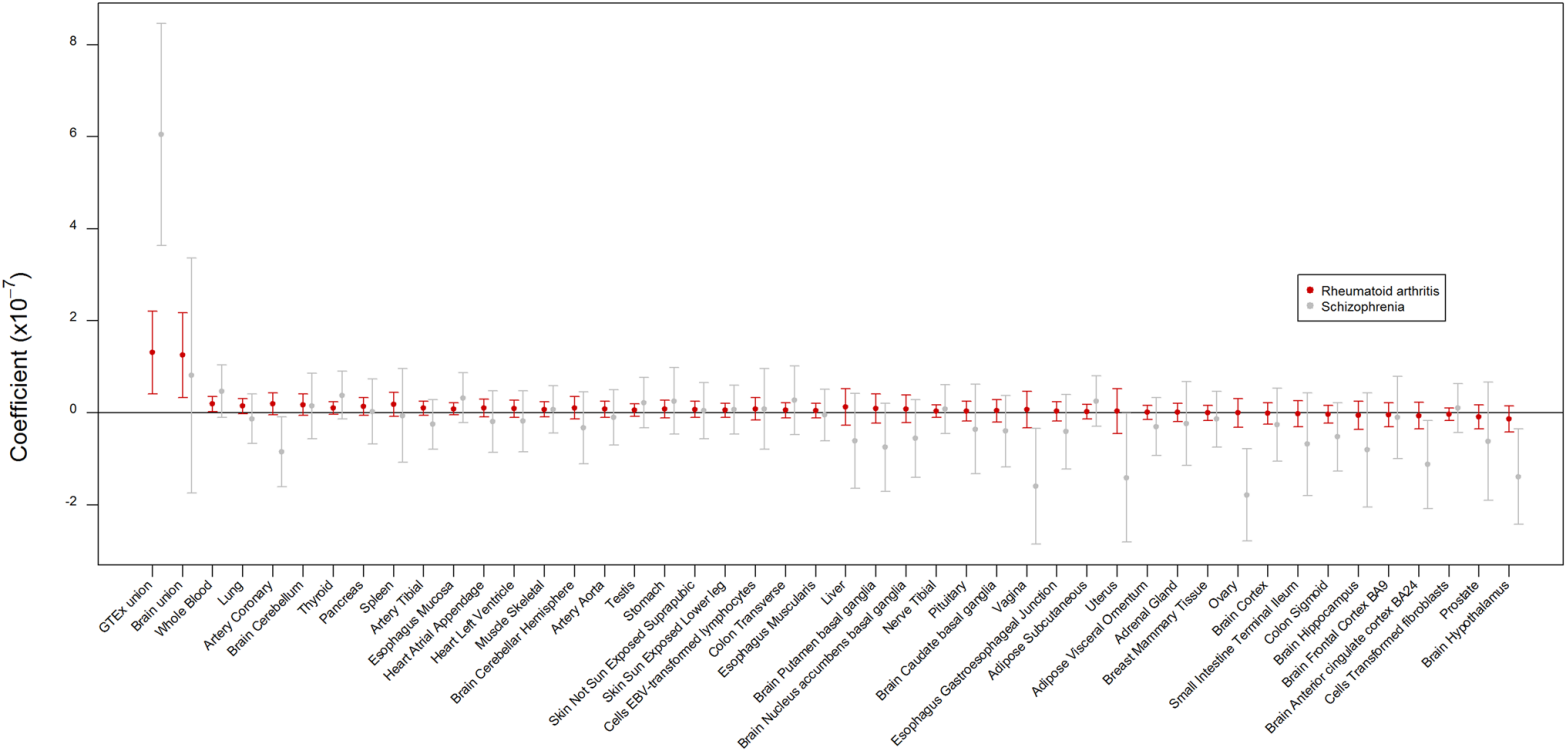
Coefficient Z-scores of the GTEx annotations. Barplot of coefficient z-scores for all GTEx annotations for schizophrenia (grey) and rheumatoid arthritis (red). Bars are sorted from highest to lowest based on the results from rheumatoid arthritis. (Color figure online)

We interrogated the enrichment of the union of GTEx eQTLs and 44 single-tissue GTEx annotations in their effect on schizophrenia and rheumatoid arthritis. Figure 1 shows the coefficient of these GTEx annotations, sorted on their Z-scores for rheumatoid arthritis. In both cases, the union of GTEx eQTLs contributed significantly to explaining the polygenic signal (Table S6), indicating that eQTLs were significantly enriched in their effects on complex traits. The single-tissue annotations, however, performed notably worse in terms of their genome-wide effects on schizophrenia and rheumatoid arthritis. For rheumatoid arthritis, the coefficient Z-scores of the whole blood annotation reached nominal significance (Z = 2.251), but failed correction for multiple testing. None of the other annotations reached nominal significance. The union of all GTEx brain annotations did not contribute significantly to explaining h^2^_SNP_ of schizophrenia (Z = 0.621, *p* = 0.267). Sample size in the eQTL discovery phase appeared to be a strong determinant of tissue-specific enrichment in GWAS signal. The correlation coefficients between the coefficient Z-scores and sample sizes were 0.658 (*p* < 0.001) and 0.467 (*p* = 0.001) for schizophrenia and rheumatoid arthritis, respectively (Table S6).

### Enrichment of the intersection between eQTLs and histone marks

We extracted the intersection of eQTLs and histone marks found in specific CNS and immune cells, and estimated the enrichment of the intersection in its effect on rheumatoid arthritis and schizophrenia. We found significant enrichment in GWAS signal for eQTLs that intersected with histones bearing modification H3K4me1, a modification thought to be present in the enhancer of actively transcribed genes (Zhou et al. 2011; Allis and Jenuwein 2016), in CNS cells for schizophrenia (see Table S7). There was some evidence for significant enrichment of eQTLs that intersected with genomic regions in immune cells bearing the H3K4me1 mark in their effect on schizophrenia, but not on rheumatoid arthritis. Specifically, none of the annotations that contained the intersection between eQTL and cell-type-specific histone modification showed evidence of enrichment for rheumatoid arthritis (Table S8). The union of GTEx eQTLs reached statistical significance for all models. For the separate annotations, we found significant enrichment in GWAS signal across most histone marks found in CNS cells and three significant immune cell-types that bore the H3K4me3 modification, a modification associated with transcriptional start sites and promoters of actively transcribed genes (Zhou et al. 2011; Allis and Jenuwein 2016), for schizophrenia (Table S9). The opposite picture was seen for rheumatoid arthritis: a wide variety of immune-cell specific histone marks showed significant enrichments in GWAS signal, while coefficients for most marks found in CNS cells were below zero (Table S10).

### GTEx eQTLs for tissue-specific differentially expressed genes

The enrichment in GWAS signal for the eQTLs for the top 10% most specifically expressed genes in a tissue correlated 0.58 and 0.24 with the enrichment in GWAS signal for the body of the specifically expressed genes reported by Finucane et al. (2018) for schizophrenia and rheumatoid arthritis, respectively. eQTLs for differentially expressed genes in brain tissues were top-ranked compared to other tissues in terms of their coefficients and Z-scores, but were not significantly enriched. None of the coefficients for the eQTL annotations surpassed the significance threshold after correction for multiple testing (Table S11). This indicates that these eQTLs contribute most strongly to the overall SNP-heritability. Furthermore, the eQTL annotations showed larger coefficients compared to corresponding annotations of whole genes (Finucane et al. 2018). For rheumatoid arthritis, eQTLs associated with differentially expressed genes for whole blood showed the most significant coefficient, but again failed correction for multiple testing (Table S11).

## Discussion

Stratified Linkage Disequilibrium Score Regression provides a way to partition h^2^_SNP_ into fractions explained by (functional) parts of the genome. A “full baseline model” containing 24 non-cell-type-specific annotations of SNPs, such as SNPs located in promoters or coding regions, was developed for analyses with SLDSR (Finucane et al. 2015). Here, we added annotations containing eQTLs derived from whole blood and brain tissue into the model, and showed that eQTLs were substantially stronger enriched in their effect on complex traits compared to all baseline categories, except for conserved genomic regions. The complete blood eQTL annotation was significantly enriched in GWAS signal for rheumatoid arthritis. The complete brain eQTL annotation was significantly enriched in GWAS signal for schizophrenia, which is consistent with previous estimates of eQTL effect enrichment (Davis et al. 2013). Considerable enrichment for eQTLs, even for traits not apparently linked to the brain or immune system (e.g. smoking behavior), suggested that non-trivial eQTL overlap across tissues might be present.

Inclusion of both brain and blood eQTLs into the SLDSR model did not separate the signal into tissue-specific effects. In general, we were not able to clearly identify tissue-specific eQTL signals with these datasets and SLDSR. For type-II diabetes (T2D), Torres et al. (2014) considered the effects of eQTLs that were identified in either one of three tissues (whole blood, adipose tissue and skeletal-muscle tissue). Only muscle-specific eQTLs were enriched in their effect on T2D. Conversely, eQTLs that were discovered in all three tissues explained larger part of the phenotypic variance of T2D and were stronger enriched in their effect on T2D. These findings are largely in line with our analyses on the 44 single-tissue GTEx eQTL sets. We found that, while an annotation containing all eQTLs identified in GTEx was significantly enriched in its effect on schizophrenia and rheumatoid arthritis [Z = 4.911 (*p* < 0.001) and Z = 2.871 (*p* = 0.004) respectively], none of the analyzed brain tissues were enriched beyond all eQTLs in their effect on schizophrenia. Similarly, whole blood eQTLs were not significantly enriched beyond all GTEx eQTLs taken together in their effect on rheumatoid arthritis. Again, these findings are not consistent with the hypothesis of abundant tissue-specific *cis*-eQTLs with effects on complex traits related to the specific tissue in question. Our findings further support a lack of power to detect any tissue-specific eQTL effects. This lack of power may be partially driven by the small physical distance between eQTLs, as any *cis*-eQTL is by definition within 1Mbp or even 250Kbp of a gene. This makes it very likely that the eQTLs in one tissue are in strong LD with the true causal eQTL in another tissue, complicating detection of tissue specific effects.

Finucane et al. (2015) examined the enrichment in effect of 220 tissue-specific epigenetically modified regions on various human traits and showed that epigenetic modifications in tissues most relevant to the etiology of those traits were top-ranked among the results. Finucane et al. (2018) looked at differentially expressed genes across tissue and calculated the enrichment in GWAS signal for these genes for multiple human traits. In line with the results for tissue-specific epigenetically modified regions, the results showed strong enrichment of GWAS signal for genes that were differentially expressed in trait-relevant tissues. Here, we took the intersection between tissue-specific epigenetically modified regions and the union of GTEx eQTLs. We find evidence for possible enrichment for eQTLs that intersected with tissue-specific H3K4me1 histone marks in both brain and immune cells in their effect on schizophrenia, but not for rheumatoid arthritis. Thus, eQTLs in H3K4me1 marks were enriched in their effect on schizophrenia above the expected enrichment based on the fact that these SNPs were both eQTLs and located in H3K4me1 histone marks. What is of substantial interest is that the enrichment in GWAS signal appeared specific to H3K4me1 marks, and not to other histone marks, suggesting that these marks specifically can aid in prioritizing genomic regions in which tissue-specific eQTLs may reside. Especially when contrasted with tissue-specific gene expression levels and tissue-specific histone modifications, tissue-specific eQTLs are of limited value in relating complex traits to a tissue. In fact, considering eQTLs associated with genes that are differentially expressed in a specific tissue identifies stronger enrichment in tissue-specific effects. While specifically expressed genes are enriched in their effects on complex traits related to the tissue of interest, eQTLs for these genes are not. The primary utility of eQTL studies for complex traits appear to lie in their ability to link genes with trait, irrespective of tissue, through MetaXcan (Barbeira et al. 2017), TWAS (Gusev et al. 2016), or GSMR (Generalised Summary-data-based Mendelian Randomisation; Zhu et al. 2018).

One of the limitations of our work involves the substantial differences in discovery sample size between the tissues, which influences the power to detect eQTLs (Lonsdale et al. 2013). Even within the GTEx tissues, where differences in sample sizes are relatively small compared to the difference between eQTLs obtained from Jansen et al. (2017) and Ramasamy et al. (2014), we still saw a significant correlation between the discovery sample size and enrichment of eQTLs in GWAS signal. Several methods have been developed to capitalize on cross-tissue overlap in eQTLs to improve power to detect SNP effects on gene expression within tissue. Flutre et al. (2013) and Li et al. (2017) proposed two Bayesian approaches to jointly link gene expression levels measured in multiple tissues to genome-wide SNPs. Their methods put a stronger prior on a SNP being an eQTL within a tissue with increasing evidence of the SNP being an eQTL across several tissues, resulting in an increased power to detect tissue-shared eQTLs. The primary aim of our paper was to explore assessment of the effects of eQTLs expressed in whole blood on presumably brain-related traits, and vice versa. Methods such as TWAS and GSMR rely on eQTLs that have been discovered in tissues that have not been linked to the etiology of the trait of interest. It is therefore of interest to test the tissue specificity of eQTLs discovered in single issues. TWAS and GSMR have not yet been applied to multi-tissue eQTLs and, as such, performing a second discovery of multi-tissue eQTLs in a GTEx content was beyond the scope of our study. Rather, we constructed an annotation containing the union of GTEx eQTLs, which may underestimate the true number of eQTLs but sufficed for addressing the primary aim of our paper. Note that GTEx release version seven includes a multi-tissue analysis and the increased power to detect tissue-shared eQTLs might allow for a more accurate partitioning of the SNP-heritability. We showed, in the analyses with eQTLs within differentially expressed genes, that enrichment in GWAS signal is stronger in these eQTLs compared to taking all SNPs in the same genes. This indicates that eQTLs, irrespective of the tissue in which they have been discovered, play an important role in the etiology of complex traits, and do so via the gene they are associated with. This does not take away the need to increase sample sizes when performing tissue-specific discovery of (*cis-*)eQTLs. Tissue specificity, in the end, is a relative judgement best reached based on weighing multiple lines of evidence, among which are differential expression, epigenetic regulation, and eQTLs. For eQTLs to play a large role in determining the tissue-specific effects on complex traits, a continued investment in resources like GTEx is required in order to increase sample sizes for detection, especially in rare tissues.

Our conclusions currently are limited to *cis*-eQTLs and may not generalize to *trans*-eQTLs which are more tissue specific. Our results are consistent with, and complimentary to, the work of Liu et al. (2017), which examined the genetic correlation between gene expression levels across 15 tissues. This revealed substantial correlations between *cis*-genetic effects on gene expression, but not between *trans* effects, across 15 tissues. Our analyses confirmed the value of using whole blood as discovery tissue for detection of *cis*-eQTLs and further demonstrated the usefulness of techniques that use *cis*-eQTLs discovered in whole blood to study the etiology of complex traits related to different tissues (Gamazon et al. 2015; Gusev et al. 2016). The results presented here highlight the overlap of *cis*-eQTL effects across tissues on a genome-wide level. However, the effect of a *cis*-eQTL might vary substantially across tissues for individual genes (Grundberg et al. 2012). Our conclusions were based on genome-wide enrichments and therefore should not be interpreted as limited evidence for tissue-specific eQTL effects for individual genes. Therefore, eQTL discovery in the tissue most relevant to a specific trait or disorder remains important to further our understanding of the genetic regulation of tissue-specific gene expression. What is also clear is that to discover those tissue-specific eQTLs that are of relevance to the interpretation of GWAS of complex traits, tissue-specific eQTL discovery needs to be refined. The practice of, as a post-hoc analysis to GWAS, performing eQTL lookup in a specific tissue linked to a trait, when larger dataset for other accessible tissues are available, may be suboptimal. In fact, one may prefer to perform a lookup in the overlap between histone modifications in a relevant tissue and eQTLs regardless of tissue. One can further consider utilizing eQTLs to link GWAS findings to a gene, and subsequently consider the differential expression of a gene to identify the tissue in which the gene is most likely to act in effecting the trait. Tissue-specific differential gene expression vastly outperforms eQTLs in tagging regions of the genome enriched in their effect on complex traits.

It is also evident that a limited dichotomous definition of eQTL/no-eQTL may be insufficient to identify tissue-specific eQTL effects. One improvement would be to compute the *difference* in eQTL effect on expression of the gene between tissues, and perform inference based on this difference in effect. eQTLs are strongly enriched SNPs, with clear biological function and utility for the translation of GWAS findings, though tissue-specific eQTL mechanisms remain elusive. The discovery of tissue-specific eQTL effects, which can aid in linking complex trait to tissue, may require novel research strategies.

## Electronic supplementary material

Below is the link to the electronic supplementary material.

Supplementary material (DOCX 159 KB)

Figure S1 (TIFF 12372 KB)

Figure S2 (TIFF 12372 KB)

Figure S3 (TIFF 15056 KB)

Figure S4 (TIFF 5035 KB)

Figure S5 (TIFF 5035 KB)

Figure S6 (TIFF 5035 KB)
