# Supplementary material for: Characterizing the Relation Between Expression QTLs and Complex Traits: Exploring the Role of Tissue Specificity
Source: Behav Genet. 2018 Jul 20;48(5):374–85. doi: 10.1007/s10519-018-9914-2 (PMC6097736; doi:10.1007/s10519-018-9914-2)
Supplement: Supplementary file 1 — Supplementary material (DOCX 159 KB) [file 10519_2018_9914_MOESM1_ESM.docx]

This document describes the supplementary tables and figures to Characterizing the relation between expression QTLs and complex traits: exploring the role of tissue specificity

Hill F. Ip, Rick Jansen, Abdel Abdellaoui, Meike Bartels, UK Brain Expression Consortium, Dorret I. Boomsma, & Michel G. Nivard

Content: 21 tables and 6 figures

**Table S1 . Descriptives of various annotations based on different *p*-value thresholds**

|  | Number of SNPs | Prop. SNPs | Mean LD score | Median LD score | Mean MAF |
| --- | --- | --- | --- | --- | --- |
| base | NA | NA | 122.722 | 88.896 | 0.237 |
| lead eQTL | 6926 | 0.0011 | 130.524 | 99.983 | 0.283 |
| mean log10(p) | 6997 | 0.0011 | 137.727 | 105.352 | 0.281 |
| median log10(p) | 6978 | 0.0011 | 138.160 | 105.145 | 0.276 |
| 10 strongest hits | 60192 | 0.0095 | 144.388 | 113.416 | 0.287 |
| all hits | 1203378 | 0.1826 | 174.584 | 135.262 | 0.273 |

From left to right, columns indicate 1) which subset of eQTLs were analyzed; 2) the number of SNPs included in the annotation; 3) the proportion of SNPs, out of all SNPs in the baseline annotation, included in the annotation; 4) the mean LD score; 5) the median LD score; and 6) the mean minor allele frequency.

**Table S2. List of available GTEx tissues**

| Adipose Subcutaneous | Heart Atrial Appendage |
| --- | --- |
| Adipose Visceral Omentum | Heart Left Ventricle |
| Adrenal Gland | Liver |
| Artery Aorta | Lung |
| Artery Coronary | Muscle Skeletal |
| Artery Tibial | Nerve Tibial |
| Brain Anterior cingulate cortex BA24 | Ovary |
| Brain Caudate basal ganglia | Pancreas |
| Brain Cerebellar Hemisphere | Pituitary |
| Brain Cerebellum | Prostate |
| Brain Cortex | Skin Not Sun Exposed Suprapubic |
| Brain Frontal Cortex BA9 | Skin Sun Exposed Lower leg |
| Brain Hippocampus | Small Intestine Terminal Ileum |
| Brain Hypothalamus | Spleen |
| Brain Nucleus accumbens basal ganglia | Stomach |
| Brain Putamen basal ganglia | Testis |
| Breast Mammary Tissue | Thyroid |
| Cells EBV-transformed lymphocytes | Uterus |
| Cells Transformed fibroblasts | Vagina |
| Colon Sigmoid | Whole Blood |
| Colon Transverse |  |
| Esophagus Gastroesophageal Junction |  |
| Esophagus Mucosa |  |
| Esophagus Muscularis |  |

**Table S3. List of available cell-type-specific histone modification in cells located in either the central nervous system or immune system**

| Tissue | Cell-type | Histone modification |
| --- | --- | --- |
| CNS | Angular gyrus | H3K27ac |
| CNS | Angular gyrus | H3K4me1 |
| CNS | Angular gyrus | H3K4me3 |
| CNS | Angular gyrus | H3K9ac |
| CNS | Anterior caudate | H3K27ac |
| CNS | Anterior caudate | H3K4me1 |
| CNS | Anterior caudate | H3K4me3 |
| CNS | Anterior caudate | H3K9ac |
| CNS | Cingulate gyrus | H3K27ac |
| CNS | Cingulate gyrus | H3K4me1 |
| CNS | Cingulate gyrus | H3K4me3 |
| CNS | Cingulate gyrus | H3K9ac |
| CNS | Fetal brain | H3K4me1 |
| CNS | Fetal brain | H3K4me3 |
| CNS | Fetal brain | H3K9ac |
| CNS | Fetal thymus | H3K4me1 |
| CNS | Fetal thymus | H3K4me3 |
| CNS | Germinal matrix | H3K4me3 |
| CNS | Hippocampus middle | H3K27ac |
| CNS | Hippocampus middle | H3K4me1 |
| CNS | Hippocampus middle | H3K4me3 |
| CNS | Hippocampus middle | H3K9ac |
| CNS | Inferior temporal lobe | H3K27ac |
| CNS | Inferior temporal lobe | H3K4me1 |
| CNS | Inferior temporal lobe | H3K4me3 |
| CNS | Inferior temporal lobe | H3K9ac |
| CNS | Mid frontal lobe | H3K27ac |
| CNS | Mid frontal lobe | H3K4me1 |
| CNS | Mid frontal lobe | H3K4me3 |
| CNS | Mid frontal lobe | H3K9ac |
| CNS | Neurosphere | H3K27ac |
| CNS | Substantia nigra | H3K27ac |
| CNS | Substantia nigra | H3K4me1 |
| CNS | Substantia nigra | H3K4me3 |
| CNS | Substantia nigra | H3K9ac |

**Table S3 (continued). List of available cell-type-specific histone modification in cells located in either the central nervous system or immune system**

| Tissue | Cell-type | Histone modification |
| --- | --- | --- |
| Immune | CD14 | H3K27ac |
| Immune | CD14, primary | H3K4me1 |
| Immune | CD14, primary | H3K4me3 |
| Immune | CD15, primary | H3K4me1 |
| Immune | CD15, primary | H3K4me3 |
| Immune | CD19 | H3K27ac |
| Immune | CD19, primary, BI | H3K4me1 |
| Immune | CD19, primary, BI | H3K4me3 |
| Immune | CD19, primary, UW | H3K4me1 |
| Immune | CD19, primary, UW | H3K4me3 |
| Immune | CD20 | H3K27ac |
| Immune | CD25-, CD45RA+, naive | H3K27ac |
| Immune | CD25-, IL17-, Th, stim, MACS | H3K27ac |
| Immune | CD25-, IL17+, Th17, stim | H3K27ac |
| Immune | CD25+, CD127-, Treg | H3K27ac |
| Immune | CD25int, CD127+, Tmem | H3K27ac |
| Immune | CD3, primary | H3K27ac |
| Immune | CD3, primary, BI | H3K4me1 |
| Immune | CD3, primary, BI | H3K4me3 |
| Immune | CD3, primary, UW | H3K4me1 |
| Immune | CD3, primary, UW | H3K4me3 |
| Immune | CD34, primary | H3K4me1 |
| Immune | CD34, primary | H3K4me3 |
| Immune | CD4, memory, primary | H3K4me1 |
| Immune | CD4, memory, primary | H3K4me3 |
| Immune | CD4, naive, primary | H3K4me1 |
| Immune | CD4, naive, primary | H3K4me3 |
| Immune | CD4, primary | H3K4me3 |
| Immune | CD4+, CD25-, CD45R0+, memory, primary | H3K4me1 |
| Immune | CD4+, CD25-, CD45R0+, memory, primary | H3K4me3 |
| Immune | CD4+, CD25-, CD45RA+, naive, primary | H3K4me1 |
| Immune | CD4+, CD25-, CD45RA+, naive, primary | H3K4me3 |
| Immune | CD4+, CD25-, IL17-, PMA, Ionomycin, stim, MACS, Th, sprimary | H3K4me1 |
| Immune | CD4+, CD25-, IL17-, PMA, Ionomycin, stim, MACS, Th, sprimary | H3K4me3 |
| Immune | CD4+, CD25-, IL17+, PMA, Ionomycin, stim, Th17, primary | H3K4me1 |
| Immune | CD4+, CD25-, IL17+, PMA, Ionomycin, stim, Th17, primary | H3K4me3 |
| Immune | CD4+, CD25-, Th, primary | H3K4me1 |
| Immune | CD4+, CD25-, Th, primary | H3K4me3 |

**Table S3 (continued). List of available cell-type-specific histone modification in cells located in either the central nervous system or immune system**

| Tissue | Cell-type | Histone modification | |  |
| --- | --- | --- | --- | --- |
| Immune | CD4+, CD25+, CD127-, Treg, primary | | H3K4me1 | |
| Immune | CD4+, CD25+, CD127-, Treg, primary | | H3K4me3 | |
| Immune | CD4+, CD25int, CD127+, Tmem, primary | H3K4me1 | |  |
| Immune | CD4+, CD25int, CD127+, Tmem, primary | H3K4me3 | |  |
| Immune | CD56, primary | H3K4me1 | |  |
| Immune | CD56, primary | H3K4me3 | |  |
| Immune | CD8, memory, primary | H3K4me1 | |  |
| Immune | CD8, memory, primary | H3K4me3 | |  |
| Immune | CD8, naive, primary, BI | H3K4me1 | |  |
| Immune | CD8, naive, primary, BI | H3K4me3 | |  |
| Immune | CD8, naive, primary, UCSF-UBC | H3K4me1 | |  |
| Immune | CD8, naive, primary, UCSF-UBC | H3K4me3 | |  |
| Immune | CD8, naive, primary, UCSF-UBC | H3K9ac | |  |
| Immune | CD8, primary | H3K4me3 | |  |
| Immune | Mobilized, CD34 | H3K27ac | |  |
| Immune | Mobilized, CD34, primary | H3K4me1 | |  |
| Immune | Mobilized, CD34, primary | H3K4me3 | |  |
| Immune | Peripheralblood, mononuclear, primary | H3K4me1 | |  |
| Immune | Peripheralblood, mononuclear, primary | H3K4me3 | |  |
| Immune | Peripheralblood, mononuclear, primary | H3K9ac | |  |
| Immune | Spleen | H3K4me1 | |  |
| Immune | Spleen | H3K4me3 | |  |
| Immune | Th0 | H3K27ac | |  |
| Immune | Th1 | H3K27ac | |  |
| Immune | Th2 | H3K27ac | |  |
| Immune | Thymus | H3K4me1 | |  |
| Immune | Treg, primary | H3K4me3 | |  |

Middle column indicates the cell-type. Column to the right show the histone modification. Column to the left indicate the tissue from which the cell-types originate.

**Table S4a. Coefficient of simultaneously modelling blood and brain eQTLs for age at menarche**

| Category | Coefficient | SE | Z |
| --- | --- | --- | --- |
| base | 1.361e-08 | 1.036e-08 | 1.314 |
| Coding_UCSC | 6.016e-08 | 6.802e-08 | 0.884 |
| Coding_UCSC.extend.500 | -6.928e-08 | 1.922e-08 | -3.605 |
| Conserved_LindbladToh | 3.683e-07 | 6.549e-08 | 5.623*** |
| Conserved_LindbladToh.extend.500 | 5.250e-09 | 1.149e-08 | 0.457 |
| CTCF_Hoffman | 3.638e-09 | 6.952e-08 | 0.052 |
| CTCF_Hoffman.extend.500 | -1.094e-08 | 3.018e-08 | -0.362 |
| DGF_ENCODE | 1.344e-08 | 2.611e-08 | 0.515 |
| DGF_ENCODE.extend.500 | -3.770e-08 | 1.440e-08 | -2.618 |
| DHS_peaks_Trynka | -5.321e-08 | 4.666e-08 | -1.140 |
| DHS_Trynka | -6.883e-08 | 4.258e-08 | -1.617 |
| DHS_Trynka.extend.500 | 4.268e-08 | 1.836e-08 | 2.325* |
| Enhancer_Andersson | -1.514e-08 | 1.431e-07 | -0.106 |
| Enhancer_Andersson.extend.500 | -4.738e-08 | 4.702e-08 | -1.008 |
| Enhancer_Hoffman | 3.465e-08 | 4.560e-08 | 0.760 |
| Enhancer_Hoffman.extend.500 | -2.897e-08 | 3.501e-08 | -0.827 |
| FetalDHS_Trynka | 2.328e-08 | 4.701e-08 | 0.495 |
| FetalDHS_Trynka.extend.500 | -3.126e-08 | 2.175e-08 | -1.437 |
| H3K27ac_Hnisz | -5.597e-09 | 3.947e-08 | -0.142 |
| H3K27ac_Hnisz.extend.500 | -4.049e-09 | 4.185e-08 | -0.097 |
| H3K27ac_PGC2 | -2.419e-09 | 3.459e-08 | -0.070 |
| H3K27ac_PGC2.extend.500 | 5.570e-09 | 3.284e-08 | 0.170 |
| H3K4me1_peaks_Trynka | 4.198e-08 | 2.624e-08 | 1.600 |
| H3K4me1_Trynka | -3.696e-09 | 2.003e-08 | -0.185 |
| H3K4me1_Trynka.extend.500 | 2.149e-08 | 1.486e-08 | 1.446 |

**Table S4a (continued). Coefficient of simultaneously modelling blood and brain eQTLs for age at menarche**

| Category | Coefficient | SE | Z |
| --- | --- | --- | --- |
| H3K4me3_peaks_Trynka | 6.422e-08 | 5.432e-08 | 1.182 |
| H3K4me3_Trynka | -3.084e-08 | 2.912e-08 | -1.059 |
| H3K4me3_Trynka.extend.500 | -1.125e-08 | 1.915e-08 | -0.587 |
| H3K9ac_peaks_Trynka | 5.265e-08 | 5.271e-08 | 0.999 |
| H3K9ac_Trynka | 7.325e-09 | 3.482e-08 | 0.210 |
| H3K9ac_Trynka.extend.500 | 1.865e-08 | 2.041e-08 | 0.914 |
| Intron_UCSC | -3.358e-08 | 8.524e-08 | -0.394 |
| Intron_UCSC.extend.500 | 3.710e-08 | 8.510e-08 | 0.436 |
| PromoterFlanking_Hoffman | 7.269e-09 | 1.075e-07 | 0.068 |
| PromoterFlanking_Hoffman.extend.500 | -3.404e-08 | 3.957e-08 | -0.860 |
| Promoter_UCSC | 8.748e-08 | 1.009e-07 | 0.867 |
| Promoter_UCSC.extend.500 | -5.403e-08 | 8.470e-08 | -0.638 |
| Repressed_Hoffman | 1.690e-08 | 1.532e-08 | 1.103 |
| Repressed_Hoffman.extend.500 | -1.291e-08 | 1.302e-08 | -0.992 |
| SuperEnhancer_Hnisz | -1.228e-07 | 1.325e-07 | -0.927 |
| SuperEnhancer_Hnisz.extend.500 | 1.131e-07 | 1.304e-07 | 0.868 |
| TFBS_ENCODE | 7.179e-08 | 2.729e-08 | 2.631* |
| TFBS_ENCODE.extend.500 | -1.389e-08 | 1.710e-08 | -0.812 |
| Transcribed_Hoffman | 1.678e-08 | 1.245e-08 | 1.348 |
| Transcribed_Hoffman.extend.500 | -7.367e-09 | 9.332e-09 | -0.789 |
| TSS_Hoffman | 1.440e-07 | 9.836e-08 | 1.464 |
| TSS_Hoffman.extend.500 | -1.559e-08 | 5.725e-08 | -0.272 |
| UTR_3_UCSC | -3.036e-08 | 7.968e-08 | -0.381 |
| UTR_3_UCSC.extend.500 | -1.738e-08 | 3.464e-08 | -0.502 |
| UTR_5_UCSC | 4.460e-08 | 1.021e-07 | 0.437 |
| UTR_5_UCSC.extend.500 | -3.561e-08 | 2.855e-08 | -1.247 |

**Table S4a. Coefficient of simultaneously modelling blood and brain eQTLs for age at menarche**

| Category | Coefficient | SE | Z |
| --- | --- | --- | --- |
| WeakEnhancer_Hoffman | -6.874e-08 | 7.390e-08 | -0.930 |
| WeakEnhancer_Hoffman.extend.500 | 3.201e-08 | 3.476e-08 | 0.921 |
| eQTL_blood_Jansen | 3.836e-08 | 3.842e-08 | 0.999 |
| eQTL_blood_Jansen.extend.100 | -3.676e-08 | 5.897e-08 | -0.623 |
| eQTL_blood_Jansen.extend.500 | 8.631e-09 | 2.302e-08 | 0.375 |
| eQTL_blood_Jansen.extension.GeneCentric | -8.127e-07 | 6.287e-07 | -1.293 |
| eQTL_blood_Jansen.extension.GeneCentric.extension.100 | 8.168e-07 | 7.499e-07 | 1.089 |
| eQTL_blood_Jansen.extension.GeneCentric.extension.500 | -6.872e-09 | 2.143e-07 | -0.032 |
| eQTL_brain_Ramasamy | 4.528e-08 | 4.178e-08 | 1.084 |
| eQTL_brain_Ramasamy.extend.100 | -4.580e-08 | 6.242e-08 | -0.734 |
| eQTL_brain_Ramasamy.extend.500 | 4.026e-09 | 2.263e-08 | 0.178 |
| eQTL_brain_Ramasamy.extension.GeneCentric | -8.155e-08 | 4.480e-07 | -0.182 |
| eQTL_brain_Ramasamy.extension.GeneCentric.extension.100 | 1.285e-07 | 5.438e-07 | 0.236 |
| eQTL_brain_Ramasamy.extension.GeneCentric.extension.500 | -4.062e-08 | 1.609e-07 | -0.252 |

From left to right, columns indicate 1) annotation, 2) the corresponding coefficient, 3) standard error of the coefficient, and 4) the Z-score of the coefficient. One asterisk indicates categories reaching nomial significance (*p*<0.05). Three asterisks indicate categories passing Bonferroni correction for multiple testing.

**Table S4b. Coefficient of simultaneously modelling blood and brain eQTLs for BMI**

| Category | Coefficient | SE | Z |
| --- | --- | --- | --- |
| base | -9.740e-09 | 9.691e-09 | -1.005 |
| Coding_UCSC | -3.497e-08 | 6.190e-08 | -0.565 |
| Coding_UCSC.extend.500 | -1.086e-09 | 1.696e-08 | -0.064 |
| Conserved_LindbladToh | 4.426e-07 | 6.088e-08 | 7.270*** |
| Conserved_LindbladToh.extend.500 | -2.264e-08 | 1.017e-08 | -2.227 |
| CTCF_Hoffman | -2.911e-08 | 7.312e-08 | -0.398 |
| CTCF_Hoffman.extend.500 | -2.334e-08 | 3.439e-08 | -0.679 |
| DGF_ENCODE | -4.550e-08 | 3.086e-08 | -1.474 |
| DGF_ENCODE.extend.500 | -2.367e-09 | 1.485e-08 | -0.159 |
| DHS_peaks_Trynka | 4.424e-08 | 3.997e-08 | 1.107 |
| DHS_Trynka | -1.061e-07 | 4.304e-08 | -2.465 |
| DHS_Trynka.extend.500 | 4.199e-08 | 2.859e-08 | 1.469 |
| Enhancer_Andersson | -1.264e-08 | 1.247e-07 | -0.101 |
| Enhancer_Andersson.extend.500 | -5.803e-08 | 4.641e-08 | -1.251 |
| Enhancer_Hoffman | 7.085e-08 | 5.493e-08 | 1.290 |
| Enhancer_Hoffman.extend.500 | -3.763e-08 | 4.021e-08 | -0.936 |
| FetalDHS_Trynka | -2.230e-08 | 4.244e-08 | -0.525 |
| FetalDHS_Trynka.extend.500 | 1.945e-08 | 1.727e-08 | 1.127 |
| H3K27ac_Hnisz | 2.067e-09 | 3.085e-08 | 0.067 |
| H3K27ac_Hnisz.extend.500 | -6.406e-09 | 3.110e-08 | -0.206 |
| H3K27ac_PGC2 | -2.183e-09 | 2.832e-08 | -0.077 |
| H3K27ac_PGC2.extend.500 | 1.209e-08 | 2.950e-08 | 0.410 |
| H3K4me1_peaks_Trynka | 1.554e-08 | 2.392e-08 | 0.650 |
| H3K4me1_Trynka | -3.629e-09 | 2.245e-08 | -0.162 |
| H3K4me1_Trynka.extend.500 | 8.687e-09 | 1.924e-08 | 0.451 |

**Table S4b (continued). Coefficient of simultaneously modelling blood and brain eQTLs for BMI**

| Category | Coefficient | SE | Z |
| --- | --- | --- | --- |
| H3K4me3_peaks_Trynka | 3.373e-08 | 4.738e-08 | 0.712 |
| H3K4me3_Trynka | -2.051e-08 | 2.690e-08 | -0.763 |
| H3K4me3_Trynka.extend.500 | 2.763e-09 | 1.463e-08 | 0.189 |
| H3K9ac_peaks_Trynka | 1.719e-07 | 5.650e-08 | 3.042** |
| H3K9ac_Trynka | -5.133e-08 | 3.399e-08 | -1.510 |
| H3K9ac_Trynka.extend.500 | 1.392e-08 | 1.956e-08 | 0.711 |
| Intron_UCSC | 1.704e-08 | 7.794e-08 | 0.219 |
| Intron_UCSC.extend.500 | -1.259e-08 | 7.781e-08 | -0.162 |
| PromoterFlanking_Hoffman | -1.733e-08 | 7.644e-08 | -0.227 |
| PromoterFlanking_Hoffman.extend.500 | -1.210e-08 | 3.983e-08 | -0.304 |
| Promoter_UCSC | 8.464e-08 | 9.107e-08 | 0.929 |
| Promoter_UCSC.extend.500 | -7.495e-08 | 7.764e-08 | -0.965 |
| Repressed_Hoffman | -7.559e-09 | 1.372e-08 | -0.551 |
| Repressed_Hoffman.extend.500 | 1.542e-08 | 1.203e-08 | 1.281 |
| SuperEnhancer_Hnisz | -2.105e-07 | 1.098e-07 | -1.916 |
| SuperEnhancer_Hnisz.extend.500 | 2.029e-07 | 1.095e-07 | 1.853* |
| TFBS_ENCODE | 6.762e-08 | 3.082e-08 | 2.194* |
| TFBS_ENCODE.extend.500 | -2.049e-08 | 1.601e-08 | -1.280 |
| Transcribed_Hoffman | 1.049e-08 | 1.330e-08 | 0.789 |
| Transcribed_Hoffman.extend.500 | 1.121e-09 | 1.037e-08 | 0.108 |
| TSS_Hoffman | -2.960e-08 | 8.503e-08 | -0.348 |
| TSS_Hoffman.extend.500 | 2.122e-08 | 6.034e-08 | 0.352 |
| UTR_3_UCSC | 1.118e-07 | 7.793e-08 | 1.435 |
| UTR_3_UCSC.extend.500 | -3.289e-08 | 3.050e-08 | -1.079 |
| UTR_5_UCSC | 6.889e-08 | 8.341e-08 | 0.826 |
| UTR_5_UCSC.extend.500 | -1.507e-08 | 2.180e-08 | -0.691 |

**Table S4b (continued). Coefficient of simultaneously modelling blood and brain eQTLs for BMI**

| Category | Coefficient | SE | Z |
| --- | --- | --- | --- |
| WeakEnhancer_Hoffman | 1.228e-08 | 7.805e-08 | 0.157 |
| WeakEnhancer_Hoffman.extend.500 | 5.055e-08 | 3.691e-08 | 1.369 |
| eQTL_blood_Jansen | 1.014e-07 | 5.277e-08 | 1.921* |
| eQTL_blood_Jansen.extend.100 | -1.415e-07 | 9.289e-08 | -1.524 |
| eQTL_blood_Jansen.extend.500 | 4.392e-08 | 4.197e-08 | 1.047 |
| eQTL_blood_Jansen.extension.GeneCentric | 1.862e-07 | 3.887e-07 | 0.479 |
| eQTL_blood_Jansen.extension.GeneCentric.extension.100 | -1.042e-07 | 4.596e-07 | -0.227 |
| eQTL_blood_Jansen.extension.GeneCentric.extension.500 | -8.742e-08 | 1.738e-07 | -0.503 |
| eQTL_brain_Ramasamy | 4.015e-08 | 3.824e-08 | 1.050 |
| eQTL_brain_Ramasamy.extend.100 | -3.007e-08 | 5.693e-08 | -0.528 |
| eQTL_brain_Ramasamy.extend.500 | -6.038e-09 | 2.025e-08 | -0.298 |
| eQTL_brain_Ramasamy.extension.GeneCentric | -2.185e-07 | 5.981e-07 | -0.365 |
| eQTL_brain_Ramasamy.extension.GeneCentric.extension.100 | 1.883e-07 | 6.801e-07 | 0.277 |
| eQTL_brain_Ramasamy.extension.GeneCentric.extension.500 | 2.669e-08 | 1.522e-07 | 0.175 |

From left to right, columns indicate 1) annotation, 2) the corresponding coefficient, 3) standard error of the coefficient, and 4) the Z-score of the coefficient. One asterisk indicates categories reaching nomial significance (*p*<0.05). Two asterisks indicate categories passing FDR correction for multiple testing (α=0.05). Three asterisks indicate categories passing Bonferroni correction for multiple testing.

**Table S4c. Coefficient of simultaneously modelling blood and brain eQTLs for coronary artery disease**

| Category | Coefficient | SE | Z |
| --- | --- | --- | --- |
| base | -7.959e-09 | 1.201e-08 | -0.663 |
| Coding_UCSC | -1.895e-08 | 8.986e-08 | -0.211 |
| Coding_UCSC.extend.500 | 3.759e-08 | 2.604e-08 | 1.444 |
| Conserved_LindbladToh | 8.417e-08 | 6.032e-08 | 1.395 |
| Conserved_LindbladToh.extend.500 | -8.764e-09 | 1.159e-08 | -0.756 |
| CTCF_Hoffman | -2.526e-08 | 8.940e-08 | -0.283 |
| CTCF_Hoffman.extend.500 | -3.317e-08 | 4.213e-08 | -0.787 |
| DGF_ENCODE | 7.864e-08 | 3.801e-08 | 2.069* |
| DGF_ENCODE.extend.500 | -1.297e-08 | 1.567e-08 | -0.828 |
| DHS_peaks_Trynka | -2.415e-08 | 6.241e-08 | -0.387 |
| DHS_Trynka | 2.397e-09 | 5.602e-08 | 0.043 |
| DHS_Trynka.extend.500 | 5.653e-09 | 1.989e-08 | 0.284 |
| Enhancer_Andersson | -2.421e-07 | 2.084e-07 | -1.161 |
| Enhancer_Andersson.extend.500 | 3.455e-08 | 6.752e-08 | 0.512 |
| Enhancer_Hoffman | 7.899e-08 | 5.792e-08 | 1.364 |
| Enhancer_Hoffman.extend.500 | -4.018e-08 | 4.197e-08 | -0.957 |
| FetalDHS_Trynka | -1.071e-08 | 6.038e-08 | -0.177 |
| FetalDHS_Trynka.extend.500 | 1.687e-08 | 2.289e-08 | 0.737 |
| H3K27ac_Hnisz | -2.350e-08 | 4.304e-08 | -0.546 |
| H3K27ac_Hnisz.extend.500 | 1.675e-08 | 4.251e-08 | 0.394 |
| H3K27ac_PGC2 | -3.256e-08 | 3.858e-08 | -0.844 |
| H3K27ac_PGC2.extend.500 | 3.267e-08 | 3.451e-08 | 0.947 |
| H3K4me1_peaks_Trynka | -2.721e-09 | 2.969e-08 | -0.092 |
| H3K4me1_Trynka | -2.602e-08 | 2.447e-08 | -1.063 |
| H3K4me1_Trynka.extend.500 | 2.824e-08 | 1.776e-08 | 1.590 |

**Table S4c (continued). Coefficient of simultaneously modelling blood and brain eQTLs for coronary artery disease**

| Category | Coefficient | SE | Z |
| --- | --- | --- | --- |
| H3K4me3_peaks_Trynka | 4.807e-08 | 7.492e-08 | 0.642 |
| H3K4me3_Trynka | -9.513e-08 | 3.390e-08 | -2.806 |
| H3K4me3_Trynka.extend.500 | 6.362e-08 | 2.463e-08 | 2.583* |
| H3K9ac_peaks_Trynka | 7.514e-08 | 7.266e-08 | 1.034 |
| H3K9ac_Trynka | 5.128e-08 | 3.806e-08 | 1.348 |
| H3K9ac_Trynka.extend.500 | -3.293e-09 | 2.510e-08 | -0.131 |
| Intron_UCSC | 2.756e-08 | 1.073e-07 | 0.257 |
| Intron_UCSC.extend.500 | -2.819e-08 | 1.068e-07 | -0.264 |
| PromoterFlanking_Hoffman | -4.174e-08 | 1.282e-07 | -0.326 |
| PromoterFlanking_Hoffman.extend.500 | 1.657e-08 | 5.505e-08 | 0.301 |
| Promoter_UCSC | 9.583e-08 | 1.279e-07 | 0.749 |
| Promoter_UCSC.extend.500 | -1.040e-07 | 1.046e-07 | -0.994 |
| Repressed_Hoffman | 1.486e-08 | 1.588e-08 | 0.936 |
| Repressed_Hoffman.extend.500 | -7.192e-09 | 1.303e-08 | -0.552 |
| SuperEnhancer_Hnisz | 2.535e-08 | 1.964e-07 | 0.129 |
| SuperEnhancer_Hnisz.extend.500 | -2.322e-08 | 1.961e-07 | -0.118 |
| TFBS_ENCODE | -8.727e-09 | 4.240e-08 | -0.206 |
| TFBS_ENCODE.extend.500 | -1.148e-08 | 2.076e-08 | -0.553 |
| Transcribed_Hoffman | 2.128e-09 | 1.414e-08 | 0.151 |
| Transcribed_Hoffman.extend.500 | -1.574e-10 | 1.184e-08 | -0.013 |
| TSS_Hoffman | 1.383e-07 | 1.114e-07 | 1.241 |
| TSS_Hoffman.extend.500 | -7.133e-08 | 7.323e-08 | -0.974 |
| UTR_3_UCSC | -1.138e-08 | 1.099e-07 | -0.103 |
| UTR_3_UCSC.extend.500 | 4.898e-08 | 6.698e-08 | 0.731 |
| UTR_5_UCSC | -1.522e-08 | 1.157e-07 | -0.131 |
| UTR_5_UCSC.extend.500 | -5.668e-08 | 3.885e-08 | -1.459 |

**Table S4c (continued). Coefficient of simultaneously modelling blood and brain eQTLs for coronary artery disease**

| Category | Coefficient | SE | Z |
| --- | --- | --- | --- |
| WeakEnhancer_Hoffman | -2.324e-08 | 8.721e-08 | -0.266 |
| WeakEnhancer_Hoffman.extend.500 | 3.009e-08 | 4.481e-08 | 0.672 |
| eQTL_blood_Jansen | 1.408e-08 | 3.756e-08 | 0.375 |
| eQTL_blood_Jansen.extend.100 | -1.094e-08 | 5.347e-08 | -0.205 |
| eQTL_blood_Jansen.extend.500 | 2.202e-09 | 1.925e-08 | 0.114 |
| eQTL_blood_Jansen.extension.GeneCentric | 3.804e-07 | 5.661e-07 | 0.672 |
| eQTL_blood_Jansen.extension.GeneCentric.extension.100 | -5.203e-07 | 6.968e-07 | -0.747 |
| eQTL_blood_Jansen.extension.GeneCentric.extension.500 | 1.361e-07 | 2.648e-07 | 0.514 |
| eQTL_brain_Ramasamy | -2.465e-09 | 4.267e-08 | -0.058 |
| eQTL_brain_Ramasamy.extend.100 | 1.923e-08 | 6.443e-08 | 0.298 |
| eQTL_brain_Ramasamy.extend.500 | -1.803e-08 | 2.332e-08 | -0.773 |
| eQTL_brain_Ramasamy.extension.GeneCentric | -2.762e-07 | 7.329e-07 | -0.377 |
| eQTL_brain_Ramasamy.extension.GeneCentric.extension.100 | 2.804e-07 | 8.614e-07 | 0.326 |
| eQTL_brain_Ramasamy.extension.GeneCentric.extension.500 | -1.036e-08 | 2.375e-07 | -0.044 |

From left to right, columns indicate 1) annotation, 2) the corresponding coefficient, 3) standard error of the coefficient, and 4) the Z-score of the coefficient. One asterisk indicates categories reaching nomial significance (*p*<0.05).

**Table S4d. Coefficient of simultaneously modelling blood and brain eQTLs for Crohn’s disease**

| Category | Coefficient | SE | Z |
| --- | --- | --- | --- |
| base | -8.618e-08 | 6.975e-08 | -1.236 |
| Coding_UCSC | 6.666e-07 | 5.387e-07 | 1.238 |
| Coding_UCSC.extend.500 | 1.642e-07 | 1.468e-07 | 1.119 |
| Conserved_LindbladToh | -1.205e-07 | 3.425e-07 | -0.352 |
| Conserved_LindbladToh.extend.500 | 1.339e-07 | 5.823e-08 | 2.299* |
| CTCF_Hoffman | -6.934e-07 | 5.785e-07 | -1.199 |
| CTCF_Hoffman.extend.500 | -2.495e-07 | 2.485e-07 | -1.004 |
| DGF_ENCODE | 7.063e-07 | 2.253e-07 | 3.135* |
| DGF_ENCODE.extend.500 | 5.341e-08 | 8.327e-08 | 0.641 |
| DHS_peaks_Trynka | 5.145e-07 | 2.802e-07 | 1.836* |
| DHS_Trynka | -1.138e-06 | 2.731e-07 | -4.166 |
| DHS_Trynka.extend.500 | -1.981e-08 | 1.024e-07 | -0.193 |
| Enhancer_Andersson | 1.648e-06 | 1.271e-06 | 1.296 |
| Enhancer_Andersson.extend.500 | 1.572e-06 | 5.465e-07 | 2.876* |
| Enhancer_Hoffman | -4.299e-07 | 3.436e-07 | -1.251 |
| Enhancer_Hoffman.extend.500 | 1.882e-07 | 2.404e-07 | 0.783 |
| FetalDHS_Trynka | 5.782e-07 | 3.037e-07 | 1.904* |
| FetalDHS_Trynka.extend.500 | -1.049e-08 | 1.142e-07 | -0.092 |
| H3K27ac_Hnisz | 1.148e-07 | 2.203e-07 | 0.521 |
| H3K27ac_Hnisz.extend.500 | -1.112e-07 | 2.210e-07 | -0.503 |
| H3K27ac_PGC2 | 6.577e-08 | 2.131e-07 | 0.309 |
| H3K27ac_PGC2.extend.500 | -3.089e-08 | 1.836e-07 | -0.168 |
| H3K4me1_peaks_Trynka | 2.966e-09 | 1.637e-07 | 0.018 |
| H3K4me1_Trynka | -1.845e-08 | 1.450e-07 | -0.127 |
| H3K4me1_Trynka.extend.500 | 4.197e-08 | 9.680e-08 | 0.434 |

**Table S4d (continued). Coefficient of simultaneously modelling blood and brain eQTLs for Crohn’s disease**

| Category | Coefficient | SE | Z |
| --- | --- | --- | --- |
| H3K4me3_peaks_Trynka | 1.989e-07 | 5.162e-07 | 0.385 |
| H3K4me3_Trynka | 4.459e-07 | 2.898e-07 | 1.539 |
| H3K4me3_Trynka.extend.500 | -1.639e-07 | 1.379e-07 | -1.188 |
| H3K9ac_peaks_Trynka | -1.563e-07 | 3.748e-07 | -0.417 |
| H3K9ac_Trynka | -5.514e-07 | 2.069e-07 | -2.665 |
| H3K9ac_Trynka.extend.500 | 1.301e-07 | 1.426e-07 | 0.912 |
| Intron_UCSC | -4.248e-07 | 8.945e-07 | -0.475 |
| Intron_UCSC.extend.500 | 3.846e-07 | 8.949e-07 | 0.430 |
| PromoterFlanking_Hoffman | -1.199e-06 | 7.549e-07 | -1.589 |
| PromoterFlanking_Hoffman.extend.500 | -1.516e-07 | 3.252e-07 | -0.466 |
| Promoter_UCSC | -7.775e-08 | 7.802e-07 | -0.100 |
| Promoter_UCSC.extend.500 | -4.691e-08 | 6.849e-07 | -0.069 |
| Repressed_Hoffman | -1.278e-07 | 8.139e-08 | -1.570 |
| Repressed_Hoffman.extend.500 | 9.657e-08 | 6.453e-08 | 1.496 |
| SuperEnhancer_Hnisz | -1.162e-06 | 1.048e-06 | -1.109 |
| SuperEnhancer_Hnisz.extend.500 | 1.206e-06 | 1.040e-06 | 1.159 |
| TFBS_ENCODE | 1.664e-07 | 2.416e-07 | 0.689 |
| TFBS_ENCODE.extend.500 | 3.332e-08 | 1.002e-07 | 0.332 |
| Transcribed_Hoffman | -9.830e-08 | 9.813e-08 | -1.002 |
| Transcribed_Hoffman.extend.500 | 8.349e-08 | 6.327e-08 | 1.320 |
| TSS_Hoffman | -4.488e-07 | 8.038e-07 | -0.558 |
| TSS_Hoffman.extend.500 | 1.036e-06 | 5.101e-07 | 2.031* |
| UTR_3_UCSC | -8.085e-07 | 5.503e-07 | -1.469 |
| UTR_3_UCSC.extend.500 | 3.682e-07 | 3.416e-07 | 1.078 |
| UTR_5_UCSC | -8.693e-08 | 8.515e-07 | -0.102 |
| UTR_5_UCSC.extend.500 | -1.813e-07 | 2.478e-07 | -0.732 |

**Table S4d (continued). Coefficient of simultaneously modelling blood and brain eQTLs for Crohn’s disease**

| Category | Coefficient | SE | Z |
| --- | --- | --- | --- |
| WeakEnhancer_Hoffman | 6.026e-07 | 5.767e-07 | 1.045 |
| WeakEnhancer_Hoffman.extend.500 | -1.894e-07 | 2.580e-07 | -0.734 |
| eQTL_blood_Jansen | 3.503e-07 | 2.683e-07 | 1.306 |
| eQTL_blood_Jansen.extend.100 | -3.541e-07 | 3.904e-07 | -0.907 |
| eQTL_blood_Jansen.extend.500 | 6.069e-08 | 1.401e-07 | 0.433 |
| eQTL_blood_Jansen.extension.GeneCentric | 2.223e-06 | 3.737e-06 | 0.595 |
| eQTL_blood_Jansen.extension.GeneCentric.extension.100 | -4.664e-06 | 4.570e-06 | -1.021 |
| eQTL_blood_Jansen.extension.GeneCentric.extension.500 | 2.434e-06 | 1.756e-06 | 1.386 |
| eQTL_brain_Ramasamy | 8.332e-07 | 3.279e-07 | 2.541* |
| eQTL_brain_Ramasamy.extend.100 | -1.086e-06 | 4.922e-07 | -2.207 |
| eQTL_brain_Ramasamy.extend.500 | 2.739e-07 | 1.769e-07 | 1.548 |
| eQTL_brain_Ramasamy.extension.GeneCentric | -1.334e-07 | 1.986e-07 | -0.672 |
| eQTL_brain_Ramasamy.extension.GeneCentric.extension.100 | 2.452e-06 | 1.350e-06 | 1.817* |
| eQTL_brain_Ramasamy.extension.GeneCentric.extension.500 | -2.323e-06 | 1.433e-06 | -1.621 |

From left to right, columns indicate 1) annotation, 2) the corresponding coefficient, 3) standard error of the coefficient, and 4) the Z-score of the coefficient. One asterisk indicates categories reaching nomial significance (*p*<0.05).

**Table S4e. Coefficient of simultaneously modelling blood and brain eQTLs for educational attainment**

| Category | Coefficient | SE | Z |
| --- | --- | --- | --- |
| base | 5.700e-09 | 4.164e-09 | 1.369 |
| Coding_UCSC | 5.283e-09 | 3.007e-08 | 0.176 |
| Coding_UCSC.extend.500 | -1.870e-08 | 9.156e-09 | -2.042 |
| Conserved_LindbladToh | 2.318e-07 | 3.393e-08 | 6.833*** |
| Conserved_LindbladToh.extend.500 | 5.875e-09 | 4.547e-09 | 1.292 |
| CTCF_Hoffman | -5.135e-08 | 2.576e-08 | -1.993 |
| CTCF_Hoffman.extend.500 | 2.028e-08 | 1.246e-08 | 1.628 |
| DGF_ENCODE | -2.723e-09 | 1.272e-08 | -0.214 |
| DGF_ENCODE.extend.500 | -8.967e-09 | 6.939e-09 | -1.292 |
| DHS_peaks_Trynka | 6.422e-09 | 1.972e-08 | 0.326 |
| DHS_Trynka | -3.329e-08 | 1.748e-08 | -1.905 |
| DHS_Trynka.extend.500 | 9.413e-09 | 7.533e-09 | 1.250 |
| Enhancer_Andersson | -3.271e-08 | 6.022e-08 | -0.543 |
| Enhancer_Andersson.extend.500 | 3.753e-08 | 2.767e-08 | 1.356 |
| Enhancer_Hoffman | -1.016e-08 | 1.688e-08 | -0.602 |
| Enhancer_Hoffman.extend.500 | -5.841e-09 | 1.409e-08 | -0.415 |
| FetalDHS_Trynka | 1.779e-08 | 1.912e-08 | 0.930 |
| FetalDHS_Trynka.extend.500 | 7.385e-09 | 7.229e-09 | 1.022 |
| H3K27ac_Hnisz | 2.207e-08 | 1.793e-08 | 1.230 |
| H3K27ac_Hnisz.extend.500 | -2.532e-08 | 1.865e-08 | -1.357 |
| H3K27ac_PGC2 | -1.047e-09 | 1.300e-08 | -0.080 |
| H3K27ac_PGC2.extend.500 | -5.678e-09 | 1.203e-08 | -0.472 |
| H3K4me1_peaks_Trynka | 1.563e-08 | 1.049e-08 | 1.489 |
| H3K4me1_Trynka | 3.124e-09 | 9.162e-09 | 0.341 |
| H3K4me1_Trynka.extend.500 | 1.167e-09 | 7.683e-09 | 0.152 |

**Table S4e (continued). Coefficient of simultaneously modelling blood and brain eQTLs for educational attainment**

| Category | Coefficient | SE | Z |
| --- | --- | --- | --- |
| H3K4me3_peaks_Trynka | -9.100e-10 | 2.025e-08 | -0.045 |
| H3K4me3_Trynka | -1.988e-09 | 1.169e-08 | -0.170 |
| H3K4me3_Trynka.extend.500 | -5.636e-09 | 7.597e-09 | -0.742 |
| H3K9ac_peaks_Trynka | 5.193e-08 | 2.097e-08 | 2.476* |
| H3K9ac_Trynka | -1.713e-08 | 1.453e-08 | -1.179 |
| H3K9ac_Trynka.extend.500 | 1.717e-08 | 9.007e-09 | 1.906* |
| Intron_UCSC | 8.658e-08 | 3.501e-08 | 2.473* |
| Intron_UCSC.extend.500 | -8.262e-08 | 3.509e-08 | -2.355 |
| PromoterFlanking_Hoffman | -6.245e-08 | 4.891e-08 | -1.277 |
| PromoterFlanking_Hoffman.extend.500 | 1.024e-09 | 2.094e-08 | 0.049 |
| Promoter_UCSC | -2.244e-08 | 4.505e-08 | -0.498 |
| Promoter_UCSC.extend.500 | 1.866e-08 | 3.690e-08 | 0.506 |
| Repressed_Hoffman | 4.759e-09 | 7.216e-09 | 0.659 |
| Repressed_Hoffman.extend.500 | -3.310e-09 | 6.157e-09 | -0.538 |
| SuperEnhancer_Hnisz | 5.455e-08 | 4.949e-08 | 1.102 |
| SuperEnhancer_Hnisz.extend.500 | -5.819e-08 | 4.921e-08 | -1.182 |
| TFBS_ENCODE | 1.635e-09 | 1.274e-08 | 0.128 |
| TFBS_ENCODE.extend.500 | -2.095e-09 | 7.108e-09 | -0.295 |
| Transcribed_Hoffman | 2.354e-09 | 5.158e-09 | 0.456 |
| Transcribed_Hoffman.extend.500 | 2.044e-09 | 4.264e-09 | 0.479 |
| TSS_Hoffman | 2.119e-08 | 3.402e-08 | 0.623 |
| TSS_Hoffman.extend.500 | 2.128e-08 | 2.390e-08 | 0.890 |
| UTR_3_UCSC | 5.857e-08 | 3.051e-08 | 1.920* |
| UTR_3_UCSC.extend.500 | -3.446e-08 | 1.288e-08 | -2.676 |
| UTR_5_UCSC | 1.489e-08 | 3.950e-08 | 0.377 |
| UTR_5_UCSC.extend.500 | 6.014e-09 | 1.233e-08 | 0.488 |

**Table S4e (continued). Coefficient of simultaneously modelling blood and brain eQTLs for educational attainment**

| Category | Coefficient | SE | Z |
| --- | --- | --- | --- |
| WeakEnhancer_Hoffman | 1.614e-08 | 2.969e-08 | 0.544 |
| WeakEnhancer_Hoffman.extend.500 | 1.476e-08 | 1.318e-08 | 1.120 |
| eQTL_blood_Jansen | 1.851e-08 | 1.352e-08 | 1.369 |
| eQTL_blood_Jansen.extend.100 | -9.418e-09 | 1.986e-08 | -0.474 |
| eQTL_blood_Jansen.extend.500 | -6.430e-09 | 7.341e-09 | -0.876 |
| eQTL_blood_Jansen.extension.GeneCentric | 4.228e-07 | 1.667e-07 | 2.536* |
| eQTL_blood_Jansen.extension.GeneCentric.extension.100 | -5.365e-07 | 2.179e-07 | -2.462 |
| eQTL_blood_Jansen.extension.GeneCentric.extension.500 | 1.152e-07 | 9.724e-08 | 1.185 |
| eQTL_brain_Ramasamy | 4.423e-08 | 1.542e-08 | 2.869* |
| eQTL_brain_Ramasamy.extend.100 | -3.690e-08 | 2.190e-08 | -1.685 |
| eQTL_brain_Ramasamy.extend.500 | -2.453e-09 | 8.276e-09 | -0.296 |
| eQTL_brain_Ramasamy.extension.GeneCentric | -2.896e-08 | 8.774e-08 | -0.330 |
| eQTL_brain_Ramasamy.extension.GeneCentric.extension.100 | -1.429e-07 | 1.731e-07 | -0.826 |
| eQTL_brain_Ramasamy.extension.GeneCentric.extension.500 | 1.720e-07 | 1.517e-07 | 1.134 |

From left to right, columns indicate 1) annotation, 2) the corresponding coefficient, 3) standard error of the coefficient, and 4) the Z-score of the coefficient. One asterisk indicates categories reaching nomial significance (*p*<0.05). Three asterisks indicate categories passing Bonferroni correction for multiple testing.

**Table S4f. Coefficient of simultaneously modelling blood and brain eQTLs for height**

| Category | Coefficient | SE | Z |
| --- | --- | --- | --- |
| base | 1.939e-08 | 2.439e-08 | 0.795 |
| Coding_UCSC | 6.795e-08 | 1.215e-07 | 0.559 |
| Coding_UCSC.extend.500 | -3.296e-08 | 3.850e-08 | -0.856 |
| Conserved_LindbladToh | 5.346e-07 | 1.078e-07 | 4.960*** |
| Conserved_LindbladToh.extend.500 | 4.407e-09 | 1.597e-08 | 0.276 |
| CTCF_Hoffman | -1.943e-08 | 1.188e-07 | -0.164 |
| CTCF_Hoffman.extend.500 | -6.039e-09 | 5.218e-08 | -0.116 |
| DGF_ENCODE | 8.308e-08 | 4.904e-08 | 1.694* |
| DGF_ENCODE.extend.500 | 2.615e-08 | 2.050e-08 | 1.276 |
| DHS_peaks_Trynka | 5.187e-08 | 6.848e-08 | 0.757 |
| DHS_Trynka | 6.132e-09 | 5.947e-08 | 0.103 |
| DHS_Trynka.extend.500 | -2.127e-08 | 2.354e-08 | -0.904 |
| Enhancer_Andersson | 1.589e-07 | 2.179e-07 | 0.729 |
| Enhancer_Andersson.extend.500 | -1.873e-07 | 7.104e-08 | -2.636 |
| Enhancer_Hoffman | 6.637e-09 | 9.158e-08 | 0.072 |
| Enhancer_Hoffman.extend.500 | 3.661e-08 | 5.705e-08 | 0.642 |
| FetalDHS_Trynka | -4.994e-08 | 7.429e-08 | -0.672 |
| FetalDHS_Trynka.extend.500 | -2.726e-08 | 2.987e-08 | -0.913 |
| H3K27ac_Hnisz | 6.003e-08 | 5.266e-08 | 1.140 |
| H3K27ac_Hnisz.extend.500 | -5.127e-08 | 4.868e-08 | -1.053 |
| H3K27ac_PGC2 | -5.813e-08 | 6.155e-08 | -0.944 |
| H3K27ac_PGC2.extend.500 | 4.024e-08 | 4.854e-08 | 0.829 |
| H3K4me1_peaks_Trynka | -2.834e-08 | 4.598e-08 | -0.616 |
| H3K4me1_Trynka | 1.050e-07 | 3.224e-08 | 3.256*** |
| H3K4me1_Trynka.extend.500 | -3.355e-08 | 2.242e-08 | -1.497 |

**Table S4f (continued). Coefficient of simultaneously modelling blood and brain eQTLs for height**

| Category | Coefficient | SE | Z |
| --- | --- | --- | --- |
| H3K4me3_peaks_Trynka | -6.976e-08 | 9.905e-08 | -0.704 |
| H3K4me3_Trynka | 4.498e-08 | 5.063e-08 | 0.888 |
| H3K4me3_Trynka.extend.500 | -2.088e-08 | 2.906e-08 | -0.718 |
| H3K9ac_peaks_Trynka | 2.269e-07 | 1.049e-07 | 2.164* |
| H3K9ac_Trynka | 1.145e-07 | 5.527e-08 | 2.072* |
| H3K9ac_Trynka.extend.500 | -3.044e-08 | 3.901e-08 | -0.780 |
| Intron_UCSC | -5.790e-08 | 1.821e-07 | -0.318 |
| Intron_UCSC.extend.500 | 5.833e-08 | 1.825e-07 | 0.320 |
| PromoterFlanking_Hoffman | -5.591e-07 | 1.945e-07 | -2.875 |
| PromoterFlanking_Hoffman.extend.500 | 1.295e-07 | 7.541e-08 | 1.717* |
| Promoter_UCSC | 2.293e-07 | 2.592e-07 | 0.884 |
| Promoter_UCSC.extend.500 | -2.466e-07 | 1.919e-07 | -1.285 |
| Repressed_Hoffman | -3.469e-09 | 2.742e-08 | -0.127 |
| Repressed_Hoffman.extend.500 | -4.962e-09 | 1.908e-08 | -0.260 |
| SuperEnhancer_Hnisz | -1.477e-07 | 2.173e-07 | -0.680 |
| SuperEnhancer_Hnisz.extend.500 | 1.471e-07 | 2.127e-07 | 0.692 |
| TFBS_ENCODE | -4.987e-09 | 4.978e-08 | -0.100 |
| TFBS_ENCODE.extend.500 | 2.877e-08 | 2.305e-08 | 1.248 |
| Transcribed_Hoffman | 5.061e-08 | 3.353e-08 | 1.509 |
| Transcribed_Hoffman.extend.500 | -4.265e-08 | 2.104e-08 | -2.027 |
| TSS_Hoffman | -5.654e-08 | 1.958e-07 | -0.289 |
| TSS_Hoffman.extend.500 | 1.722e-07 | 1.194e-07 | 1.442 |
| UTR_3_UCSC | 3.279e-07 | 1.999e-07 | 1.640 |
| UTR_3_UCSC.extend.500 | 5.807e-09 | 1.283e-07 | 0.045 |
| UTR_5_UCSC | 3.819e-07 | 2.031e-07 | 1.880* |
| UTR_5_UCSC.extend.500 | -1.836e-07 | 5.685e-08 | -3.230 |

**Table S4f (continued). Coefficient of simultaneously modelling blood and brain eQTLs for height**

| Category | Coefficient | SE | Z |
| --- | --- | --- | --- |
| WeakEnhancer_Hoffman | -1.446e-07 | 1.165e-07 | -1.241 |
| WeakEnhancer_Hoffman.extend.500 | -9.584e-08 | 5.325e-08 | -1.800 |
| eQTL_blood_Jansen | 1.961e-07 | 6.521e-08 | 3.008** |
| eQTL_blood_Jansen.extend.100 | -2.396e-07 | 9.733e-08 | -2.462 |
| eQTL_blood_Jansen.extend.500 | 5.700e-08 | 3.684e-08 | 1.547 |
| eQTL_blood_Jansen.extension.GeneCentric | 1.251e-06 | 7.180e-07 | 1.742* |
| eQTL_blood_Jansen.extension.GeneCentric.extension.100 | -1.632e-06 | 9.275e-07 | -1.759 |
| eQTL_blood_Jansen.extension.GeneCentric.extension.500 | 3.588e-07 | 4.578e-07 | 0.784 |
| eQTL_brain_Ramasamy | 1.493e-07 | 7.152e-08 | 2.088* |
| eQTL_brain_Ramasamy.extend.100 | -1.429e-07 | 1.074e-07 | -1.331 |
| eQTL_brain_Ramasamy.extend.500 | 2.075e-08 | 4.132e-08 | 0.502 |
| eQTL_brain_Ramasamy.extension.GeneCentric | 1.479e-08 | 4.245e-08 | 0.348 |
| eQTL_brain_Ramasamy.extension.GeneCentric.extension.100 | 6.570e-08 | 3.399e-07 | 0.193 |
| eQTL_brain_Ramasamy.extension.GeneCentric.extension.500 | -8.607e-08 | 3.451e-07 | -0.249 |

From left to right, columns indicate 1) annotation, 2) the corresponding coefficient, 3) standard error of the coefficient, and 4) the Z-score of the coefficient. One asterisk indicates categories reaching nomial significance (*p*<0.05). Two asterisks indicate categories passing FDR correction for multiple testing (α=0.05). Three asterisks indicate categories passing Bonferroni correction for multiple testing.

**Table S4g. Coefficient of simultaneously modelling blood and brain eQTLs for LDL level**

| Category | Coefficient | SE | Z |
| --- | --- | --- | --- |
| base | -6.780e-09 | 1.422e-08 | -0.477 |
| Coding_UCSC | -1.001e-07 | 1.238e-07 | -0.809 |
| Coding_UCSC.extend.500 | 1.186e-07 | 3.772e-08 | 3.145* |
| Conserved_LindbladToh | 1.151e-07 | 7.793e-08 | 1.477 |
| Conserved_LindbladToh.extend.500 | -1.763e-09 | 1.230e-08 | -0.143 |
| CTCF_Hoffman | 1.194e-07 | 1.235e-07 | 0.967 |
| CTCF_Hoffman.extend.500 | -5.961e-08 | 5.550e-08 | -1.074 |
| DGF_ENCODE | 2.399e-08 | 4.018e-08 | 0.597 |
| DGF_ENCODE.extend.500 | -1.814e-08 | 1.740e-08 | -1.042 |
| DHS_peaks_Trynka | 3.677e-09 | 5.436e-08 | 0.068 |
| DHS_Trynka | -3.039e-08 | 5.568e-08 | -0.546 |
| DHS_Trynka.extend.500 | -7.741e-09 | 2.178e-08 | -0.355 |
| Enhancer_Andersson | -2.984e-07 | 2.308e-07 | -1.293 |
| Enhancer_Andersson.extend.500 | -1.639e-08 | 6.572e-08 | -0.249 |
| Enhancer_Hoffman | 1.069e-07 | 6.908e-08 | 1.547 |
| Enhancer_Hoffman.extend.500 | -4.602e-08 | 4.567e-08 | -1.008 |
| FetalDHS_Trynka | 2.301e-08 | 6.173e-08 | 0.373 |
| FetalDHS_Trynka.extend.500 | 1.744e-08 | 2.084e-08 | 0.837 |
| H3K27ac_Hnisz | -9.122e-08 | 4.594e-08 | -1.986 |
| H3K27ac_Hnisz.extend.500 | 9.364e-08 | 4.481e-08 | 2.090* |
| H3K27ac_PGC2 | 2.630e-08 | 4.939e-08 | 0.533 |
| H3K27ac_PGC2.extend.500 | -3.557e-08 | 4.032e-08 | -0.882 |
| H3K4me1_peaks_Trynka | -2.797e-08 | 4.820e-08 | -0.580 |
| H3K4me1_Trynka | 4.302e-08 | 2.709e-08 | 1.588 |
| H3K4me1_Trynka.extend.500 | -1.292e-08 | 2.266e-08 | -0.570 |

**Table S4g (continued). Coefficient of simultaneously modelling blood and brain eQTLs for LDL level**

| Category | Coefficient | SE | Z |
| --- | --- | --- | --- |
| H3K4me3_peaks_Trynka | -6.674e-08 | 1.114e-07 | -0.599 |
| H3K4me3_Trynka | 2.747e-09 | 4.788e-08 | 0.057 |
| H3K4me3_Trynka.extend.500 | 2.544e-08 | 2.731e-08 | 0.932 |
| H3K9ac_peaks_Trynka | -1.644e-07 | 9.744e-08 | -1.687 |
| H3K9ac_Trynka | 9.689e-08 | 4.608e-08 | 2.102* |
| H3K9ac_Trynka.extend.500 | -1.538e-08 | 3.924e-08 | -0.392 |
| Intron_UCSC | -2.729e-07 | 1.293e-07 | -2.111 |
| Intron_UCSC.extend.500 | 2.662e-07 | 1.300e-07 | 2.047* |
| PromoterFlanking_Hoffman | -7.099e-08 | 1.971e-07 | -0.360 |
| PromoterFlanking_Hoffman.extend.500 | 3.385e-08 | 7.935e-08 | 0.427 |
| Promoter_UCSC | -8.677e-08 | 1.783e-07 | -0.487 |
| Promoter_UCSC.extend.500 | 4.900e-08 | 1.395e-07 | 0.351 |
| Repressed_Hoffman | 1.533e-09 | 1.672e-08 | 0.092 |
| Repressed_Hoffman.extend.500 | 1.057e-08 | 1.300e-08 | 0.813 |
| SuperEnhancer_Hnisz | -6.533e-08 | 1.763e-07 | -0.371 |
| SuperEnhancer_Hnisz.extend.500 | 6.879e-08 | 1.758e-07 | 0.391 |
| TFBS_ENCODE | 8.439e-08 | 6.020e-08 | 1.402 |
| TFBS_ENCODE.extend.500 | -2.214e-08 | 2.485e-08 | -0.891 |
| Transcribed_Hoffman | 1.994e-08 | 1.785e-08 | 1.117 |
| Transcribed_Hoffman.extend.500 | -1.025e-08 | 1.226e-08 | -0.837 |
| TSS_Hoffman | -3.248e-07 | 1.985e-07 | -1.637 |
| TSS_Hoffman.extend.500 | 3.202e-07 | 1.330e-07 | 2.407* |
| UTR_3_UCSC | -1.110e-08 | 1.007e-07 | -0.110 |
| UTR_3_UCSC.extend.500 | -4.811e-08 | 4.377e-08 | -1.099 |
| UTR_5_UCSC | -4.463e-08 | 1.605e-07 | -0.278 |
| UTR_5_UCSC.extend.500 | -9.931e-08 | 6.629e-08 | -1.498 |

**Table S4g (continued). Coefficient of simultaneously modelling blood and brain eQTLs for LDL level**

| Category | Coefficient | SE | Z |
| --- | --- | --- | --- |
| WeakEnhancer_Hoffman | 1.564e-10 | 9.451e-08 | 0.002 |
| WeakEnhancer_Hoffman.extend.500 | 6.593e-09 | 4.436e-08 | 0.149 |
| eQTL_blood_Jansen | 1.073e-07 | 5.780e-08 | 1.857* |
| eQTL_blood_Jansen.extend.100 | -1.350e-07 | 8.562e-08 | -1.577 |
| eQTL_blood_Jansen.extend.500 | 4.110e-08 | 3.100e-08 | 1.326 |
| eQTL_blood_Jansen.extension.GeneCentric | 3.227e-07 | 1.021e-06 | 0.316 |
| eQTL_blood_Jansen.extension.GeneCentric.extension.100 | -3.868e-07 | 1.171e-06 | -0.330 |
| eQTL_blood_Jansen.extension.GeneCentric.extension.500 | 5.902e-08 | 3.886e-07 | 0.152 |
| eQTL_brain_Ramasamy | -1.253e-09 | 5.838e-08 | -0.021 |
| eQTL_brain_Ramasamy.extend.100 | 3.587e-09 | 8.348e-08 | 0.043 |
| eQTL_brain_Ramasamy.extend.500 | -5.711e-09 | 2.842e-08 | -0.201 |
| eQTL_brain_Ramasamy.extension.GeneCentric | -1.548e-07 | 2.646e-06 | -0.059 |
| eQTL_brain_Ramasamy.extension.GeneCentric.extension.100 | -3.856e-07 | 3.090e-06 | -0.125 |
| eQTL_brain_Ramasamy.extension.GeneCentric.extension.500 | 5.421e-07 | 7.429e-07 | 0.730 |

From left to right, columns indicate 1) annotation, 2) the corresponding coefficient, 3) standard error of the coefficient, and 4) the Z-score of the coefficient. One asterisk indicates categories reaching nomial significance (*p*<0.05).

**Table S4h. Coefficient of simultaneously modelling blood and brain eQTLs for rheumatoid arthritis**

| Category | Coefficient | SE | Z |
| --- | --- | --- | --- |
| base | -2.031e-08 | 1.311e-08 | -1.549 |
| Coding_UCSC | 1.261e-07 | 1.632e-07 | 0.773 |
| Coding_UCSC.extend.500 | 6.893e-08 | 3.919e-08 | 1.759* |
| Conserved_LindbladToh | 5.217e-08 | 9.825e-08 | 0.531 |
| Conserved_LindbladToh.extend.500 | 1.032e-08 | 1.639e-08 | 0.630 |
| CTCF_Hoffman | -6.320e-08 | 1.328e-07 | -0.476 |
| CTCF_Hoffman.extend.500 | -5.568e-08 | 5.969e-08 | -0.933 |
| DGF_ENCODE | 1.209e-07 | 6.281e-08 | 1.925* |
| DGF_ENCODE.extend.500 | 1.398e-08 | 2.163e-08 | 0.646 |
| DHS_peaks_Trynka | -7.888e-08 | 8.723e-08 | -0.904 |
| DHS_Trynka | -5.717e-08 | 6.846e-08 | -0.835 |
| DHS_Trynka.extend.500 | 4.201e-09 | 2.787e-08 | 0.151 |
| Enhancer_Andersson | 3.660e-07 | 4.292e-07 | 0.853 |
| Enhancer_Andersson.extend.500 | 2.546e-07 | 1.287e-07 | 1.979* |
| Enhancer_Hoffman | -4.245e-08 | 8.996e-08 | -0.472 |
| Enhancer_Hoffman.extend.500 | 2.120e-08 | 5.562e-08 | 0.381 |
| FetalDHS_Trynka | 9.346e-08 | 7.492e-08 | 1.247 |
| FetalDHS_Trynka.extend.500 | 4.200e-09 | 2.751e-08 | 0.153 |
| H3K27ac_Hnisz | 4.895e-08 | 6.415e-08 | 0.763 |
| H3K27ac_Hnisz.extend.500 | -3.474e-08 | 6.170e-08 | -0.563 |
| H3K27ac_PGC2 | 8.865e-08 | 6.302e-08 | 1.407 |
| H3K27ac_PGC2.extend.500 | -4.600e-08 | 5.196e-08 | -0.885 |
| H3K4me1_peaks_Trynka | -2.683e-08 | 4.168e-08 | -0.644 |
| H3K4me1_Trynka | 1.478e-08 | 3.929e-08 | 0.376 |
| H3K4me1_Trynka.extend.500 | -3.890e-09 | 2.470e-08 | -0.157 |

**Table S4h (continued). Coefficient of simultaneously modelling blood and brain eQTLs for rheumatoid arthritis**

| Category | Coefficient | SE | Z |
| --- | --- | --- | --- |
| H3K4me3_peaks_Trynka | -6.896e-09 | 9.286e-08 | -0.074 |
| H3K4me3_Trynka | 1.177e-07 | 5.222e-08 | 2.253* |
| H3K4me3_Trynka.extend.500 | -5.352e-08 | 3.284e-08 | -1.630 |
| H3K9ac_peaks_Trynka | -2.867e-08 | 8.416e-08 | -0.341 |
| H3K9ac_Trynka | -1.645e-07 | 6.373e-08 | -2.581 |
| H3K9ac_Trynka.extend.500 | 1.336e-09 | 4.097e-08 | 0.033 |
| Intron_UCSC | 7.883e-09 | 1.337e-07 | 0.059 |
| Intron_UCSC.extend.500 | -1.601e-08 | 1.334e-07 | -0.120 |
| PromoterFlanking_Hoffman | -2.319e-07 | 2.684e-07 | -0.864 |
| PromoterFlanking_Hoffman.extend.500 | 5.996e-08 | 1.126e-07 | 0.532 |
| Promoter_UCSC | 3.231e-07 | 2.418e-07 | 1.336 |
| Promoter_UCSC.extend.500 | -2.798e-07 | 1.912e-07 | -1.463 |
| Repressed_Hoffman | 8.950e-09 | 2.046e-08 | 0.437 |
| Repressed_Hoffman.extend.500 | 1.416e-08 | 1.760e-08 | 0.805 |
| SuperEnhancer_Hnisz | -4.972e-07 | 3.711e-07 | -1.340 |
| SuperEnhancer_Hnisz.extend.500 | 5.162e-07 | 3.690e-07 | 1.399 |
| TFBS_ENCODE | 1.168e-07 | 5.943e-08 | 1.966* |
| TFBS_ENCODE.extend.500 | -5.311e-08 | 3.249e-08 | -1.634 |
| Transcribed_Hoffman | 1.488e-08 | 2.279e-08 | 0.653 |
| Transcribed_Hoffman.extend.500 | -9.698e-09 | 1.783e-08 | -0.544 |
| TSS_Hoffman | 3.101e-07 | 2.311e-07 | 1.342 |
| TSS_Hoffman.extend.500 | -3.573e-09 | 1.432e-07 | -0.025 |
| UTR_3_UCSC | -5.989e-08 | 1.664e-07 | -0.360 |
| UTR_3_UCSC.extend.500 | -2.383e-08 | 7.279e-08 | -0.327 |
| UTR_5_UCSC | 1.138e-07 | 2.119e-07 | 0.537 |
| UTR_5_UCSC.extend.500 | -6.505e-08 | 7.088e-08 | -0.918 |

**Table S4h (continued). Coefficient of simultaneously modelling blood and brain eQTLs for rheumatoid arthritis**

| Category | Coefficient | SE | Z |
| --- | --- | --- | --- |
| WeakEnhancer_Hoffman | 8.592e-08 | 1.590e-07 | 0.540 |
| WeakEnhancer_Hoffman.extend.500 | 3.714e-08 | 6.702e-08 | 0.554 |
| eQTL_blood_Jansen | 1.215e-07 | 5.886e-08 | 2.065* |
| eQTL_blood_Jansen.extend.100 | -1.585e-07 | 8.429e-08 | -1.881 |
| eQTL_blood_Jansen.extend.500 | 5.310e-08 | 2.946e-08 | 1.803* |
| eQTL_blood_Jansen.extension.GeneCentric | 2.462e-07 | 1.057e-06 | 0.233 |
| eQTL_blood_Jansen.extension.GeneCentric.extension.100 | -5.916e-07 | 1.271e-06 | -0.465 |
| eQTL_blood_Jansen.extension.GeneCentric.extension.500 | 3.473e-07 | 4.248e-07 | 0.818 |
| eQTL_brain_Ramasamy | 2.423e-07 | 9.197e-08 | 2.634* |
| eQTL_brain_Ramasamy.extend.100 | -3.535e-07 | 1.405e-07 | -2.516 |
| eQTL_brain_Ramasamy.extend.500 | 1.144e-07 | 5.091e-08 | 2.248* |
| eQTL_brain_Ramasamy.extension.GeneCentric | 2.266e-08 | 1.291e-07 | 0.176 |
| eQTL_brain_Ramasamy.extension.GeneCentric.extension.100 | 7.415e-07 | 6.167e-07 | 1.202 |
| eQTL_brain_Ramasamy.extension.GeneCentric.extension.500 | -7.715e-07 | 6.393e-07 | -1.207 |

From left to right, columns indicate 1) annotation, 2) the corresponding coefficient, 3) standard error of the coefficient, and 4) the Z-score of the coefficient. One asterisk indicates categories reaching nomial significance (*p*<0.05).

**Table S4i. Coefficient of simultaneously modelling blood and brain eQTLs for smoking behavior**

| Category | Coefficient | SE | Z |
| --- | --- | --- | --- |
| base | 2.342e-08 | 1.384e-08 | 1.691* |
| Coding_UCSC | 8.441e-08 | 7.678e-08 | 1.099 |
| Coding_UCSC.extend.500 | -1.167e-08 | 2.145e-08 | -0.544 |
| Conserved_LindbladToh | 1.280e-07 | 5.545e-08 | 2.308* |
| Conserved_LindbladToh.extend.500 | 4.416e-09 | 8.302e-09 | 0.532 |
| CTCF_Hoffman | -2.811e-08 | 8.047e-08 | -0.349 |
| CTCF_Hoffman.extend.500 | 2.875e-08 | 3.331e-08 | 0.863 |
| DGF_ENCODE | 3.822e-09 | 3.287e-08 | 0.116 |
| DGF_ENCODE.extend.500 | 9.800e-09 | 1.598e-08 | 0.613 |
| DHS_peaks_Trynka | -1.417e-08 | 5.854e-08 | -0.242 |
| DHS_Trynka | -5.103e-08 | 4.636e-08 | -1.101 |
| DHS_Trynka.extend.500 | 9.388e-09 | 1.907e-08 | 0.492 |
| Enhancer_Andersson | 6.969e-08 | 1.601e-07 | 0.435 |
| Enhancer_Andersson.extend.500 | -1.104e-07 | 6.178e-08 | -1.787 |
| Enhancer_Hoffman | 1.569e-08 | 5.727e-08 | 0.274 |
| Enhancer_Hoffman.extend.500 | 1.188e-08 | 4.158e-08 | 0.286 |
| FetalDHS_Trynka | 6.921e-08 | 5.761e-08 | 1.201 |
| FetalDHS_Trynka.extend.500 | 6.895e-09 | 1.977e-08 | 0.349 |
| H3K27ac_Hnisz | 3.457e-08 | 4.666e-08 | 0.741 |
| H3K27ac_Hnisz.extend.500 | -4.039e-08 | 4.842e-08 | -0.834 |
| H3K27ac_PGC2 | -6.035e-08 | 3.727e-08 | -1.619 |
| H3K27ac_PGC2.extend.500 | 5.439e-08 | 3.462e-08 | 1.571 |
| H3K4me1_peaks_Trynka | 1.624e-08 | 2.689e-08 | 0.604 |
| H3K4me1_Trynka | 2.740e-08 | 2.279e-08 | 1.202 |
| H3K4me1_Trynka.extend.500 | -1.606e-08 | 1.792e-08 | -0.896 |

**Table S4i (continued). Coefficient of simultaneously modelling blood and brain eQTLs for smoking behavior**

| Category | Coefficient | SE | Z |
| --- | --- | --- | --- |
| H3K4me3_peaks_Trynka | 1.374e-08 | 6.412e-08 | 0.214 |
| H3K4me3_Trynka | 1.363e-09 | 3.270e-08 | 0.042 |
| H3K4me3_Trynka.extend.500 | -8.825e-09 | 1.950e-08 | -0.453 |
| H3K9ac_peaks_Trynka | 4.474e-08 | 6.122e-08 | 0.731 |
| H3K9ac_Trynka | -2.961e-09 | 3.750e-08 | -0.079 |
| H3K9ac_Trynka.extend.500 | -2.604e-08 | 2.457e-08 | -1.060 |
| Intron_UCSC | 5.351e-09 | 1.058e-07 | 0.051 |
| Intron_UCSC.extend.500 | -7.127e-09 | 1.058e-07 | -0.067 |
| PromoterFlanking_Hoffman | -2.640e-08 | 1.050e-07 | -0.252 |
| PromoterFlanking_Hoffman.extend.500 | -3.820e-09 | 5.004e-08 | -0.076 |
| Promoter_UCSC | 3.461e-08 | 1.157e-07 | 0.299 |
| Promoter_UCSC.extend.500 | -4.373e-08 | 9.635e-08 | -0.454 |
| Repressed_Hoffman | -1.539e-08 | 1.688e-08 | -0.911 |
| Repressed_Hoffman.extend.500 | -8.111e-09 | 1.371e-08 | -0.592 |
| SuperEnhancer_Hnisz | 1.970e-07 | 1.395e-07 | 1.412 |
| SuperEnhancer_Hnisz.extend.500 | -2.088e-07 | 1.379e-07 | -1.514 |
| TFBS_ENCODE | 7.247e-08 | 3.751e-08 | 1.932* |
| TFBS_ENCODE.extend.500 | -3.566e-08 | 2.065e-08 | -1.727 |
| Transcribed_Hoffman | -1.563e-08 | 1.508e-08 | -1.037 |
| Transcribed_Hoffman.extend.500 | 4.172e-09 | 9.587e-09 | 0.435 |
| TSS_Hoffman | -1.245e-09 | 1.005e-07 | -0.012 |
| TSS_Hoffman.extend.500 | -2.577e-08 | 6.357e-08 | -0.405 |
| UTR_3_UCSC | -3.607e-08 | 8.075e-08 | -0.447 |
| UTR_3_UCSC.extend.500 | -1.498e-08 | 3.846e-08 | -0.389 |
| UTR_5_UCSC | -8.529e-08 | 9.846e-08 | -0.866 |
| UTR_5_UCSC.extend.500 | -1.265e-09 | 3.194e-08 | -0.040 |

**Table S4i (continued). Coefficient of simultaneously modelling blood and brain eQTLs for smoking behavior**

| Category | Coefficient | SE | Z |
| --- | --- | --- | --- |
| WeakEnhancer_Hoffman | 1.365e-08 | 8.704e-08 | 0.157 |
| WeakEnhancer_Hoffman.extend.500 | -1.173e-08 | 3.775e-08 | -0.311 |
| eQTL_blood_Jansen | 5.438e-08 | 3.692e-08 | 1.473 |
| eQTL_blood_Jansen.extend.100 | -5.643e-08 | 5.325e-08 | -1.060 |
| eQTL_blood_Jansen.extend.500 | 1.009e-09 | 1.921e-08 | 0.053 |
| eQTL_blood_Jansen.extension.GeneCentric | -7.134e-07 | 5.077e-07 | -1.405 |
| eQTL_blood_Jansen.extension.GeneCentric.extension.100 | 6.078e-07 | 5.965e-07 | 1.019 |
| eQTL_blood_Jansen.extension.GeneCentric.extension.500 | 1.110e-07 | 2.160e-07 | 0.514 |
| eQTL_brain_Ramasamy | 5.551e-08 | 4.894e-08 | 1.134 |
| eQTL_brain_Ramasamy.extend.100 | -7.901e-08 | 7.472e-08 | -1.057 |
| eQTL_brain_Ramasamy.extend.500 | 2.180e-08 | 2.827e-08 | 0.771 |
| eQTL_brain_Ramasamy.extension.GeneCentric | 6.372e-08 | 1.592e-07 | 0.400 |
| eQTL_brain_Ramasamy.extension.GeneCentric.extension.100 | 3.880e-07 | 2.903e-07 | 1.337 |
| eQTL_brain_Ramasamy.extension.GeneCentric.extension.500 | -4.479e-07 | 2.287e-07 | -1.959 |

From left to right, columns indicate 1) annotation, 2) the corresponding coefficient, 3) standard error of the coefficient, and 4) the Z-score of the coefficient. One asterisk indicates categories reaching nomial significance (*p*<0.05).

**Table S4j. Coefficient of simultaneously modelling blood and brain eQTLs for schizophrenia**

| Category | Coefficient | SE | Z |
| --- | --- | --- | --- |
| base | -2.458e-08 | 5.471e-08 | -0.449 |
| Coding_UCSC | 3.810e-07 | 3.814e-07 | 0.999 |
| Coding_UCSC.extend.500 | -2.021e-07 | 1.109e-07 | -1.823 |
| Conserved_LindbladToh | 2.068e-06 | 3.387e-07 | 6.105*** |
| Conserved_LindbladToh.extend.500 | 1.002e-07 | 4.901e-08 | 2.045* |
| CTCF_Hoffman | -3.359e-07 | 3.170e-07 | -1.060 |
| CTCF_Hoffman.extend.500 | 1.446e-07 | 1.418e-07 | 1.020 |
| DGF_ENCODE | 3.616e-07 | 1.606e-07 | 2.252* |
| DGF_ENCODE.extend.500 | -5.343e-08 | 7.721e-08 | -0.692 |
| DHS_peaks_Trynka | -4.547e-07 | 2.219e-07 | -2.049 |
| DHS_Trynka | -2.879e-07 | 1.941e-07 | -1.483 |
| DHS_Trynka.extend.500 | 1.234e-07 | 8.065e-08 | 1.530 |
| Enhancer_Andersson | -3.184e-07 | 7.045e-07 | -0.452 |
| Enhancer_Andersson.extend.500 | -3.601e-07 | 2.273e-07 | -1.584 |
| Enhancer_Hoffman | -4.719e-07 | 2.226e-07 | -2.120 |
| Enhancer_Hoffman.extend.500 | 6.489e-08 | 1.333e-07 | 0.487 |
| FetalDHS_Trynka | 3.178e-07 | 2.374e-07 | 1.339 |
| FetalDHS_Trynka.extend.500 | 2.172e-08 | 9.113e-08 | 0.238 |
| H3K27ac_Hnisz | -4.372e-08 | 1.871e-07 | -0.234 |
| H3K27ac_Hnisz.extend.500 | -1.820e-08 | 1.880e-07 | -0.097 |
| H3K27ac_PGC2 | -2.479e-07 | 1.332e-07 | -1.861 |
| H3K27ac_PGC2.extend.500 | 2.665e-07 | 1.242e-07 | 2.145* |
| H3K4me1_peaks_Trynka | 1.837e-07 | 1.187e-07 | 1.548 |
| H3K4me1_Trynka | -1.824e-08 | 9.424e-08 | -0.194 |
| H3K4me1_Trynka.extend.500 | 3.522e-08 | 7.011e-08 | 0.502 |

**Table S4j (continued). Coefficient of simultaneously modelling blood and brain eQTLs for schizophrenia**

| Category | Coefficient | SE | Z |
| --- | --- | --- | --- |
| H3K4me3_peaks_Trynka | 3.463e-07 | 2.630e-07 | 1.317 |
| H3K4me3_Trynka | 1.031e-07 | 1.451e-07 | 0.711 |
| H3K4me3_Trynka.extend.500 | -1.458e-07 | 8.856e-08 | -1.646 |
| H3K9ac_peaks_Trynka | 8.935e-07 | 2.581e-07 | 3.462*** |
| H3K9ac_Trynka | -1.911e-07 | 1.469e-07 | -1.301 |
| H3K9ac_Trynka.extend.500 | 1.374e-07 | 9.806e-08 | 1.401 |
| Intron_UCSC | 1.358e-06 | 4.204e-07 | 3.229*** |
| Intron_UCSC.extend.500 | -1.289e-06 | 4.173e-07 | -3.089 |
| PromoterFlanking_Hoffman | 5.123e-07 | 5.326e-07 | 0.962 |
| PromoterFlanking_Hoffman.extend.500 | -1.671e-07 | 1.942e-07 | -0.860 |
| Promoter_UCSC | 5.760e-07 | 5.495e-07 | 1.048 |
| Promoter_UCSC.extend.500 | -6.002e-07 | 4.813e-07 | -1.247 |
| Repressed_Hoffman | 1.164e-07 | 8.149e-08 | 1.429 |
| Repressed_Hoffman.extend.500 | -5.833e-08 | 6.985e-08 | -0.835 |
| SuperEnhancer_Hnisz | 3.562e-07 | 5.874e-07 | 0.606 |
| SuperEnhancer_Hnisz.extend.500 | -3.806e-07 | 5.743e-07 | -0.663 |
| TFBS_ENCODE | 2.714e-07 | 1.354e-07 | 2.004* |
| TFBS_ENCODE.extend.500 | -1.438e-07 | 7.845e-08 | -1.833 |
| Transcribed_Hoffman | -1.396e-08 | 6.290e-08 | -0.222 |
| Transcribed_Hoffman.extend.500 | 5.854e-08 | 4.714e-08 | 1.242 |
| TSS_Hoffman | -2.378e-07 | 4.203e-07 | -0.566 |
| TSS_Hoffman.extend.500 | 2.388e-07 | 2.870e-07 | 0.832 |
| UTR_3_UCSC | 7.551e-07 | 5.642e-07 | 1.338 |
| UTR_3_UCSC.extend.500 | -1.612e-07 | 2.338e-07 | -0.689 |
| UTR_5_UCSC | -2.397e-07 | 4.416e-07 | -0.543 |
| UTR_5_UCSC.extend.500 | 1.573e-07 | 1.787e-07 | 0.880 |

**Table S4j (continued). Coefficient of simultaneously modelling blood and brain eQTLs for schizophrenia**

| Category | Coefficient | SE | Z |
| --- | --- | --- | --- |
| WeakEnhancer_Hoffman | -3.181e-08 | 3.320e-07 | -0.096 |
| WeakEnhancer_Hoffman.extend.500 | 1.473e-07 | 1.670e-07 | 0.882 |
| eQTL_blood_Jansen | 1.630e-07 | 1.451e-07 | 1.124 |
| eQTL_blood_Jansen.extend.100 | 6.768e-08 | 2.138e-07 | 0.317 |
| eQTL_blood_Jansen.extend.500 | -1.990e-07 | 8.203e-08 | -2.426 |
| eQTL_blood_Jansen.extension.GeneCentric | 2.654e-06 | 2.093e-06 | 1.268 |
| eQTL_blood_Jansen.extension.GeneCentric.extension.100 | -4.185e-06 | 2.417e-06 | -1.732 |
| eQTL_blood_Jansen.extension.GeneCentric.extension.500 | 1.538e-06 | 9.943e-07 | 1.547 |
| eQTL_brain_Ramasamy | 6.960e-07 | 1.992e-07 | 3.495*** |
| eQTL_brain_Ramasamy.extend.100 | -7.505e-07 | 2.980e-07 | -2.518 |
| eQTL_brain_Ramasamy.extend.500 | 1.350e-07 | 1.121e-07 | 1.204 |
| eQTL_brain_Ramasamy.extension.GeneCentric | -1.628e-07 | 1.550e-06 | -0.105 |
| eQTL_brain_Ramasamy.extension.GeneCentric.extension.100 | 5.199e-07 | 2.016e-06 | 0.258 |
| eQTL_brain_Ramasamy.extension.GeneCentric.extension.500 | -3.680e-07 | 9.096e-07 | -0.405 |

From left to right, columns indicate 1) annotation, 2) the corresponding coefficient, 3) standard error of the coefficient, and 4) the Z-score of the coefficient. One asterisk indicates categories reaching nomial significance (*p*<0.05). Three asterisks indicate categories passing Bonferroni correction for multiple testing.

**Table S4k. Coefficient of simultaneously modelling blood and brain eQTLs for ulcerative colitis**

| Category | Coefficient | SE | Z |
| --- | --- | --- | --- |
| base | -6.358e-08 | 3.798e-08 | -1.674 |
| Coding_UCSC | 3.285e-07 | 2.995e-07 | 1.097 |
| Coding_UCSC.extend.500 | 3.878e-08 | 9.105e-08 | 0.426 |
| Conserved_LindbladToh | 3.100e-07 | 1.817e-07 | 1.706* |
| Conserved_LindbladToh.extend.500 | 2.392e-08 | 2.883e-08 | 0.830 |
| CTCF_Hoffman | -2.923e-07 | 2.925e-07 | -0.999 |
| CTCF_Hoffman.extend.500 | -4.463e-08 | 1.407e-07 | -0.317 |
| DGF_ENCODE | 2.000e-07 | 1.456e-07 | 1.374 |
| DGF_ENCODE.extend.500 | 7.931e-08 | 5.052e-08 | 1.570 |
| DHS_peaks_Trynka | 2.237e-07 | 2.029e-07 | 1.103 |
| DHS_Trynka | -2.917e-07 | 2.011e-07 | -1.451 |
| DHS_Trynka.extend.500 | 3.266e-08 | 5.980e-08 | 0.546 |
| Enhancer_Andersson | 1.582e-06 | 6.735e-07 | 2.349* |
| Enhancer_Andersson.extend.500 | 2.385e-07 | 2.013e-07 | 1.185 |
| Enhancer_Hoffman | -1.401e-07 | 2.181e-07 | -0.642 |
| Enhancer_Hoffman.extend.500 | 6.298e-08 | 1.648e-07 | 0.382 |
| FetalDHS_Trynka | -1.085e-07 | 1.991e-07 | -0.545 |
| FetalDHS_Trynka.extend.500 | -9.821e-09 | 6.840e-08 | -0.144 |
| H3K27ac_Hnisz | 2.382e-09 | 1.520e-07 | 0.016 |
| H3K27ac_Hnisz.extend.500 | 5.695e-08 | 1.438e-07 | 0.396 |
| H3K27ac_PGC2 | -2.066e-08 | 1.254e-07 | -0.165 |
| H3K27ac_PGC2.extend.500 | 1.746e-08 | 1.124e-07 | 0.155 |
| H3K4me1_peaks_Trynka | 2.123e-08 | 1.087e-07 | 0.195 |
| H3K4me1_Trynka | -3.584e-08 | 9.410e-08 | -0.381 |
| H3K4me1_Trynka.extend.500 | -4.479e-08 | 5.943e-08 | -0.754 |

**Table S4k (continued). Coefficient of simultaneously modelling blood and brain eQTLs for ulcerative colitis**

| Category | Coefficient | SE | Z |
| --- | --- | --- | --- |
| H3K4me3_peaks_Trynka | -7.374e-09 | 2.711e-07 | -0.027 |
| H3K4me3_Trynka | 1.410e-07 | 1.470e-07 | 0.959 |
| H3K4me3_Trynka.extend.500 | -1.835e-08 | 8.336e-08 | -0.220 |
| H3K9ac_peaks_Trynka | 2.104e-08 | 2.487e-07 | 0.085 |
| H3K9ac_Trynka | 2.401e-09 | 1.617e-07 | 0.015 |
| H3K9ac_Trynka.extend.500 | -1.028e-08 | 9.347e-08 | -0.110 |
| Intron_UCSC | 1.381e-08 | 4.115e-07 | 0.034 |
| Intron_UCSC.extend.500 | -3.647e-08 | 4.109e-07 | -0.089 |
| PromoterFlanking_Hoffman | -1.127e-09 | 4.482e-07 | -0.003 |
| PromoterFlanking_Hoffman.extend.500 | -1.646e-07 | 1.714e-07 | -0.960 |
| Promoter_UCSC | -4.621e-07 | 5.034e-07 | -0.918 |
| Promoter_UCSC.extend.500 | 3.068e-07 | 4.261e-07 | 0.720 |
| Repressed_Hoffman | 3.439e-08 | 4.757e-08 | 0.723 |
| Repressed_Hoffman.extend.500 | -1.872e-10 | 4.374e-08 | -0.004 |
| SuperEnhancer_Hnisz | -7.783e-07 | 6.156e-07 | -1.264 |
| SuperEnhancer_Hnisz.extend.500 | 7.850e-07 | 6.133e-07 | 1.280 |
| TFBS_ENCODE | 4.560e-07 | 1.812e-07 | 2.517* |
| TFBS_ENCODE.extend.500 | -1.456e-07 | 8.011e-08 | -1.817 |
| Transcribed_Hoffman | 2.497e-08 | 5.866e-08 | 0.426 |
| Transcribed_Hoffman.extend.500 | 1.204e-08 | 4.248e-08 | 0.283 |
| TSS_Hoffman | -2.474e-07 | 3.595e-07 | -0.688 |
| TSS_Hoffman.extend.500 | 1.393e-07 | 2.699e-07 | 0.516 |
| UTR_3_UCSC | -5.811e-07 | 3.319e-07 | -1.751 |
| UTR_3_UCSC.extend.500 | 2.794e-07 | 1.814e-07 | 1.541 |
| UTR_5_UCSC | 3.102e-07 | 4.643e-07 | 0.668 |
| UTR_5_UCSC.extend.500 | 5.297e-08 | 1.553e-07 | 0.341 |

**Table S4k (continued). Coefficient of simultaneously modelling blood and brain eQTLs for ulcerative colitis**

| Category | Coefficient | SE | Z |
| --- | --- | --- | --- |
| WeakEnhancer_Hoffman | -2.655e-07 | 2.951e-07 | -0.900 |
| WeakEnhancer_Hoffman.extend.500 | 4.766e-08 | 1.624e-07 | 0.294 |
| eQTL_blood_Jansen | 1.368e-07 | 1.469e-07 | 0.931 |
| eQTL_blood_Jansen.extend.100 | -6.713e-08 | 2.135e-07 | -0.314 |
| eQTL_blood_Jansen.extend.500 | -4.312e-08 | 7.772e-08 | -0.555 |
| eQTL_blood_Jansen.extension.GeneCentric | -5.208e-07 | 2.108e-06 | -0.247 |
| eQTL_blood_Jansen.extension.GeneCentric.extension.100 | -7.144e-07 | 2.455e-06 | -0.291 |
| eQTL_blood_Jansen.extension.GeneCentric.extension.500 | 1.210e-06 | 9.041e-07 | 1.339 |
| eQTL_brain_Ramasamy | 1.133e-07 | 1.862e-07 | 0.608 |
| eQTL_brain_Ramasamy.extend.100 | -3.064e-08 | 2.683e-07 | -0.114 |
| eQTL_brain_Ramasamy.extend.500 | -6.052e-08 | 9.086e-08 | -0.666 |
| eQTL_brain_Ramasamy.extension.GeneCentric | 1.026e-07 | 1.567e-07 | 0.655 |
| eQTL_brain_Ramasamy.extension.GeneCentric.extension.100 | 1.149e-06 | 1.060e-06 | 1.084 |
| eQTL_brain_Ramasamy.extension.GeneCentric.extension.500 | -1.248e-06 | 1.101e-06 | -1.133 |

From left to right, columns indicate 1) annotation, 2) the corresponding coefficient, 3) standard error of the coefficient, and 4) the Z-score of the coefficient. One asterisk indicates categories reaching nomial significance (*p*<0.05).

**Table S5. Coefficient Z-score of simultaneously modelling blood and brain eQTLs, meta-analyzed over all traits**

| Category | Z score |
| --- | --- |
| base | 0.588(0.278) |
| Coding_UCSC | 1.058(0.145) |
| Coding_UCSC.extend.500 | -1.383(0.917) |
| Conserved_LindbladToh | 12.739(0.000)*** |
| Conserved_LindbladToh.extend.500 | 0.997(0.159) |
| CTCF_Hoffman | -1.656(0.951) |
| CTCF_Hoffman.extend.500 | 0.011(0.496) |
| DGF_ENCODE | 2.331(0.010)* |
| DGF_ENCODE.extend.500 | -1.065(0.857) |
| DHS_peaks_Trynka | 0.233(0.408) |
| DHS_Trynka | -3.811(1.000) |
| DHS_Trynka.extend.500 | 1.911(0.028)* |
| Enhancer_Andersson | 0.013(0.495) |
| Enhancer_Andersson.extend.500 | -1.007(0.843) |
| Enhancer_Hoffman | 0.537(0.296) |
| Enhancer_Hoffman.extend.500 | -0.644(0.740) |
| FetalDHS_Trynka | 1.146(0.126) |
| FetalDHS_Trynka.extend.500 | 0.529(0.299) |
| H3K27ac_Hnisz | 0.815(0.208) |
| H3K27ac_Hnisz.extend.500 | -0.953(0.830) |
| H3K27ac_PGC2 | -0.976(0.836) |
| H3K27ac_PGC2.extend.500 | 0.846(0.199) |
| H3K4me1_peaks_Trynka | 1.367(0.086) |
| H3K4me1_Trynka | 2.049(0.020)* |
| H3K4me1_Trynka.extend.500 | 0.008(0.497) |
| H3K4me3_peaks_Trynka | 0.548(0.292) |
| H3K4me3_Trynka | -0.035(0.514) |
| H3K4me3_Trynka.extend.500 | -0.868(0.807) |
| H3K9ac_peaks_Trynka | 4.025(0.000)*** |
| H3K9ac_Trynka | -0.221(0.587) |
| H3K9ac_Trynka.extend.500 | 1.117(0.132) |
| Intron_UCSC | 1.051(0.147) |
| Intron_UCSC.extend.500 | -0.981(0.837) |
| PromoterFlanking_Hoffman | -2.497(0.994) |
| PromoterFlanking_Hoffman.extend.500 | 0.384(0.351) |
| Promoter_UCSC | 1.136(0.128) |
| Promoter_UCSC.extend.500 | -1.488(0.932) |
| Repressed_Hoffman | 0.725(0.234) |
| Repressed_Hoffman.extend.500 | -0.161(0.564) |
| SuperEnhancer_Hnisz | -0.862(0.806) |
| SuperEnhancer_Hnisz.extend.500 | 0.779(0.218) |

**Table S5. Coefficient Z-score of simultaneously modelling blood and brain eQTLs, meta-analyzed over all traits**

| Category | Z score |
| --- | --- |
| TFBS_ENCODE | 3.558(0.000)*** |
| TFBS_ENCODE.extend.500 | -1.876(0.970) |
| Transcribed_Hoffman | 1.778(0.038)* |
| Transcribed_Hoffman.extend.500 | -0.759(0.776) |
| TSS_Hoffman | 0.396(0.346) |
| TSS_Hoffman.extend.500 | 1.971(0.024)* |
| UTR_3_UCSC | 1.689(0.046)* |
| UTR_3_UCSC.extend.500 | -1.994(0.977) |
| UTR_5_UCSC | 1.259(0.104) |
| UTR_5_UCSC.extend.500 | -2.728(0.997) |
| WeakEnhancer_Hoffman | -0.477(0.683) |
| WeakEnhancer_Hoffman.extend.500 | 0.834(0.202) |
| eQTL_blood_Jansen | 4.907(0.000)*** |
| eQTL_blood_Jansen.extend.100 | -3.308(1.000) |
| eQTL_blood_Jansen.extend.500 | 1.067(0.143) |
| eQTL_blood_Jansen.extension.GeneCentric | 2.063(0.020)* |
| eQTL_blood_Jansen.extension.GeneCentric.extension.100 | -2.375(0.991) |
| eQTL_blood_Jansen.extension.GeneCentric.extension.500 | 1.853(0.032)* |
| eQTL_brain_Ramasamy | 4.905(0.000)*** |
| eQTL_brain_Ramasamy.extend.100 | -3.274(0.999) |
| eQTL_brain_Ramasamy.extend.500 | 0.716(0.237) |
| eQTL_brain_Ramasamy.extension.GeneCentric | -0.176(0.570) |
| eQTL_brain_Ramasamy.extension.GeneCentric.extension.100 | 0.819(0.206) |
| eQTL_brain_Ramasamy.extension.GeneCentric.extension.500 | -0.461(0.678) |

Left column indicates the SLDSC annotation. Right column indicates the meta-analyzed Z-score. Values between brackets show the corresponding *p*-value. One asterisk indicates categories reaching nomial significance (*p*<0.05). Three asterisks indicate categories passing Bonferroni correction for multiple testing.

**Table S6. Results of single-tissue GTEx annotations**

|  |  |  | Rheumatoid arthritis | | | Schizophrenia | | |
| --- | --- | --- | --- | --- | --- | --- | --- | --- |
| Tissue | Sample size | Prop.SNPs | Coefficient | SE | Z | Coefficient | SE | Z |
| GTEx union | NA | 0.354 | 1.307e-07 | 4.552e-08 | 2.871* | 6.044e-07 | 1.231e-07 | 4.911*** |
| Brain union | NA | 0.128 | 1.250e-07 | 4.695e-08 | 2.662* | 8.078e-08 | 1.302e-07 | 0.621 |
| Whole Blood | 338 | 0.087 | 1.922e-08 | 8.538e-09 | 2.251* | 4.739e-08 | 2.889e-08 | 1.640 |
| Lung | 278 | 0.090 | 1.476e-08 | 8.247e-09 | 1.790* | -1.300e-08 | 2.745e-08 | -0.474 |
| Artery Coronary | 118 | 0.029 | 1.953e-08 | 1.200e-08 | 1.628 | -8.472e-08 | 3.889e-08 | -2.178 |
| Brain Cerebellum | 103 | 0.048 | 1.772e-08 | 1.195e-08 | 1.482 | 1.484e-08 | 3.608e-08 | 0.411 |
| Thyroid | 278 | 0.122 | 1.052e-08 | 7.150e-09 | 1.472 | 3.817e-08 | 2.631e-08 | 1.451 |
| Pancreas | 149 | 0.051 | 1.379e-08 | 9.846e-09 | 1.401 | 2.598e-09 | 3.587e-08 | 0.072 |
| Spleen | 89 | 0.029 | 1.836e-08 | 1.334e-08 | 1.377 | -5.793e-09 | 5.149e-08 | -0.113 |
| Artery Tibial | 285 | 0.103 | 1.045e-08 | 7.792e-09 | 1.341 | -2.480e-08 | 2.749e-08 | -0.902 |
| Esophagus Mucosa | 241 | 0.094 | 8.791e-09 | 6.830e-09 | 1.287 | 3.275e-08 | 2.719e-08 | 1.204 |
| Heart Atrial Appendage | 159 | 0.050 | 1.113e-08 | 9.807e-09 | 1.135 | -1.907e-08 | 3.405e-08 | -0.560 |
| Heart Left Ventricle | 190 | 0.058 | 9.171e-09 | 9.389e-09 | 0.977 | -1.802e-08 | 3.389e-08 | -0.532 |
| Muscle Skeletal | 361 | 0.093 | 7.778e-09 | 8.473e-09 | 0.918 | 7.517e-09 | 2.597e-08 | 0.289 |
| Brain Cerebellar Hemisphere | 89 | 0.036 | 1.094e-08 | 1.239e-08 | 0.883 | -3.288e-08 | 3.986e-08 | -0.825 |
| Artery Aorta | 197 | 0.078 | 7.965e-09 | 9.072e-09 | 0.878 | -9.914e-09 | 3.053e-08 | -0.325 |
| Testis | 157 | 0.105 | 5.938e-09 | 7.045e-09 | 0.843 | 2.208e-08 | 2.788e-08 | 0.792 |
| Stomach | 170 | 0.044 | 8.362e-09 | 9.996e-09 | 0.837 | 2.570e-08 | 3.663e-08 | 0.702 |
| Skin Not Sun Exposed Suprapubic | 196 | 0.065 | 7.394e-09 | 8.924e-09 | 0.829 | 4.881e-09 | 3.098e-08 | 0.158 |
| Skin Sun Exposed Lower leg | 302 | 0.107 | 5.718e-09 | 7.649e-09 | 0.748 | 6.701e-09 | 2.714e-08 | 0.247 |
| Cells EBV-transformed lymphocytes | 114 | 0.034 | 8.911e-09 | 1.246e-08 | 0.715 | 8.135e-09 | 4.440e-08 | 0.183 |
| Colon Transverse | 169 | 0.055 | 5.898e-09 | 8.422e-09 | 0.700 | 2.750e-08 | 3.794e-08 | 0.725 |
| Esophagus Muscularis | 218 | 0.088 | 5.346e-09 | 8.104e-09 | 0.660 | -4.616e-09 | 2.872e-08 | -0.161 |
| Liver | 97 | 0.019 | 1.293e-08 | 2.001e-08 | 0.646 | -6.050e-08 | 5.247e-08 | -1.153 |
| Brain Putamen basal ganglia | 82 | 0.018 | 9.760e-09 | 1.611e-08 | 0.606 | -7.487e-08 | 4.906e-08 | -1.526 |

**Table S6 (continued). Results of single-tissue GTEx annotations**

|  |  |  | Rheumatoid arthritis | | | Schizophrenia | | |
| --- | --- | --- | --- | --- | --- | --- | --- | --- |
| Tissue | Sample size | Prop.SNPs | Coefficient | SE | Z | Coefficient | SE | Z |
| Brain Nucleus accumbens basal ganglia | 93 | 0.025 | 8.911e-09 | 1.520e-08 | 0.586 | -5.548e-08 | 4.317e-08 | -1.285 |
| Nerve Tibial | 256 | 0.119 | 3.915e-09 | 7.147e-09 | 0.548 | 7.869e-09 | 2.706e-08 | 0.291 |
| Pituitary | 87 | 0.026 | 4.112e-09 | 1.109e-08 | 0.371 | -3.547e-08 | 4.931e-08 | -0.719 |
| Brain Caudate basal ganglia | 100 | 0.030 | 4.552e-09 | 1.241e-08 | 0.367 | -3.955e-08 | 3.949e-08 | -1.002 |
| Vagina | 79 | 0.012 | 6.777e-09 | 2.024e-08 | 0.335 | -1.594e-07 | 6.425e-08 | -2.480 |
| Esophagus Gastroesophageal Junction | 127 | 0.036 | 3.589e-09 | 1.079e-08 | 0.333 | -4.085e-08 | 4.156e-08 | -0.983 |
| Adipose Subcutaneous | 298 | 0.105 | 2.448e-09 | 8.143e-09 | 0.301 | 2.547e-08 | 2.759e-08 | 0.923 |
| Uterus | 70 | 0.010 | 3.823e-09 | 2.482e-08 | 0.154 | -1.409e-07 | 7.135e-08 | -1.974 |
| Adipose Visceral Omentum | 185 | 0.055 | 1.199e-09 | 7.833e-09 | 0.153 | -2.959e-08 | 3.190e-08 | -0.927 |
| Adrenal Gland | 126 | 0.039 | 1.156e-09 | 1.016e-08 | 0.114 | -2.368e-08 | 4.629e-08 | -0.512 |
| Breast Mammary Tissue | 183 | 0.052 | 2.136e-10 | 8.457e-09 | 0.025 | -1.370e-08 | 3.097e-08 | -0.442 |
| Ovary | 85 | 0.017 | 3.401e-11 | 1.588e-08 | 0.002 | -1.782e-07 | 5.135e-08 | -3.470 |
| Brain Cortex | 96 | 0.030 | -1.138e-09 | 1.165e-08 | -0.098 | -2.579e-08 | 4.042e-08 | -0.638 |
| Small Intestine Terminal Ileum | 77 | 0.015 | -1.764e-09 | 1.472e-08 | -0.120 | -6.793e-08 | 5.690e-08 | -1.194 |
| Colon Sigmoid | 124 | 0.036 | -2.451e-09 | 9.843e-09 | -0.249 | -5.223e-08 | 3.779e-08 | -1.382 |
| Brain Hippocampus | 81 | 0.014 | -5.119e-09 | 1.563e-08 | -0.328 | -8.052e-08 | 6.321e-08 | -1.274 |
| Brain Frontal Cortex BA9 | 92 | 0.025 | -4.491e-09 | 1.334e-08 | -0.337 | -9.978e-09 | 4.528e-08 | -0.220 |
| Brain Anterior cingulate cortex BA24 | 72 | 0.015 | -6.200e-09 | 1.469e-08 | -0.422 | -1.123e-07 | 4.887e-08 | -2.297 |
| Cells Transformed fibroblasts | 272 | 0.111 | -3.108e-09 | 6.927e-09 | -0.449 | 1.023e-08 | 2.694e-08 | 0.380 |
| Prostate | 87 | 0.017 | -8.375e-09 | 1.321e-08 | -0.634 | -6.180e-08 | 6.526e-08 | -0.947 |
| Brain Hypothalamus | 81 | 0.015 | -1.302e-08 | 1.424e-08 | -0.914 | -1.385e-07 | 5.294e-08 | -2.617 |

From left to right, columns show 1) tissue from which eQTLs were obtained, 2) the discovery sample size, 3) the proportion of SNPs included in each annotation, 4) the regression coefficient for rheumatoid arthritis, 5) the standard error of the coefficient, 6) the corresponding Z-score, and the last three columns repeated for schizophrenia. One asterisk indicates categories reaching nomial significance (*p*<0.05). Three asterisks indicate categories passing Bonferroni correction for multiple testing.

**Table S7. Results of intersection between eQTLs and cell-type-specific differentially epigenetically modified regions for schizophrenia**

| Tissue type | Tissue | Histone | Prop.SNPs | Coefficient | SE | Z |
| --- | --- | --- | --- | --- | --- | --- |
| CNS | Angular_gyrus | H3K27ac | 0.011 | 2.344e-07 | 2.732e-07 | 0.858 |
| CNS | Anterior_caudate | H3K27ac | 0.009 | 1.281e-07 | 2.639e-07 | 0.485 |
| CNS | Cingulate_gyrus | H3K27ac | 0.011 | 1.323e-07 | 2.393e-07 | 0.553 |
| CNS | Hippocampus_middle | H3K27ac | 0.010 | 3.491e-08 | 2.558e-07 | 0.136 |
| CNS | Inferior_temporal_lobe | H3K27ac | 0.011 | 1.367e-07 | 2.553e-07 | 0.536 |
| CNS | Mid_frontal_lobe | H3K27ac | 0.009 | 5.432e-07 | 3.650e-07 | 1.488 |
| CNS | Neurosphere | H3K27ac | 0.013 | 4.570e-07 | 2.284e-07 | 2.001* |
| CNS | Substantia_nigra | H3K27ac | 0.012 | 1.941e-07 | 2.077e-07 | 0.935 |
| CNS | Angular_gyrus | H3K4me1 | 0.007 | 1.261e-06 | 4.563e-07 | 2.764* |
| CNS | Anterior_caudate | H3K4me1 | 0.019 | 6.343e-07 | 2.184e-07 | 2.904* |
| CNS | Cingulate_gyrus | H3K4me1 | 0.020 | 5.169e-07 | 2.142e-07 | 2.413* |
| CNS | Fetal_brain | H3K4me1 | 0.024 | 7.044e-08 | 1.742e-07 | 0.404 |
| CNS | Fetal_thymus | H3K4me1 | 0.019 | 2.108e-07 | 1.912e-07 | 1.103 |
| CNS | Hippocampus_middle | H3K4me1 | 0.026 | 4.101e-07 | 1.752e-07 | 2.341* |
| CNS | Inferior_temporal_lobe | H3K4me1 | 0.024 | 4.393e-07 | 1.921e-07 | 2.287* |
| CNS | Mid_frontal_lobe | H3K4me1 | 0.002 | 2.534e-06 | 1.043e-06 | 2.431* |
| CNS | Substantia_nigra | H3K4me1 | 0.018 | 5.347e-07 | 2.156e-07 | 2.480* |
| CNS | Angular_gyrus | H3K4me3 | 0.007 | -1.172e-06 | 7.749e-07 | -1.513 |
| CNS | Anterior_caudate | H3K4me3 | 0.012 | -4.792e-07 | 5.315e-07 | -0.902 |
| CNS | Cingulate_gyrus | H3K4me3 | 0.011 | -4.561e-07 | 5.802e-07 | -0.786 |
| CNS | Fetal_brain | H3K4me3 | 0.003 | -2.239e-06 | 1.888e-06 | -1.185 |
| CNS | Fetal_thymus | H3K4me3 | 0.007 | -5.405e-08 | 6.514e-07 | -0.083 |
| CNS | Germinal_matrix | H3K4me3 | 0.008 | -1.147e-06 | 8.324e-07 | -1.378 |
| CNS | Hippocampus_middle | H3K4me3 | 0.012 | -4.655e-07 | 5.064e-07 | -0.919 |
| CNS | Inferior_temporal_lobe | H3K4me3 | 0.011 | -7.047e-07 | 5.882e-07 | -1.198 |
| CNS | Mid_frontal_lobe | H3K4me3 | 0.011 | -7.338e-07 | 5.964e-07 | -1.230 |

**Table S7 (continued). Results of intersection between eQTLs and cell-type-specific differentially epigenetically modified regions for schizophrenia**

| Tissue type | Tissue | Histone | Prop.SNPs | Coefficient | SE | Z |
| --- | --- | --- | --- | --- | --- | --- |
| CNS | Substantia_nigra | H3K4me3 | 0.009 | -6.337e-07 | 5.961e-07 | -1.063 |
| CNS | Angular_gyrus | H3K9ac | 0.007 | -6.481e-07 | 6.499e-07 | -0.997 |
| CNS | Anterior_caudate | H3K9ac | 0.009 | -1.995e-07 | 5.372e-07 | -0.371 |
| CNS | Cingulate_gyrus | H3K9ac | 0.012 | 1.023e-07 | 4.046e-07 | 0.253 |
| CNS | Fetal_brain | H3K9ac | 0.009 | 3.121e-07 | 5.193e-07 | 0.601 |
| CNS | Hippocampus_middle | H3K9ac | 0.012 | 2.088e-07 | 4.100e-07 | 0.509 |
| CNS | Inferior_temporal_lobe | H3K9ac | 0.011 | 1.776e-07 | 4.562e-07 | 0.389 |
| CNS | Mid_frontal_lobe | H3K9ac | 0.010 | 1.223e-09 | 4.941e-07 | 0.002 |
| CNS | Substantia_nigra | H3K9ac | 0.011 | 2.205e-07 | 4.210e-07 | 0.524 |
| Immune | CD14 | H3K27ac | 0.015 | 5.035e-08 | 1.645e-07 | 0.306 |
| Immune | CD19 | H3K27ac | 0.017 | -2.751e-07 | 1.824e-07 | -1.509 |
| Immune | CD20 | H3K27ac | 0.016 | -2.921e-07 | 2.067e-07 | -1.414 |
| Immune | CD25-_CD45RA+_naive | H3K27ac | 0.016 | -4.230e-08 | 2.894e-07 | -0.146 |
| Immune | CD25-_IL17-_Th_stim_MACS | H3K27ac | 0.013 | -7.861e-08 | 2.561e-07 | -0.307 |
| Immune | CD25-_IL17+_Th17_stim | H3K27ac | 0.015 | 3.077e-07 | 2.452e-07 | 1.255 |
| Immune | CD25+_CD127-_Treg | H3K27ac | 0.018 | 2.170e-07 | 2.353e-07 | 0.922 |
| Immune | CD25int_CD127+_Tmem | H3K27ac | 0.017 | 2.858e-07 | 2.503e-07 | 1.142 |
| Immune | CD3_primary | H3K27ac | 0.016 | -4.610e-08 | 1.955e-07 | -0.236 |
| Immune | Mobilized_CD34 | H3K27ac | 0.017 | 1.049e-07 | 2.293e-07 | 0.458 |
| Immune | Th0 | H3K27ac | 0.015 | 2.953e-07 | 2.097e-07 | 1.408 |
| Immune | Th1 | H3K27ac | 0.015 | 2.396e-08 | 2.022e-07 | 0.118 |
| Immune | Th2 | H3K27ac | 0.014 | 1.230e-07 | 2.143e-07 | 0.574 |
| Immune | CD14_primary | H3K4me1 | 0.022 | 1.046e-07 | 1.586e-07 | 0.659 |
| Immune | CD15_primary | H3K4me1 | 0.021 | 3.195e-09 | 1.604e-07 | 0.020 |
| Immune | CD19_primary_BI | H3K4me1 | 0.024 | -4.770e-08 | 1.633e-07 | -0.292 |
| Immune | CD19_primary_UW | H3K4me1 | 0.023 | 4.253e-08 | 1.709e-07 | 0.249 |

**Table S7 (continued). Results of intersection between eQTLs and cell-type-specific differentially epigenetically modified regions for schizophrenia**

| Tissue type | Tissue | Histone | Prop.SNPs | Coefficient | SE | Z |
| --- | --- | --- | --- | --- | --- | --- |
| Immune | CD3_primary_BI | H3K4me1 | 0.016 | 2.689e-07 | 2.350e-07 | 1.144 |
| Immune | CD3_primary_UW | H3K4me1 | 0.021 | 3.301e-07 | 2.128e-07 | 1.552 |
| Immune | CD34_primary | H3K4me1 | 0.018 | 6.080e-09 | 2.196e-07 | 0.028 |
| Immune | CD4_memory_primary | H3K4me1 | 0.024 | 2.797e-07 | 1.724e-07 | 1.622 |
| Immune | CD4_naive_primary | H3K4me1 | 0.019 | 2.909e-07 | 2.245e-07 | 1.296 |
| Immune | CD4+_CD25-_CD45R0+_memory_primary | H3K4me1 | 0.020 | 3.119e-07 | 1.983e-07 | 1.573 |
| Immune | CD4+_CD25-_CD45RA+_naive_primary | H3K4me1 | 0.022 | 2.951e-07 | 2.057e-07 | 1.435 |
| Immune | CD4+_CD25-_IL17-_PMA_Ionomycin_stim_MACS_Th_sprimary | H3K4me1 | 0.030 | 2.275e-07 | 1.616e-07 | 1.408 |
| Immune | CD4+_CD25-_IL17+_PMA_Ionomycin_stim_Th17_primary | H3K4me1 | 0.023 | 2.274e-07 | 1.861e-07 | 1.222 |
| Immune | CD4+_CD25-_Th_primary | H3K4me1 | 0.026 | 2.801e-07 | 1.867e-07 | 1.500 |
| Immune | CD4+_CD25+_CD127-_Treg_primary | H3K4me1 | 0.017 | 4.495e-07 | 2.422e-07 | 1.856* |
| Immune | CD4+_CD25int_CD127+_Tmem_primary | H3K4me1 | 0.009 | 3.651e-07 | 3.653e-07 | 1.000 |
| Immune | CD56_primary | H3K4me1 | 0.020 | 2.010e-07 | 2.045e-07 | 0.983 |
| Immune | CD8_memory_primary | H3K4me1 | 0.019 | 3.327e-07 | 2.213e-07 | 1.504 |
| Immune | CD8_naive_primary_BI | H3K4me1 | 0.019 | 2.724e-07 | 2.382e-07 | 1.144 |
| Immune | CD8_naive_primary_UCSF_minUBC | H3K4me1 | 0.015 | 2.186e-07 | 2.710e-07 | 0.806 |
| Immune | Mobilized_CD34_primary | H3K4me1 | 0.036 | -7.269e-09 | 1.369e-07 | -0.053 |
| Immune | Peripheralblood_mononuclear_primary | H3K4me1 | 0.006 | 5.471e-07 | 9.181e-07 | 0.596 |
| Immune | Spleen | H3K4me1 | 0.024 | 3.651e-07 | 1.891e-07 | 1.930* |
| Immune | Thymus | H3K4me1 | 0.005 | 1.571e-07 | 4.684e-07 | 0.335 |
| Immune | CD14_primary | H3K4me3 | 0.006 | 9.920e-09 | 6.787e-07 | 0.015 |
| Immune | CD15_primary | H3K4me3 | 0.010 | 5.752e-09 | 3.563e-07 | 0.016 |
| Immune | CD19_primary_BI | H3K4me3 | 0.009 | -6.535e-07 | 4.942e-07 | -1.322 |
| Immune | CD19_primary_UW | H3K4me3 | 0.007 | -5.152e-07 | 6.305e-07 | -0.817 |
| Immune | CD3_primary_BI | H3K4me3 | 0.010 | -2.251e-07 | 5.004e-07 | -0.450 |
| Immune | CD3_primary_UW | H3K4me3 | 0.008 | -7.069e-07 | 8.058e-07 | -0.877 |

**Table S7 (continued). Results of intersection between eQTLs and cell-type-specific differentially epigenetically modified regions for schizophrenia**

| Tissue type | Tissue | Histone | Prop.SNPs | Coefficient | SE | Z |
| --- | --- | --- | --- | --- | --- | --- |
| Immune | CD34_primary | H3K4me3 | 0.008 | -2.993e-07 | 5.704e-07 | -0.525 |
| Immune | CD4_memory_primary | H3K4me3 | 0.008 | 1.982e-08 | 6.127e-07 | 0.032 |
| Immune | CD4_naive_primary | H3K4me3 | 0.009 | -1.675e-07 | 6.521e-07 | -0.257 |
| Immune | CD4+_CD25-_CD45R0+_memory_primary | H3K4me3 | 0.006 | -7.095e-07 | 7.808e-07 | -0.909 |
| Immune | CD4+_CD25-_CD45RA+_naive_primary | H3K4me3 | 0.009 | -5.146e-08 | 7.467e-07 | -0.069 |
| Immune | CD4+_CD25-_IL17-_PMA_Ionomycin_stim_MACS_Th_sprimary | H3K4me3 | 0.012 | -2.073e-07 | 4.686e-07 | -0.442 |
| Immune | CD4+_CD25-_IL17+_PMA_Ionomycin_stim_Th17_primary | H3K4me3 | 0.009 | -1.618e-07 | 5.982e-07 | -0.270 |
| Immune | CD4+_CD25-_Th_primary | H3K4me3 | 0.009 | -3.131e-07 | 7.479e-07 | -0.419 |
| Immune | CD4+_CD25+_CD127-_Treg_primary | H3K4me3 | 0.010 | 9.344e-08 | 5.589e-07 | 0.167 |
| Immune | CD4+_CD25int_CD127+_Tmem_primary | H3K4me3 | 0.008 | -2.694e-07 | 8.094e-07 | -0.333 |
| Immune | CD4_primary | H3K4me3 | 0.009 | -3.233e-07 | 6.224e-07 | -0.519 |
| Immune | CD56_primary | H3K4me3 | 0.006 | -4.227e-07 | 6.246e-07 | -0.677 |
| Immune | CD8_memory_primary | H3K4me3 | 0.008 | -5.967e-08 | 7.176e-07 | -0.083 |
| Immune | CD8_naive_primary_BI | H3K4me3 | 0.008 | -5.616e-07 | 8.684e-07 | -0.647 |
| Immune | CD8_naive_primary_UCSF_minUBC | H3K4me3 | 0.005 | -1.253e-06 | 1.317e-06 | -0.951 |
| Immune | CD8_primary | H3K4me3 | 0.008 | -3.925e-07 | 6.955e-07 | -0.564 |
| Immune | Mobilized_CD34_primary | H3K4me3 | 0.014 | 2.353e-07 | 3.287e-07 | 0.716 |
| Immune | Peripheralblood_mononuclear_primary | H3K4me3 | 0.007 | -3.895e-07 | 9.063e-07 | -0.430 |
| Immune | Spleen | H3K4me3 | 0.003 | -1.022e-06 | 1.185e-06 | -0.862 |
| Immune | Treg_primary | H3K4me3 | 0.012 | 5.591e-08 | 3.338e-07 | 0.167 |
| Immune | CD8_naive_primary_UCSF_minUBC | H3K9ac | 0.002 | -3.679e-06 | 1.956e-06 | -1.881 |
| Immune | Peripheralblood_mononuclear_primary | H3K9ac | 0.009 | -3.512e-07 | 7.550e-07 | -0.465 |

From left to right, columns indicate 1) tissue from which the cell-types originate, 2) cell-type, 3) histone modification, 4) proportion of SNPs included in the annotation, 5) the regression coefficient, 6) the standard error of the coefficient, and 7) the corresponding Z-score. One asterisk indicates coefficients passing nomial significance (*p*<0.05)

**Table S8. Results of intersection between eQTLs and cell-type-specific differentially epigenetically modified regions for Rheumatoid arthritis**

| Tissue type | Tissue | Histone | Prop.SNPs | Coefficient | SE | Z |
| --- | --- | --- | --- | --- | --- | --- |
| CNS | Angular_gyrus | H3K27ac | 0.011 | 3.576e-08 | 6.548e-08 | 0.546 |
| CNS | Anterior_caudate | H3K27ac | 0.009 | -8.032e-09 | 7.873e-08 | -0.102 |
| CNS | Cingulate_gyrus | H3K27ac | 0.011 | 1.071e-08 | 6.438e-08 | 0.166 |
| CNS | Hippocampus_middle | H3K27ac | 0.010 | 4.895e-08 | 8.696e-08 | 0.563 |
| CNS | Inferior_temporal_lobe | H3K27ac | 0.011 | 3.957e-08 | 6.295e-08 | 0.629 |
| CNS | Mid_frontal_lobe | H3K27ac | 0.009 | -1.799e-09 | 7.953e-08 | -0.023 |
| CNS | Neurosphere | H3K27ac | 0.013 | 5.197e-09 | 7.540e-08 | 0.069 |
| CNS | Substantia_nigra | H3K27ac | 0.012 | 3.595e-08 | 6.301e-08 | 0.571 |
| CNS | Angular_gyrus | H3K4me1 | 0.007 | -2.860e-08 | 1.201e-07 | -0.238 |
| CNS | Anterior_caudate | H3K4me1 | 0.019 | 6.844e-09 | 6.051e-08 | 0.113 |
| CNS | Cingulate_gyrus | H3K4me1 | 0.020 | 1.554e-08 | 5.941e-08 | 0.262 |
| CNS | Fetal_brain | H3K4me1 | 0.024 | 4.310e-08 | 5.563e-08 | 0.775 |
| CNS | Fetal_thymus | H3K4me1 | 0.019 | -1.306e-07 | 9.336e-08 | -1.399 |
| CNS | Hippocampus_middle | H3K4me1 | 0.026 | -1.252e-08 | 5.075e-08 | -0.247 |
| CNS | Inferior_temporal_lobe | H3K4me1 | 0.024 | 1.578e-08 | 5.354e-08 | 0.295 |
| CNS | Mid_frontal_lobe | H3K4me1 | 0.002 | -1.629e-07 | 2.439e-07 | -0.668 |
| CNS | Substantia_nigra | H3K4me1 | 0.018 | 6.355e-09 | 5.968e-08 | 0.106 |
| CNS | Angular_gyrus | H3K4me3 | 0.007 | -5.034e-08 | 2.676e-07 | -0.188 |
| CNS | Anterior_caudate | H3K4me3 | 0.012 | -1.027e-07 | 1.673e-07 | -0.614 |
| CNS | Cingulate_gyrus | H3K4me3 | 0.011 | -1.327e-07 | 1.738e-07 | -0.764 |
| CNS | Fetal_brain | H3K4me3 | 0.003 | 9.566e-08 | 4.160e-07 | 0.230 |
| CNS | Fetal_thymus | H3K4me3 | 0.007 | -3.307e-07 | 3.656e-07 | -0.905 |
| CNS | Germinal_matrix | H3K4me3 | 0.008 | 4.808e-08 | 2.208e-07 | 0.218 |
| CNS | Hippocampus_middle | H3K4me3 | 0.012 | -1.170e-07 | 1.731e-07 | -0.676 |
| CNS | Inferior_temporal_lobe | H3K4me3 | 0.011 | -8.033e-08 | 1.815e-07 | -0.442 |
| CNS | Mid_frontal_lobe | H3K4me3 | 0.011 | -1.026e-07 | 1.910e-07 | -0.537 |

**Table S8 (continued). Results of intersection between eQTLs and cell-type-specific differentially epigenetically modified regions for Rheumatoid arthritis**

| Tissue type | Tissue | Histone | Prop.SNPs | Coefficient | SE | Z |
| --- | --- | --- | --- | --- | --- | --- |
| CNS | Substantia_nigra | H3K4me3 | 0.009 | -3.074e-07 | 2.062e-07 | -1.491 |
| CNS | Angular_gyrus | H3K9ac | 0.007 | 3.218e-08 | 2.105e-07 | 0.153 |
| CNS | Anterior_caudate | H3K9ac | 0.009 | -9.343e-08 | 1.871e-07 | -0.499 |
| CNS | Cingulate_gyrus | H3K9ac | 0.012 | -3.727e-09 | 1.298e-07 | -0.029 |
| CNS | Fetal_brain | H3K9ac | 0.009 | 1.293e-07 | 2.038e-07 | 0.635 |
| CNS | Hippocampus_middle | H3K9ac | 0.012 | 8.515e-09 | 1.326e-07 | 0.064 |
| CNS | Inferior_temporal_lobe | H3K9ac | 0.011 | 6.892e-08 | 1.389e-07 | 0.496 |
| CNS | Mid_frontal_lobe | H3K9ac | 0.010 | 4.192e-08 | 1.565e-07 | 0.268 |
| CNS | Substantia_nigra | H3K9ac | 0.011 | -3.825e-08 | 1.322e-07 | -0.289 |
| Immune | CD14 | H3K27ac | 0.015 | 7.335e-08 | 8.470e-08 | 0.866 |
| Immune | CD19 | H3K27ac | 0.017 | -1.755e-08 | 1.126e-07 | -0.156 |
| Immune | CD20 | H3K27ac | 0.016 | -4.156e-08 | 1.056e-07 | -0.394 |
| Immune | CD25-_CD45RA+_naive | H3K27ac | 0.016 | -3.845e-08 | 1.073e-07 | -0.358 |
| Immune | CD25-_IL17-_Th_stim_MACS | H3K27ac | 0.013 | -2.429e-07 | 1.386e-07 | -1.753 |
| Immune | CD25-_IL17+_Th17_stim | H3K27ac | 0.015 | -2.090e-07 | 1.939e-07 | -1.078 |
| Immune | CD25+_CD127-_Treg | H3K27ac | 0.018 | -6.370e-08 | 1.484e-07 | -0.429 |
| Immune | CD25int_CD127+_Tmem | H3K27ac | 0.017 | -1.394e-07 | 1.365e-07 | -1.021 |
| Immune | CD3_primary | H3K27ac | 0.016 | -1.104e-07 | 1.034e-07 | -1.069 |
| Immune | Mobilized_CD34 | H3K27ac | 0.017 | 2.719e-08 | 8.735e-08 | 0.311 |
| Immune | Th0 | H3K27ac | 0.015 | -2.288e-07 | 1.446e-07 | -1.583 |
| Immune | Th1 | H3K27ac | 0.015 | -2.352e-07 | 1.532e-07 | -1.535 |
| Immune | Th2 | H3K27ac | 0.014 | -2.765e-07 | 1.524e-07 | -1.814 |
| Immune | CD14_primary | H3K4me1 | 0.022 | -7.523e-08 | 9.331e-08 | -0.806 |
| Immune | CD15_primary | H3K4me1 | 0.021 | -3.884e-08 | 1.080e-07 | -0.360 |
| Immune | CD19_primary_BI | H3K4me1 | 0.024 | -5.550e-08 | 1.051e-07 | -0.528 |

**Table S8 (continued). Results of intersection between eQTLs and cell-type-specific differentially epigenetically modified regions for Rheumatoid arthritis**

| Tissue type | Tissue | Histone | Prop.SNPs | Coefficient | SE | Z |
| --- | --- | --- | --- | --- | --- | --- |
| Immune | CD19_primary_UW | H3K4me1 | 0.023 | -6.478e-08 | 1.023e-07 | -0.633 |
| Immune | CD3_primary_BI | H3K4me1 | 0.016 | -1.668e-07 | 1.100e-07 | -1.516 |
| Immune | CD3_primary_UW | H3K4me1 | 0.021 | -1.820e-07 | 1.108e-07 | -1.643 |
| Immune | CD34_primary | H3K4me1 | 0.018 | -9.181e-08 | 1.073e-07 | -0.855 |
| Immune | CD4_memory_primary | H3K4me1 | 0.024 | -1.111e-07 | 8.754e-08 | -1.270 |
| Immune | CD4_naive_primary | H3K4me1 | 0.019 | -1.619e-07 | 1.009e-07 | -1.604 |
| Immune | CD4+_CD25-_CD45R0+_memory_primary | H3K4me1 | 0.020 | -1.605e-07 | 1.090e-07 | -1.472 |
| Immune | CD4+_CD25-_CD45RA+_naive_primary | H3K4me1 | 0.022 | -1.142e-07 | 8.308e-08 | -1.375 |
| Immune | CD4+_CD25-_IL17-_PMA_Ionomycin_stim_MACS_Th_sprimary | H3K4me1 | 0.030 | -1.073e-07 | 6.891e-08 | -1.557 |
| Immune | CD4+_CD25-_IL17+_PMA_Ionomycin_stim_Th17_primary | H3K4me1 | 0.023 | -1.193e-07 | 1.017e-07 | -1.173 |
| Immune | CD4+_CD25-_Th_primary | H3K4me1 | 0.026 | -1.116e-07 | 8.589e-08 | -1.299 |
| Immune | CD4+_CD25+_CD127-_Treg_primary | H3K4me1 | 0.017 | -1.516e-07 | 1.459e-07 | -1.039 |
| Immune | CD4+_CD25int_CD127+_Tmem_primary | H3K4me1 | 0.009 | -3.220e-07 | 2.169e-07 | -1.485 |
| Immune | CD56_primary | H3K4me1 | 0.020 | -1.266e-07 | 1.088e-07 | -1.163 |
| Immune | CD8_memory_primary | H3K4me1 | 0.019 | -1.551e-07 | 1.042e-07 | -1.488 |
| Immune | CD8_naive_primary_BI | H3K4me1 | 0.019 | -1.515e-07 | 9.368e-08 | -1.617 |
| Immune | CD8_naive_primary_UCSF_minUBC | H3K4me1 | 0.015 | -1.868e-07 | 1.157e-07 | -1.615 |
| Immune | Mobilized_CD34_primary | H3K4me1 | 0.036 | -3.528e-08 | 5.735e-08 | -0.615 |
| Immune | Peripheralblood_mononuclear_primary | H3K4me1 | 0.006 | -6.776e-07 | 3.585e-07 | -1.890 |
| Immune | Spleen | H3K4me1 | 0.024 | -8.727e-08 | 7.855e-08 | -1.111 |
| Immune | Thymus | H3K4me1 | 0.005 | -1.794e-07 | 3.200e-07 | -0.561 |
| Immune | CD14_primary | H3K4me3 | 0.006 | 7.043e-08 | 3.341e-07 | 0.211 |
| Immune | CD15_primary | H3K4me3 | 0.010 | -2.479e-07 | 2.739e-07 | -0.905 |
| Immune | CD19_primary_BI | H3K4me3 | 0.009 | -1.601e-07 | 3.218e-07 | -0.498 |
| Immune | CD19_primary_UW | H3K4me3 | 0.007 | -8.637e-08 | 3.996e-07 | -0.216 |

**Table S8 (continued). Results of intersection between eQTLs and cell-type-specific differentially epigenetically modified regions for Rheumatoid arthritis**

| Tissue type | Tissue | Histone | Prop.SNPs | Coefficient | SE | Z |
| --- | --- | --- | --- | --- | --- | --- |
| Immune | CD3_primary_BI | H3K4me3 | 0.010 | -6.467e-07 | 2.520e-07 | -2.566 |
| Immune | CD3_primary_UW | H3K4me3 | 0.008 | -6.780e-07 | 3.962e-07 | -1.711 |
| Immune | CD34_primary | H3K4me3 | 0.008 | 1.048e-07 | 2.819e-07 | 0.372 |
| Immune | CD4_memory_primary | H3K4me3 | 0.008 | -7.286e-07 | 3.564e-07 | -2.044 |
| Immune | CD4_naive_primary | H3K4me3 | 0.009 | -7.876e-07 | 2.894e-07 | -2.722 |
| Immune | CD4+_CD25-_CD45R0+_memory_primary | H3K4me3 | 0.006 | -1.043e-06 | 3.980e-07 | -2.621 |
| Immune | CD4+_CD25-_CD45RA+_naive_primary | H3K4me3 | 0.009 | -5.910e-07 | 2.913e-07 | -2.029 |
| Immune | CD4+_CD25-_IL17-_PMA_Ionomycin_stim_MACS_Th_sprimary | H3K4me3 | 0.012 | -5.647e-07 | 2.382e-07 | -2.370 |
| Immune | CD4+_CD25-_IL17+_PMA_Ionomycin_stim_Th17_primary | H3K4me3 | 0.009 | -6.483e-07 | 4.124e-07 | -1.572 |
| Immune | CD4+_CD25-_Th_primary | H3K4me3 | 0.009 | -5.417e-07 | 3.338e-07 | -1.623 |
| Immune | CD4+_CD25+_CD127-_Treg_primary | H3K4me3 | 0.010 | -5.774e-07 | 3.496e-07 | -1.652 |
| Immune | CD4+_CD25int_CD127+_Tmem_primary | H3K4me3 | 0.008 | -7.642e-07 | 3.881e-07 | -1.969 |
| Immune | CD4_primary | H3K4me3 | 0.009 | -6.365e-07 | 3.307e-07 | -1.924 |
| Immune | CD56_primary | H3K4me3 | 0.006 | -4.879e-07 | 4.217e-07 | -1.157 |
| Immune | CD8_memory_primary | H3K4me3 | 0.008 | -8.118e-07 | 3.170e-07 | -2.561 |
| Immune | CD8_naive_primary_BI | H3K4me3 | 0.008 | -6.978e-07 | 3.252e-07 | -2.146 |
| Immune | CD8_naive_primary_UCSF_minUBC | H3K4me3 | 0.005 | -8.569e-07 | 5.098e-07 | -1.681 |
| Immune | CD8_primary | H3K4me3 | 0.008 | -5.375e-07 | 3.545e-07 | -1.516 |
| Immune | Mobilized_CD34_primary | H3K4me3 | 0.014 | -6.640e-08 | 1.790e-07 | -0.371 |
| Immune | Peripheralblood_mononuclear_primary | H3K4me3 | 0.007 | -6.799e-07 | 4.001e-07 | -1.699 |
| Immune | Spleen | H3K4me3 | 0.003 | 1.362e-07 | 5.380e-07 | 0.253 |
| Immune | Treg_primary | H3K4me3 | 0.012 | -3.735e-07 | 2.313e-07 | -1.614 |
| Immune | CD8_naive_primary_UCSF_minUBC | H3K9ac | 0.002 | -9.649e-07 | 9.446e-07 | -1.021 |
| Immune | Peripheralblood_mononuclear_primary | H3K9ac | 0.009 | -5.066e-07 | 2.785e-07 | -1.819 |

From left to right, columns indicate 1) tissue from which the cell-types originate, 2) cell-type, 3) histone modification, 4) proportion of SNPs included in the annotation, 5) the regression coefficient, 6) the standard error of the coefficient, and 7) the corresponding Z-score.

**Table S9. Results of cell-type-specific differentially epigenetically modified regions for schizophrenia**

| Tissue type | Tissue | Histone | Prop.SNPs | Coefficient | SE | Z |
| --- | --- | --- | --- | --- | --- | --- |
| CNS | Angular_gyrus | H3K27ac | 0.024 | 6.943e-07 | 1.545e-07 | 4.494*** |
| CNS | Anterior_caudate | H3K27ac | 0.022 | 4.663e-07 | 1.299e-07 | 3.588*** |
| CNS | Cingulate_gyrus | H3K27ac | 0.025 | 3.729e-07 | 1.419e-07 | 2.628** |
| CNS | Hippocampus_middle | H3K27ac | 0.020 | 3.042e-07 | 1.553e-07 | 1.959* |
| CNS | Inferior_temporal_lobe | H3K27ac | 0.026 | 6.007e-07 | 1.457e-07 | 4.122*** |
| CNS | Mid_frontal_lobe | H3K27ac | 0.019 | 1.055e-06 | 2.211e-07 | 4.771*** |
| CNS | Neurosphere | H3K27ac | 0.031 | 1.474e-07 | 1.219e-07 | 1.210 |
| CNS | Substantia_nigra | H3K27ac | 0.025 | 9.123e-08 | 1.222e-07 | 0.747 |
| CNS | Angular_gyrus | H3K4me1 | 0.013 | 4.945e-07 | 2.913e-07 | 1.697* |
| CNS | Anterior_caudate | H3K4me1 | 0.039 | 4.735e-07 | 1.283e-07 | 3.691*** |
| CNS | Cingulate_gyrus | H3K4me1 | 0.041 | 4.194e-07 | 1.375e-07 | 3.051** |
| CNS | Fetal_brain | H3K4me1 | 0.033 | 5.058e-07 | 1.346e-07 | 3.758*** |
| CNS | Fetal_thymus | H3K4me1 | 0.036 | -7.583e-08 | 1.286e-07 | -0.590 |
| CNS | Hippocampus_middle | H3K4me1 | 0.052 | 3.294e-07 | 1.235e-07 | 2.666** |
| CNS | Inferior_temporal_lobe | H3K4me1 | 0.048 | 5.234e-07 | 1.245e-07 | 4.206*** |
| CNS | Mid_frontal_lobe | H3K4me1 | 0.003 | 3.201e-07 | 6.386e-07 | 0.501 |
| CNS | Substantia_nigra | H3K4me1 | 0.038 | 2.109e-07 | 1.320e-07 | 1.598 |
| CNS | Angular_gyrus | H3K4me3 | 0.011 | 3.892e-06 | 6.244e-07 | 6.233*** |
| CNS | Anterior_caudate | H3K4me3 | 0.019 | 2.490e-06 | 3.702e-07 | 6.726*** |
| CNS | Cingulate_gyrus | H3K4me3 | 0.018 | 2.641e-06 | 4.385e-07 | 6.023*** |
| CNS | Fetal_brain | H3K4me3 | 0.004 | 4.680e-06 | 1.743e-06 | 2.684** |
| CNS | Fetal_thymus | H3K4me3 | 0.009 | 5.206e-07 | 5.415e-07 | 0.961 |
| CNS | Germinal_matrix | H3K4me3 | 0.012 | 4.280e-06 | 8.139e-07 | 5.258*** |
| CNS | Hippocampus_middle | H3K4me3 | 0.019 | 1.907e-06 | 3.968e-07 | 4.807*** |
| CNS | Inferior_temporal_lobe | H3K4me3 | 0.017 | 3.031e-06 | 4.548e-07 | 6.663*** |
| CNS | Mid_frontal_lobe | H3K4me3 | 0.017 | 3.565e-06 | 4.524e-07 | 7.881*** |

**Table S9 (continued). Results of cell-type-specific differentially epigenetically modified regions for schizophrenia**

| Tissue type | Tissue | Histone | Prop.SNPs | Coefficient | SE | Z |
| --- | --- | --- | --- | --- | --- | --- |
| CNS | Substantia_nigra | H3K4me3 | 0.014 | 1.794e-06 | 4.645e-07 | 3.862*** |
| CNS | Angular_gyrus | H3K9ac | 0.011 | 2.390e-06 | 5.076e-07 | 4.708*** |
| CNS | Anterior_caudate | H3K9ac | 0.015 | 1.842e-06 | 4.173e-07 | 4.413*** |
| CNS | Cingulate_gyrus | H3K9ac | 0.019 | 1.588e-06 | 3.224e-07 | 4.927*** |
| CNS | Fetal_brain | H3K9ac | 0.013 | 2.032e-06 | 4.764e-07 | 4.265*** |
| CNS | Hippocampus_middle | H3K9ac | 0.018 | 1.080e-06 | 3.151e-07 | 3.429*** |
| CNS | Inferior_temporal_lobe | H3K9ac | 0.017 | 1.245e-06 | 3.478e-07 | 3.580*** |
| CNS | Mid_frontal_lobe | H3K9ac | 0.015 | 2.139e-06 | 3.905e-07 | 5.479*** |
| CNS | Substantia_nigra | H3K9ac | 0.017 | 7.714e-07 | 3.118e-07 | 2.474** |
| Immune | CD14 | H3K27ac | 0.028 | -1.503e-07 | 1.146e-07 | -1.311 |
| Immune | CD19 | H3K27ac | 0.037 | 3.247e-07 | 1.307e-07 | 2.485** |
| Immune | CD20 | H3K27ac | 0.029 | 3.972e-07 | 1.522e-07 | 2.609** |
| Immune | CD25-_CD45RA+_naive | H3K27ac | 0.027 | 3.452e-07 | 2.447e-07 | 1.411 |
| Immune | CD25-_IL17-_Th_stim_MACS | H3K27ac | 0.023 | 1.845e-07 | 1.995e-07 | 0.925 |
| Immune | CD25-_IL17+_Th17_stim | H3K27ac | 0.027 | -1.433e-07 | 1.533e-07 | -0.935 |
| Immune | CD25+_CD127-_Treg | H3K27ac | 0.031 | 6.526e-09 | 1.755e-07 | 0.037 |
| Immune | CD25int_CD127+_Tmem | H3K27ac | 0.032 | -2.017e-07 | 1.829e-07 | -1.103 |
| Immune | CD3_primary | H3K27ac | 0.032 | 2.310e-08 | 1.380e-07 | 0.167 |
| Immune | Mobilized_CD34 | H3K27ac | 0.032 | -2.219e-08 | 1.381e-07 | -0.161 |
| Immune | Th0 | H3K27ac | 0.032 | -1.290e-07 | 1.113e-07 | -1.159 |
| Immune | Th1 | H3K27ac | 0.032 | -5.767e-08 | 1.152e-07 | -0.500 |
| Immune | Th2 | H3K27ac | 0.032 | -5.138e-08 | 1.184e-07 | -0.434 |
| Immune | CD14_primary | H3K4me1 | 0.040 | -2.687e-07 | 1.097e-07 | -2.450 |
| Immune | CD15_primary | H3K4me1 | 0.036 | -2.496e-08 | 1.072e-07 | -0.233 |
| Immune | CD19_primary_BI | H3K4me1 | 0.041 | 1.734e-07 | 1.324e-07 | 1.309 |
| Immune | CD19_primary_UW | H3K4me1 | 0.041 | 1.210e-07 | 1.251e-07 | 0.968 |

**Table S9 (continued). Results of cell-type-specific differentially epigenetically modified regions for schizophrenia**

| Tissue type | Tissue | Histone | Prop.SNPs | Coefficient | SE | Z |
| --- | --- | --- | --- | --- | --- | --- |
| Immune | CD3_primary_BI | H3K4me1 | 0.030 | 6.196e-08 | 1.663e-07 | 0.373 |
| Immune | CD3_primary_UW | H3K4me1 | 0.036 | 1.110e-07 | 1.561e-07 | 0.711 |
| Immune | CD34_primary | H3K4me1 | 0.032 | -1.565e-07 | 1.520e-07 | -1.030 |
| Immune | CD4_memory_primary | H3K4me1 | 0.041 | 6.978e-08 | 1.142e-07 | 0.611 |
| Immune | CD4_naive_primary | H3K4me1 | 0.032 | 1.305e-07 | 1.592e-07 | 0.820 |
| Immune | CD4+_CD25-_CD45R0+_memory_primary | H3K4me1 | 0.034 | 6.198e-08 | 1.359e-07 | 0.456 |
| Immune | CD4+_CD25-_CD45RA+_naive_primary | H3K4me1 | 0.038 | 1.694e-07 | 1.558e-07 | 1.088 |
| Immune | CD4+_CD25-_IL17-_PMA_Ionomycin_stim_MACS_Th_sprimary | H3K4me1 | 0.052 | 1.550e-07 | 1.162e-07 | 1.334 |
| Immune | CD4+_CD25-_IL17+_PMA_Ionomycin_stim_Th17_primary | H3K4me1 | 0.039 | 9.004e-08 | 1.183e-07 | 0.761 |
| Immune | CD4+_CD25-_Th_primary | H3K4me1 | 0.043 | 1.659e-07 | 1.408e-07 | 1.178 |
| Immune | CD4+_CD25+_CD127-_Treg_primary | H3K4me1 | 0.029 | 5.888e-08 | 1.826e-07 | 0.323 |
| Immune | CD4+_CD25int_CD127+_Tmem_primary | H3K4me1 | 0.016 | 4.113e-08 | 3.038e-07 | 0.135 |
| Immune | CD56_primary | H3K4me1 | 0.035 | 2.104e-09 | 1.432e-07 | 0.015 |
| Immune | CD8_memory_primary | H3K4me1 | 0.032 | 1.147e-07 | 1.582e-07 | 0.725 |
| Immune | CD8_naive_primary_BI | H3K4me1 | 0.032 | 1.885e-07 | 1.803e-07 | 1.045 |
| Immune | CD8_naive_primary_UCSF_minUBC | H3K4me1 | 0.025 | 2.666e-07 | 2.067e-07 | 1.290 |
| Immune | Mobilized_CD34_primary | H3K4me1 | 0.069 | -6.512e-08 | 8.956e-08 | -0.727 |
| Immune | Peripheralblood_mononuclear_primary | H3K4me1 | 0.009 | 3.303e-07 | 9.936e-07 | 0.332 |
| Immune | Spleen | H3K4me1 | 0.046 | -7.693e-08 | 1.254e-07 | -0.614 |
| Immune | Thymus | H3K4me1 | 0.009 | 7.628e-08 | 2.574e-07 | 0.296 |
| Immune | CD14_primary | H3K4me3 | 0.008 | 6.794e-07 | 6.180e-07 | 1.099 |
| Immune | CD15_primary | H3K4me3 | 0.014 | 9.060e-08 | 3.062e-07 | 0.296 |
| Immune | CD19_primary_BI | H3K4me3 | 0.012 | 9.726e-07 | 4.593e-07 | 2.118* |
| Immune | CD19_primary_UW | H3K4me3 | 0.009 | 1.537e-06 | 6.240e-07 | 2.463** |
| Immune | CD3_primary_BI | H3K4me3 | 0.014 | 6.060e-07 | 5.015e-07 | 1.208 |
| Immune | CD3_primary_UW | H3K4me3 | 0.011 | 1.044e-06 | 8.855e-07 | 1.179 |

**Table S9 (continued). Results of cell-type-specific differentially epigenetically modified regions for schizophrenia**

| Tissue type | Tissue | Histone | Prop.SNPs | Coefficient | SE | Z |
| --- | --- | --- | --- | --- | --- | --- |
| Immune | CD34_primary | H3K4me3 | 0.011 | 1.219e-06 | 5.362e-07 | 2.274** |
| Immune | CD4_memory_primary | H3K4me3 | 0.012 | 5.957e-07 | 7.508e-07 | 0.793 |
| Immune | CD4_naive_primary | H3K4me3 | 0.013 | 7.754e-07 | 7.610e-07 | 1.019 |
| Immune | CD4+_CD25-_CD45R0+_memory_primary | H3K4me3 | 0.009 | 1.206e-06 | 9.471e-07 | 1.273 |
| Immune | CD4+_CD25-_CD45RA+_naive_primary | H3K4me3 | 0.013 | 1.234e-06 | 9.227e-07 | 1.338 |
| Immune | CD4+_CD25-_IL17-_PMA_Ionomycin_stim_MACS_Th_sprimary | H3K4me3 | 0.017 | 6.397e-07 | 4.989e-07 | 1.282 |
| Immune | CD4+_CD25-_IL17+_PMA_Ionomycin_stim_Th17_primary | H3K4me3 | 0.013 | 7.085e-07 | 6.526e-07 | 1.086 |
| Immune | CD4+_CD25-_Th_primary | H3K4me3 | 0.013 | 1.298e-06 | 8.878e-07 | 1.462 |
| Immune | CD4+_CD25+_CD127-_Treg_primary | H3K4me3 | 0.014 | 6.204e-07 | 6.662e-07 | 0.931 |
| Immune | CD4+_CD25int_CD127+_Tmem_primary | H3K4me3 | 0.011 | 1.544e-06 | 1.001e-06 | 1.543 |
| Immune | CD4_primary | H3K4me3 | 0.013 | 8.178e-07 | 7.905e-07 | 1.035 |
| Immune | CD56_primary | H3K4me3 | 0.008 | 7.627e-07 | 5.486e-07 | 1.390 |
| Immune | CD8_memory_primary | H3K4me3 | 0.011 | 6.919e-07 | 8.943e-07 | 0.774 |
| Immune | CD8_naive_primary_BI | H3K4me3 | 0.011 | 1.428e-06 | 1.050e-06 | 1.360 |
| Immune | CD8_naive_primary_UCSF_minUBC | H3K4me3 | 0.006 | 2.436e-06 | 1.385e-06 | 1.758* |
| Immune | CD8_primary | H3K4me3 | 0.010 | 6.304e-07 | 7.210e-07 | 0.874 |
| Immune | Mobilized_CD34_primary | H3K4me3 | 0.023 | 4.351e-08 | 2.732e-07 | 0.159 |
| Immune | Peripheralblood_mononuclear_primary | H3K4me3 | 0.010 | 1.724e-06 | 1.004e-06 | 1.717* |
| Immune | Spleen | H3K4me3 | 0.004 | 2.806e-06 | 1.053e-06 | 2.666** |
| Immune | Treg_primary | H3K4me3 | 0.018 | 1.527e-07 | 2.878e-07 | 0.530 |
| Immune | CD8_naive_primary_UCSF_minUBC | H3K9ac | 0.002 | 4.223e-06 | 1.733e-06 | 2.437** |
| Immune | Peripheralblood_mononuclear_primary | H3K9ac | 0.013 | 1.271e-06 | 9.195e-07 | 1.382 |

From left to right, columns indicate 1) tissue from which the cell-types originate, 2) cell-type, 3) histone modification, 4) proportion of SNPs included in the annotation, 5) the regression coefficient, 6) the standard error of the coefficient, and 7) the corresponding Z-score. One asterisk indicates annotations reaching nomial significance (*p*<0.05). Two asterisks indicate annotations passing FDR correction for multiple testing. Three asterisks indicate annotations passing Bonferroni correction for multiple testing.

**Table S10. Results of cell-type-specific differentially epigenetically modified regions for rheumatoid arthritis**

| Tissue type | Tissue | Histone | Prop.SNPs | Coefficient | SE | Z |
| --- | --- | --- | --- | --- | --- | --- |
| CNS | Angular_gyrus | H3K27ac | 0.024 | -1.033e-07 | 3.428e-08 | -3.013 |
| CNS | Anterior_caudate | H3K27ac | 0.022 | -5.277e-08 | 4.252e-08 | -1.241 |
| CNS | Cingulate_gyrus | H3K27ac | 0.025 | -4.908e-08 | 3.391e-08 | -1.447 |
| CNS | Hippocampus_middle | H3K27ac | 0.020 | -4.112e-08 | 4.059e-08 | -1.013 |
| CNS | Inferior_temporal_lobe | H3K27ac | 0.026 | -8.970e-08 | 3.377e-08 | -2.656 |
| CNS | Mid_frontal_lobe | H3K27ac | 0.019 | -1.103e-07 | 4.765e-08 | -2.315 |
| CNS | Neurosphere | H3K27ac | 0.031 | -9.939e-08 | 3.555e-08 | -2.795 |
| CNS | Substantia_nigra | H3K27ac | 0.025 | -5.674e-08 | 3.091e-08 | -1.836 |
| CNS | Angular_gyrus | H3K4me1 | 0.013 | -7.439e-08 | 9.234e-08 | -0.806 |
| CNS | Anterior_caudate | H3K4me1 | 0.039 | -6.031e-08 | 4.433e-08 | -1.361 |
| CNS | Cingulate_gyrus | H3K4me1 | 0.041 | -6.760e-08 | 3.918e-08 | -1.725 |
| CNS | Fetal_brain | H3K4me1 | 0.033 | -7.948e-08 | 4.324e-08 | -1.838 |
| CNS | Fetal_thymus | H3K4me1 | 0.036 | 1.826e-07 | 5.701e-08 | 3.203** |
| CNS | Hippocampus_middle | H3K4me1 | 0.052 | -6.430e-08 | 3.531e-08 | -1.821 |
| CNS | Inferior_temporal_lobe | H3K4me1 | 0.048 | -8.856e-08 | 3.473e-08 | -2.550 |
| CNS | Mid_frontal_lobe | H3K4me1 | 0.003 | -2.832e-07 | 2.147e-07 | -1.319 |
| CNS | Substantia_nigra | H3K4me1 | 0.038 | -5.176e-08 | 3.868e-08 | -1.338 |
| CNS | Angular_gyrus | H3K4me3 | 0.011 | -3.636e-07 | 2.285e-07 | -1.591 |
| CNS | Anterior_caudate | H3K4me3 | 0.019 | -2.741e-07 | 1.371e-07 | -2.000 |
| CNS | Cingulate_gyrus | H3K4me3 | 0.018 | -2.337e-07 | 1.433e-07 | -1.631 |
| CNS | Fetal_brain | H3K4me3 | 0.004 | -5.063e-07 | 3.025e-07 | -1.674 |
| CNS | Fetal_thymus | H3K4me3 | 0.009 | 3.740e-07 | 3.089e-07 | 1.211 |
| CNS | Germinal_matrix | H3K4me3 | 0.012 | -4.488e-07 | 1.669e-07 | -2.689 |
| CNS | Hippocampus_middle | H3K4me3 | 0.019 | -2.687e-07 | 1.438e-07 | -1.868 |
| CNS | Inferior_temporal_lobe | H3K4me3 | 0.017 | -2.463e-07 | 1.339e-07 | -1.839 |
| CNS | Mid_frontal_lobe | H3K4me3 | 0.017 | -2.419e-07 | 1.500e-07 | -1.612 |

**Table S10 (continued). Results of cell-type-specific differentially epigenetically modified regions for rheumatoid arthritis**

| Tissue type | Tissue | Histone | Prop.SNPs | Coefficient | SE | Z |
| --- | --- | --- | --- | --- | --- | --- |
| CNS | Substantia_nigra | H3K4me3 | 0.014 | -1.319e-07 | 1.821e-07 | -0.724 |
| CNS | Angular_gyrus | H3K9ac | 0.011 | -3.038e-07 | 1.478e-07 | -2.055 |
| CNS | Anterior_caudate | H3K9ac | 0.015 | -1.646e-07 | 1.325e-07 | -1.242 |
| CNS | Cingulate_gyrus | H3K9ac | 0.019 | -2.010e-07 | 9.945e-08 | -2.022 |
| CNS | Fetal_brain | H3K9ac | 0.013 | -4.183e-07 | 1.541e-07 | -2.715 |
| CNS | Hippocampus_middle | H3K9ac | 0.018 | -1.386e-07 | 1.016e-07 | -1.365 |
| CNS | Inferior_temporal_lobe | H3K9ac | 0.017 | -1.677e-07 | 9.882e-08 | -1.697 |
| CNS | Mid_frontal_lobe | H3K9ac | 0.015 | -2.454e-07 | 1.184e-07 | -2.073 |
| CNS | Substantia_nigra | H3K9ac | 0.017 | -1.071e-07 | 9.771e-08 | -1.096 |
| Immune | CD14 | H3K27ac | 0.028 | -5.654e-08 | 5.799e-08 | -0.975 |
| Immune | CD19 | H3K27ac | 0.037 | 1.487e-07 | 6.735e-08 | 2.208** |
| Immune | CD20 | H3K27ac | 0.029 | 1.749e-07 | 6.434e-08 | 2.719** |
| Immune | CD25-_CD45RA+_naive | H3K27ac | 0.027 | 1.543e-07 | 7.224e-08 | 2.136** |
| Immune | CD25-_IL17-_Th_stim_MACS | H3K27ac | 0.023 | 4.638e-07 | 1.009e-07 | 4.598*** |
| Immune | CD25-_IL17+_Th17_stim | H3K27ac | 0.027 | 5.741e-07 | 1.404e-07 | 4.089*** |
| Immune | CD25+_CD127-_Treg | H3K27ac | 0.031 | 3.423e-07 | 9.567e-08 | 3.578*** |
| Immune | CD25int_CD127+_Tmem | H3K27ac | 0.032 | 2.462e-07 | 1.008e-07 | 2.442** |
| Immune | CD3_primary | H3K27ac | 0.032 | 2.692e-07 | 5.632e-08 | 4.780*** |
| Immune | Mobilized_CD34 | H3K27ac | 0.032 | -3.403e-08 | 5.825e-08 | -0.584 |
| Immune | Th0 | H3K27ac | 0.032 | 4.583e-07 | 9.826e-08 | 4.664*** |
| Immune | Th1 | H3K27ac | 0.032 | 4.833e-07 | 1.038e-07 | 4.658*** |
| Immune | Th2 | H3K27ac | 0.032 | 5.266e-07 | 1.086e-07 | 4.850*** |
| Immune | CD14_primary | H3K4me1 | 0.040 | 1.006e-07 | 6.740e-08 | 1.493 |
| Immune | CD15_primary | H3K4me1 | 0.036 | 8.093e-08 | 8.137e-08 | 0.994 |
| Immune | CD19_primary_BI | H3K4me1 | 0.041 | 2.457e-07 | 6.897e-08 | 3.563*** |
| Immune | CD19_primary_UW | H3K4me1 | 0.041 | 2.654e-07 | 6.361e-08 | 4.172*** |

**Table S10 (continued). Results of cell-type-specific differentially epigenetically modified regions for rheumatoid arthritis**

| Tissue type | Tissue | Histone | Prop.SNPs | Coefficient | SE | Z |
| --- | --- | --- | --- | --- | --- | --- |
| Immune | CD3_primary_BI | H3K4me1 | 0.030 | 3.136e-07 | 7.004e-08 | 4.477*** |
| Immune | CD3_primary_UW | H3K4me1 | 0.036 | 3.533e-07 | 8.496e-08 | 4.158*** |
| Immune | CD34_primary | H3K4me1 | 0.032 | 1.047e-07 | 7.557e-08 | 1.385 |
| Immune | CD4_memory_primary | H3K4me1 | 0.041 | 2.936e-07 | 6.228e-08 | 4.714*** |
| Immune | CD4_naive_primary | H3K4me1 | 0.032 | 3.370e-07 | 6.961e-08 | 4.842*** |
| Immune | CD4+_CD25-_CD45R0+_memory_primary | H3K4me1 | 0.034 | 3.521e-07 | 7.633e-08 | 4.613*** |
| Immune | CD4+_CD25-_CD45RA+_naive_primary | H3K4me1 | 0.038 | 2.476e-07 | 5.461e-08 | 4.533*** |
| Immune | CD4+_CD25-_IL17-_PMA_Ionomycin_stim_MACS_Th_sprimary | H3K4me1 | 0.052 | 2.531e-07 | 4.823e-08 | 5.248*** |
| Immune | CD4+_CD25-_IL17+_PMA_Ionomycin_stim_Th17_primary | H3K4me1 | 0.039 | 3.556e-07 | 7.226e-08 | 4.921*** |
| Immune | CD4+_CD25-_Th_primary | H3K4me1 | 0.043 | 2.680e-07 | 6.020e-08 | 4.452*** |
| Immune | CD4+_CD25+_CD127-_Treg_primary | H3K4me1 | 0.029 | 4.698e-07 | 1.020e-07 | 4.604*** |
| Immune | CD4+_CD25int_CD127+_Tmem_primary | H3K4me1 | 0.016 | 6.269e-07 | 1.579e-07 | 3.972*** |
| Immune | CD56_primary | H3K4me1 | 0.035 | 3.057e-07 | 8.622e-08 | 3.546*** |
| Immune | CD8_memory_primary | H3K4me1 | 0.032 | 3.463e-07 | 7.334e-08 | 4.721*** |
| Immune | CD8_naive_primary_BI | H3K4me1 | 0.032 | 2.984e-07 | 6.085e-08 | 4.904*** |
| Immune | CD8_naive_primary_UCSF_minUBC | H3K4me1 | 0.025 | 3.639e-07 | 7.944e-08 | 4.581*** |
| Immune | Mobilized_CD34_primary | H3K4me1 | 0.069 | 5.941e-08 | 3.946e-08 | 1.505 |
| Immune | Peripheralblood_mononuclear_primary | H3K4me1 | 0.009 | 9.200e-07 | 2.909e-07 | 3.163** |
| Immune | Spleen | H3K4me1 | 0.046 | 9.473e-08 | 6.121e-08 | 1.548 |
| Immune | Thymus | H3K4me1 | 0.009 | 4.338e-07 | 1.785e-07 | 2.430** |
| Immune | CD14_primary | H3K4me3 | 0.008 | -3.631e-07 | 2.811e-07 | -1.291 |
| Immune | CD15_primary | H3K4me3 | 0.014 | 2.675e-07 | 2.240e-07 | 1.195 |
| Immune | CD19_primary_BI | H3K4me3 | 0.012 | 3.942e-07 | 2.578e-07 | 1.529 |
| Immune | CD19_primary_UW | H3K4me3 | 0.009 | 4.722e-07 | 3.624e-07 | 1.303 |
| Immune | CD3_primary_BI | H3K4me3 | 0.014 | 6.403e-07 | 2.336e-07 | 2.741** |
| Immune | CD3_primary_UW | H3K4me3 | 0.011 | 9.280e-07 | 3.643e-07 | 2.547** |

**Table S10 (continued). Results of cell-type-specific differentially epigenetically modified regions for rheumatoid arthritis**

| Tissue type | Tissue | Histone | Prop.SNPs | Coefficient | SE | Z |
| --- | --- | --- | --- | --- | --- | --- |
| Immune | CD34_primary | H3K4me3 | 0.011 | -1.430e-07 | 2.277e-07 | -0.628 |
| Immune | CD4_memory_primary | H3K4me3 | 0.012 | 9.856e-07 | 3.097e-07 | 3.182** |
| Immune | CD4_naive_primary | H3K4me3 | 0.013 | 8.876e-07 | 2.483e-07 | 3.575*** |
| Immune | CD4+_CD25-_CD45R0+_memory_primary | H3K4me3 | 0.009 | 1.313e-06 | 3.456e-07 | 3.798*** |
| Immune | CD4+_CD25-_CD45RA+_naive_primary | H3K4me3 | 0.013 | 7.623e-07 | 2.495e-07 | 3.055** |
| Immune | CD4+_CD25-_IL17-_PMA_Ionomycin_stim_MACS_Th_sprimary | H3K4me3 | 0.017 | 8.349e-07 | 2.120e-07 | 3.938*** |
| Immune | CD4+_CD25-_IL17+_PMA_Ionomycin_stim_Th17_primary | H3K4me3 | 0.013 | 1.340e-06 | 3.778e-07 | 3.547*** |
| Immune | CD4+_CD25-_Th_primary | H3K4me3 | 0.013 | 7.465e-07 | 2.836e-07 | 2.632** |
| Immune | CD4+_CD25+_CD127-_Treg_primary | H3K4me3 | 0.014 | 1.089e-06 | 3.331e-07 | 3.269** |
| Immune | CD4+_CD25int_CD127+_Tmem_primary | H3K4me3 | 0.011 | 1.057e-06 | 3.392e-07 | 3.116** |
| Immune | CD4_primary | H3K4me3 | 0.013 | 9.037e-07 | 2.948e-07 | 3.065** |
| Immune | CD56_primary | H3K4me3 | 0.008 | 7.575e-07 | 3.414e-07 | 2.219** |
| Immune | CD8_memory_primary | H3K4me3 | 0.011 | 9.795e-07 | 2.740e-07 | 3.575*** |
| Immune | CD8_naive_primary_BI | H3K4me3 | 0.011 | 7.682e-07 | 2.731e-07 | 2.813** |
| Immune | CD8_naive_primary_UCSF_minUBC | H3K4me3 | 0.006 | 7.521e-07 | 4.817e-07 | 1.561 |
| Immune | CD8_primary | H3K4me3 | 0.010 | 6.658e-07 | 3.049e-07 | 2.184** |
| Immune | Mobilized_CD34_primary | H3K4me3 | 0.023 | 1.872e-08 | 1.334e-07 | 0.140 |
| Immune | Peripheralblood_mononuclear_primary | H3K4me3 | 0.010 | 8.455e-07 | 3.431e-07 | 2.464** |
| Immune | Spleen | H3K4me3 | 0.004 | -3.601e-07 | 4.285e-07 | -0.840 |
| Immune | Treg_primary | H3K4me3 | 0.018 | 7.087e-07 | 1.823e-07 | 3.887*** |
| Immune | CD8_naive_primary_UCSF_minUBC | H3K9ac | 0.002 | 1.673e-06 | 9.061e-07 | 1.847* |
| Immune | Peripheralblood_mononuclear_primary | H3K9ac | 0.013 | 8.021e-07 | 2.324e-07 | 3.452*** |

From left to right, columns indicate 1) tissue from which the cell-types originate, 2) cell-type, 3) histone modification, 4) proportion of SNPs included in the annotation, 5) the regression coefficient, 6) the standard error of the coefficient, and 7) the corresponding Z-score. One asterisk indicates annotations reaching nomial significance (*p*<0.05). Two asterisks indicate annotations passing FDR correction for multiple testing. Three asterisks indicate annotations passing Bonferroni correction for multiple testing.

**Table S11. Results of eQTLs for top10% most strongly differentially expressed genes per tissue**

|  |  | Rheumatoid arthritis | | | Schizophrenia | | |
| --- | --- | --- | --- | --- | --- | --- | --- |
| Tissue | Prop.SNPs | Coefficient | SE | Z | Coefficient | SE | Z |
| Adipose_Subcutaneous | 0.015 | -5.730e-09 | 1.635e-08 | -0.351 | -4.724e-08 | 5.661e-08 | -0.835 |
| Adipose_Visceral_Omentum | 0.005 | -5.872e-08 | 2.529e-08 | -2.321 | -1.644e-07 | 9.625e-08 | -1.708 |
| Adrenal_Gland | 0.006 | -5.096e-09 | 2.269e-08 | -0.225 | -1.614e-09 | 8.742e-08 | -0.018 |
| Artery_Aorta | 0.011 | -2.720e-09 | 1.894e-08 | -0.144 | 2.305e-09 | 6.790e-08 | 0.034 |
| Artery_Coronary | 0.002 | 3.110e-08 | 3.719e-08 | 0.836 | -2.571e-07 | 8.087e-08 | -3.179 |
| Artery_Tibial | 0.016 | 6.666e-09 | 1.874e-08 | 0.356 | -6.888e-08 | 5.874e-08 | -1.173 |
| Brain_Anterior_cingulate_cortex_BA24 | 0.001 | -8.115e-08 | 3.719e-08 | -2.182 | 5.061e-08 | 2.814e-07 | 0.180 |
| Brain_Caudate_basal_ganglia | 0.002 | -2.638e-08 | 3.942e-08 | -0.669 | 2.996e-07 | 2.721e-07 | 1.101 |
| Brain_Cerebellar_Hemisphere | 0.006 | -1.564e-08 | 2.080e-08 | -0.752 | 2.288e-07 | 1.431e-07 | 1.599 |
| Brain_Cerebellum | 0.008 | -3.974e-09 | 2.030e-08 | -0.196 | 2.427e-07 | 1.124e-07 | 2.160* |
| Brain_Cortex | 0.002 | -3.348e-08 | 3.324e-08 | -1.007 | 3.204e-07 | 2.341e-07 | 1.369 |
| Brain_Frontal_Cortex_BA9 | 0.002 | -3.806e-08 | 3.319e-08 | -1.147 | 1.715e-07 | 2.526e-07 | 0.679 |
| Brain_Hippocampus | 0.001 | -3.989e-10 | 5.069e-08 | -0.008 | 3.672e-07 | 4.730e-07 | 0.776 |
| Brain_Hypothalamus | 0.001 | -6.279e-08 | 3.543e-08 | -1.772 | 4.470e-07 | 5.352e-07 | 0.835 |
| Brain_Nucleus_accumbens_basal_ganglia | 0.002 | -9.337e-09 | 7.134e-08 | -0.131 | -6.728e-08 | 1.669e-07 | -0.403 |
| Brain_Putamen_basal_ganglia | 0.001 | 1.202e-08 | 7.775e-08 | 0.155 | 3.846e-07 | 3.461e-07 | 1.111 |
| Breast_Mammary_Tissue | 0.005 | -5.365e-08 | 1.982e-08 | -2.707 | -1.139e-07 | 8.071e-08 | -1.411 |
| Cells_EBV-transformed_lymphocytes | 0.007 | -4.452e-09 | 2.438e-08 | -0.183 | -1.117e-07 | 7.292e-08 | -1.532 |
| Cells_Transformed_fibroblasts | 0.021 | -1.281e-08 | 1.224e-08 | -1.046 | -4.662e-08 | 4.652e-08 | -1.002 |
| Colon_Sigmoid | 0.004 | -2.710e-08 | 2.197e-08 | -1.233 | -7.252e-08 | 1.483e-07 | -0.489 |
| Colon_Transverse | 0.007 | 2.563e-08 | 4.333e-08 | 0.592 | 1.177e-07 | 1.012e-07 | 1.163 |
| Esophagus_Gastroesophageal_Junction | 0.003 | -3.079e-08 | 2.791e-08 | -1.103 | 1.357e-07 | 2.381e-07 | 0.570 |
| Esophagus_Mucosa | 0.014 | 1.021e-08 | 2.187e-08 | 0.467 | -3.009e-09 | 5.399e-08 | -0.056 |
| Esophagus_Muscularis | 0.012 | -2.709e-08 | 1.559e-08 | -1.737 | 9.857e-08 | 8.263e-08 | 1.193 |
| Heart_Atrial_Appendage | 0.007 | 1.652e-08 | 3.197e-08 | 0.517 | -1.682e-07 | 6.204e-08 | -2.712 |

**Table S11 (continued). Results of eQTLs for top10% most strongly differentially expressed genes per tissue**

|  |  | Rheumatoid arthritis | | | Schizophrenia | | |
| --- | --- | --- | --- | --- | --- | --- | --- |
| Tissue | Prop.SNPs | Coefficient | SE | Z | Coefficient | SE | Z |
| Heart_Left_Ventricle | 0.010 | -5.879e-10 | 1.686e-08 | -0.035 | -1.559e-07 | 6.313e-08 | -2.470 |
| Liver | 0.003 | 1.608e-07 | 1.091e-07 | 1.474 | -1.271e-07 | 1.080e-07 | -1.177 |
| Lung | 0.012 | -2.972e-09 | 2.431e-08 | -0.122 | 2.560e-08 | 7.923e-08 | 0.323 |
| Muscle_Skeletal | 0.016 | 2.610e-09 | 1.640e-08 | 0.159 | 4.613e-08 | 6.176e-08 | 0.747 |
| Nerve_Tibial | 0.021 | 1.377e-08 | 1.452e-08 | 0.948 | -6.392e-08 | 4.235e-08 | -1.509 |
| Ovary | 0.003 | 2.910e-08 | 3.073e-08 | 0.947 | -4.072e-08 | 1.530e-07 | -0.266 |
| Pancreas | 0.005 | 7.670e-08 | 5.990e-08 | 1.280 | 2.953e-08 | 8.917e-08 | 0.331 |
| Pituitary | 0.003 | 7.489e-09 | 2.814e-08 | 0.266 | -2.113e-07 | 8.241e-08 | -2.564 |
| Prostate | 0.002 | -2.865e-09 | 5.053e-08 | -0.057 | -3.222e-08 | 2.552e-07 | -0.126 |
| Skin_Not_Sun_Exposed_Suprapubic | 0.007 | -7.256e-09 | 2.984e-08 | -0.243 | 2.674e-09 | 6.893e-08 | 0.039 |
| Skin_Sun_Exposed_Lower_leg | 0.015 | 3.282e-09 | 2.430e-08 | 0.135 | 6.688e-09 | 5.036e-08 | 0.133 |
| Small_Intestine_Terminal_Ileum | 0.002 | 3.520e-08 | 6.806e-08 | 0.517 | 5.652e-08 | 1.840e-07 | 0.307 |
| Spleen | 0.005 | 3.952e-09 | 4.295e-08 | 0.092 | -8.153e-08 | 1.317e-07 | -0.619 |
| Stomach | 0.005 | 2.054e-08 | 4.999e-08 | 0.411 | 7.799e-08 | 1.364e-07 | 0.572 |
| Testis | 0.016 | 4.114e-09 | 1.231e-08 | 0.334 | -3.972e-08 | 4.589e-08 | -0.865 |
| Thyroid | 0.025 | -4.043e-09 | 1.485e-08 | -0.272 | 3.261e-08 | 5.752e-08 | 0.567 |
| Uterus | 0.001 | -4.183e-08 | 3.615e-08 | -1.157 | -2.634e-07 | 1.051e-07 | -2.507 |
| Vagina | 0.001 | 4.895e-08 | 6.267e-08 | 0.781 | -3.625e-07 | 1.575e-07 | -2.302 |
| Whole_Blood | 0.013 | 5.820e-08 | 3.867e-08 | 1.505 | 2.490e-08 | 6.802e-08 | 0.366 |

From left to right, columns show 1) tissue from which eQTLs were obtained, 2) the proportion of SNPs included in each annotation, 3) the regression coefficient for rheumatoid arthritis, 4) the standard error of the coefficient, 5) the corresponding Z-score, and the last three columns repeated for schizophrenia. One asterisk indicates categories reaching nomial significance (*p*<0.05).

**Figure S1. Comparison of enrichment in GWAS signal across eQTL annotations with various inclusion criteria**

Every dot represents one of the 11 traits. X-axis corresponds to the enrichment of the annotation labeled at the top of the plots. Y-axis corresponds to the enrichment of the annotation labeled at the left of the plots. Red line is an identity line.

**Figure S2. Comparison of coefficient Z-scores across annotations with various inclusion criteria**

Every dot represents one of the 11 traits. X-axis corresponds to the enrichment of the annotation labeled at the top of the plots. Y-axis corresponds to the enrichment of the annotation labeled at the left of the plots. Red line is an identity line.

**Figure S3 . Comparison of public dataset and datasets excluding participants from the NTR/NESDA.**

Vertical axis represents coefficient Z-scores.

**Figure S4. Distribution of mean correlation across genes within blood probe sets**

**Figure S5. Distribution of mean correlation across genes within brain probe sets**

**Figure S6. Distribution of mean correlation across genes across blood and brain probe sets**
