# Supplementary figures and images for: Characterizing the Relation Between Expression QTLs and Complex Traits: Exploring the Role of Tissue Specificity
Source: Behav Genet. 2018 Jul 20;48(5):374–85. doi: 10.1007/s10519-018-9914-2 (PMC6097736; doi:10.1007/s10519-018-9914-2)

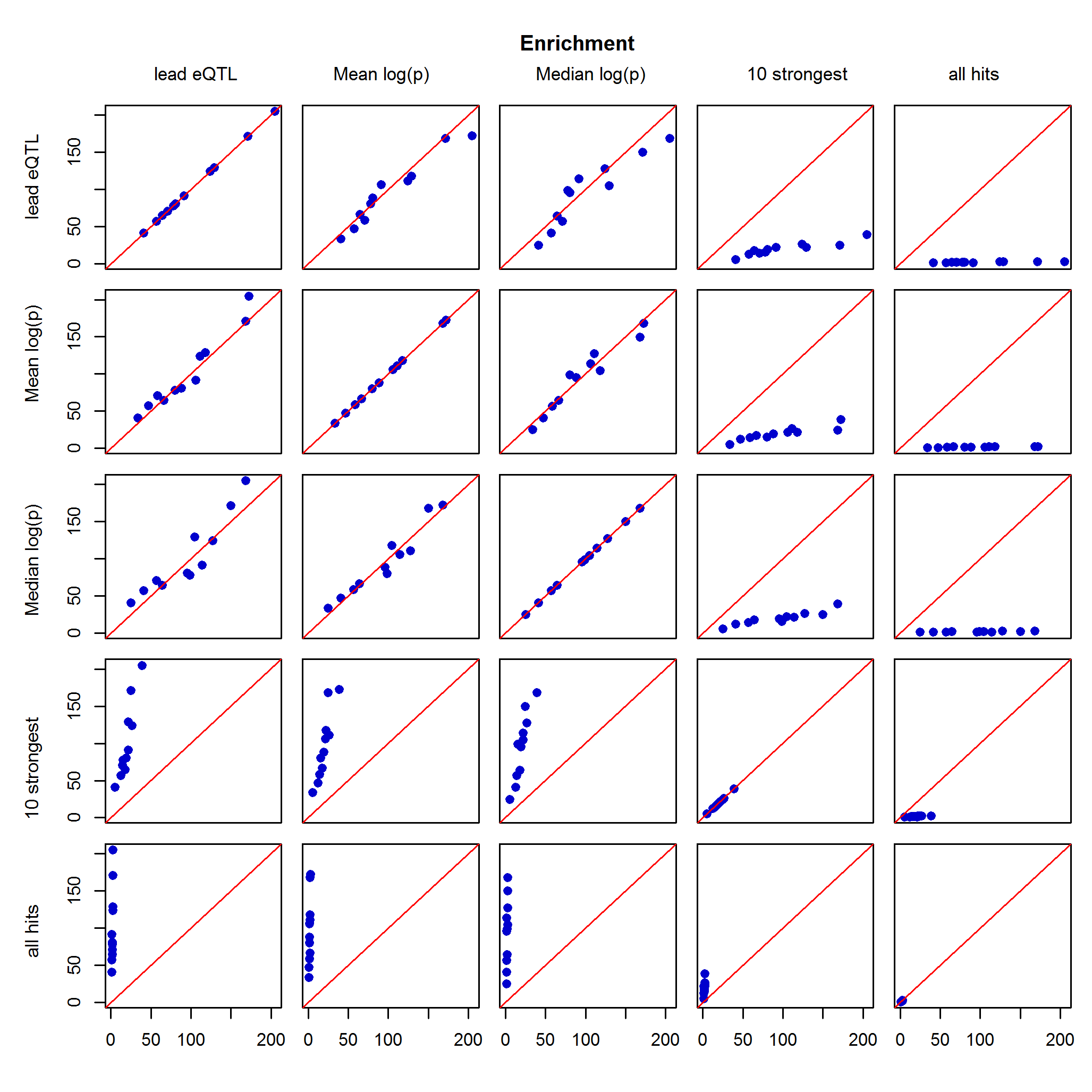

Supplement: Supplementary file 2 — Figure S1 (TIFF 12372 KB) [file 10519_2018_9914_MOESM2_ESM.tiff]

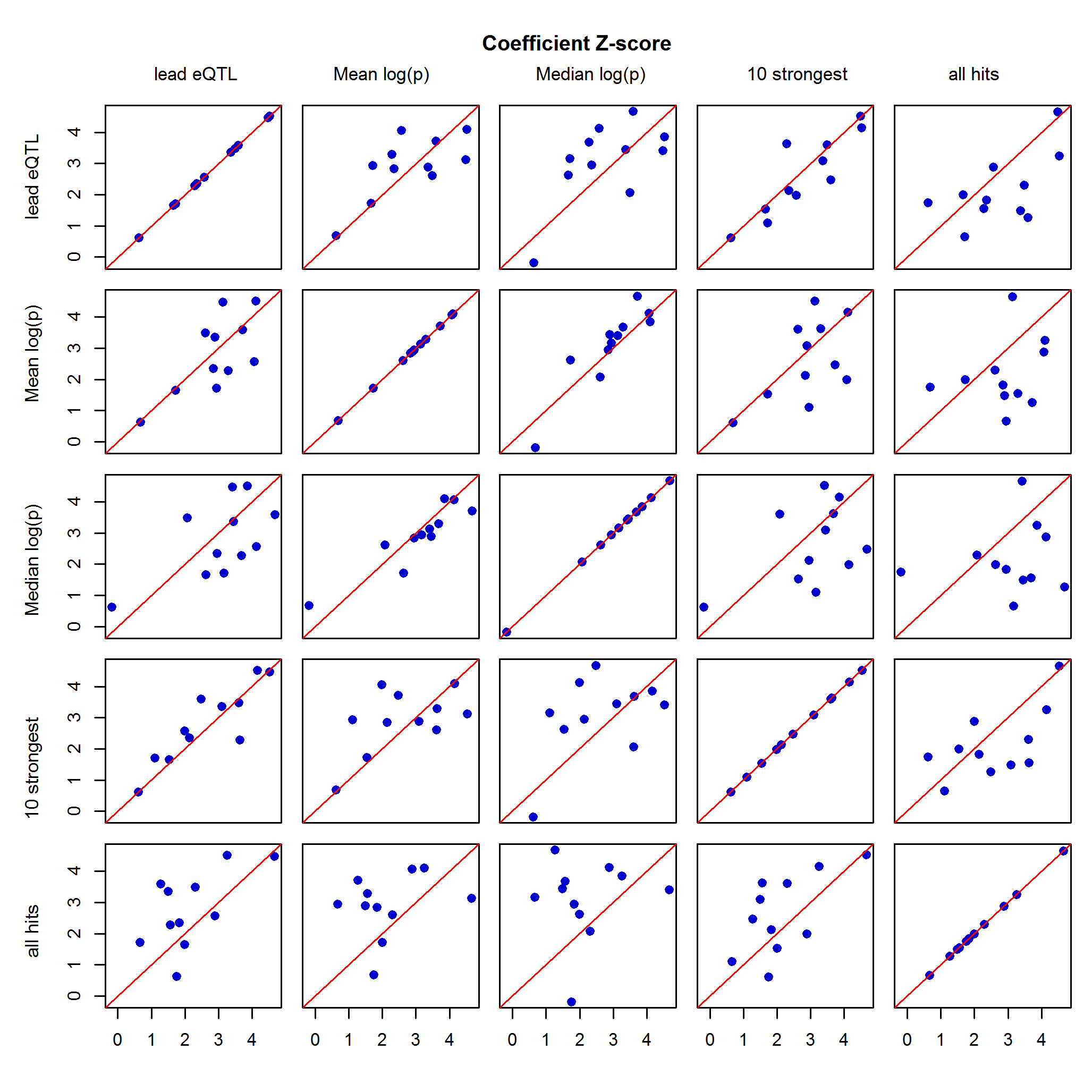

Supplement: Supplementary file 3 — Figure S2 (TIFF 12372 KB) [file 10519_2018_9914_MOESM3_ESM.tiff]

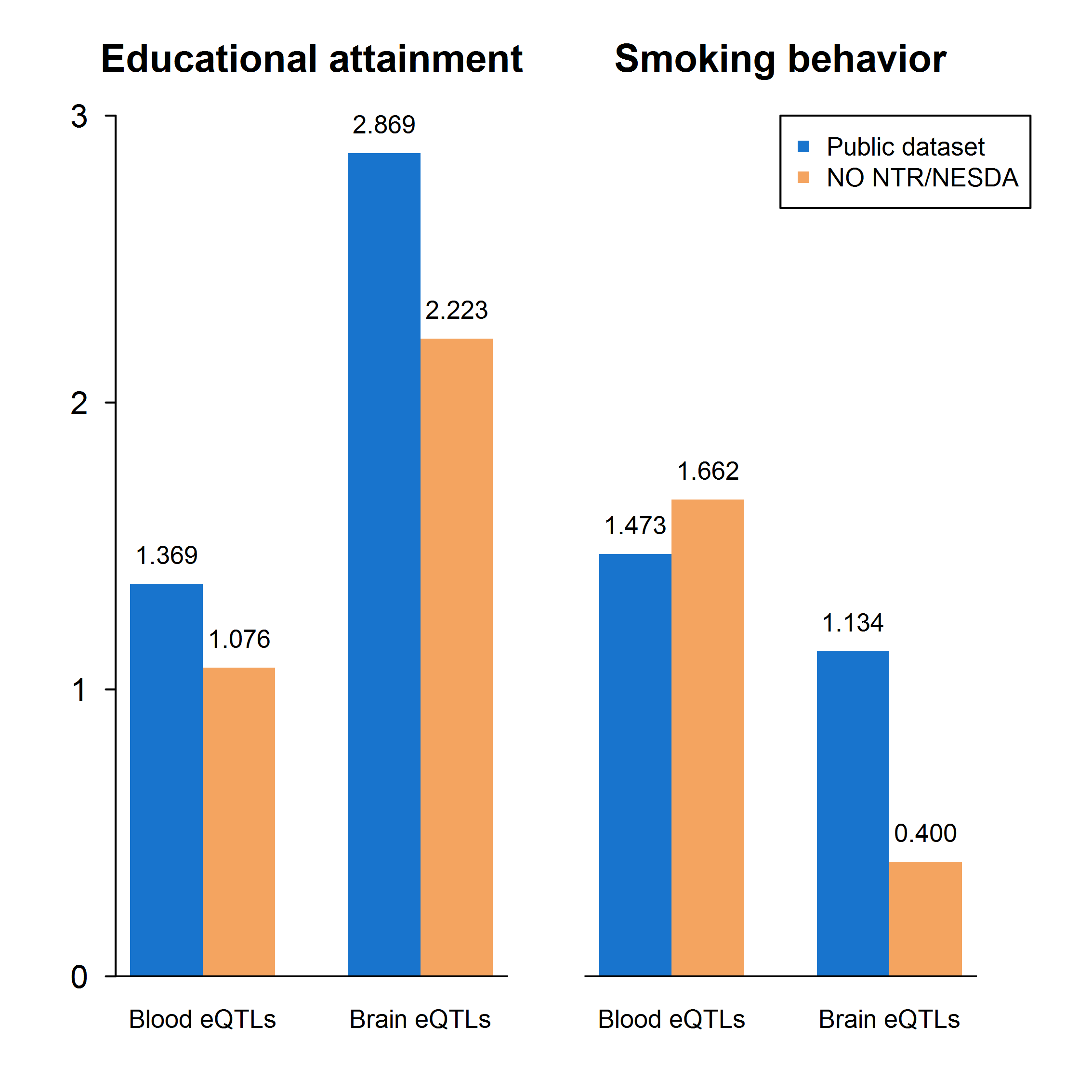

Supplement: Supplementary file 4 — Figure S3 (TIFF 15056 KB) [file 10519_2018_9914_MOESM4_ESM.tiff]

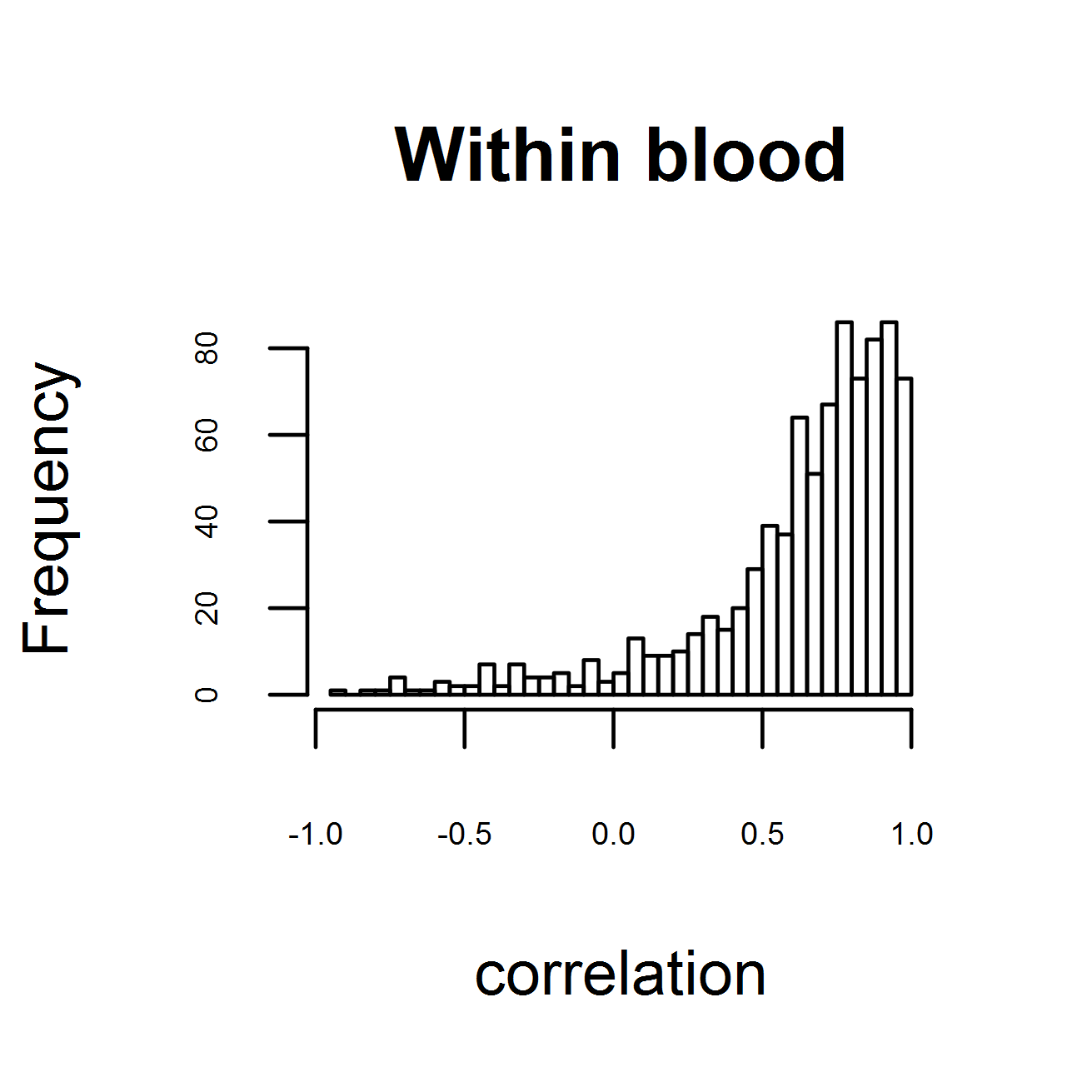

Supplement: Supplementary file 5 — Figure S4 (TIFF 5035 KB) [file 10519_2018_9914_MOESM5_ESM.tiff]

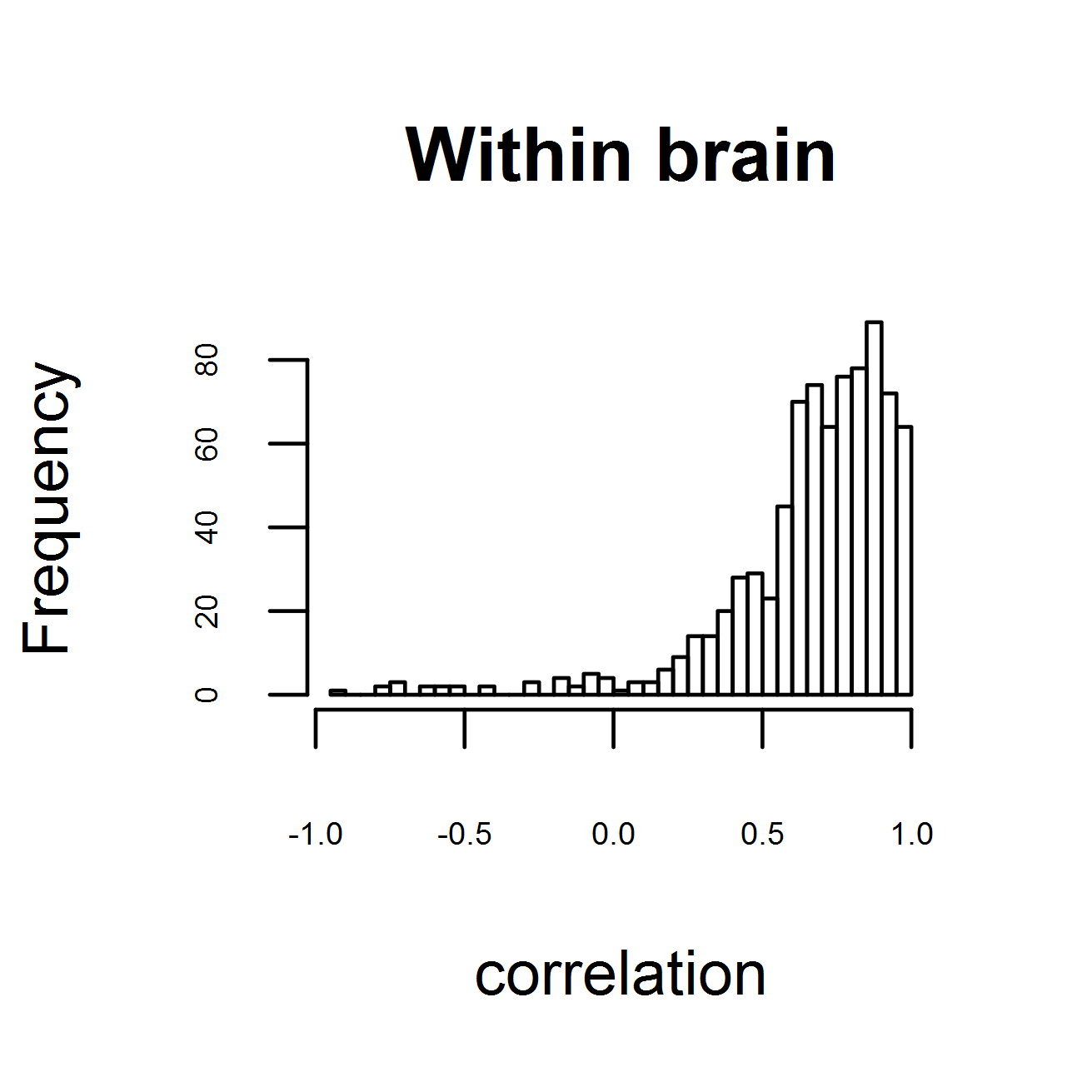

Supplement: Supplementary file 6 — Figure S5 (TIFF 5035 KB) [file 10519_2018_9914_MOESM6_ESM.tiff]

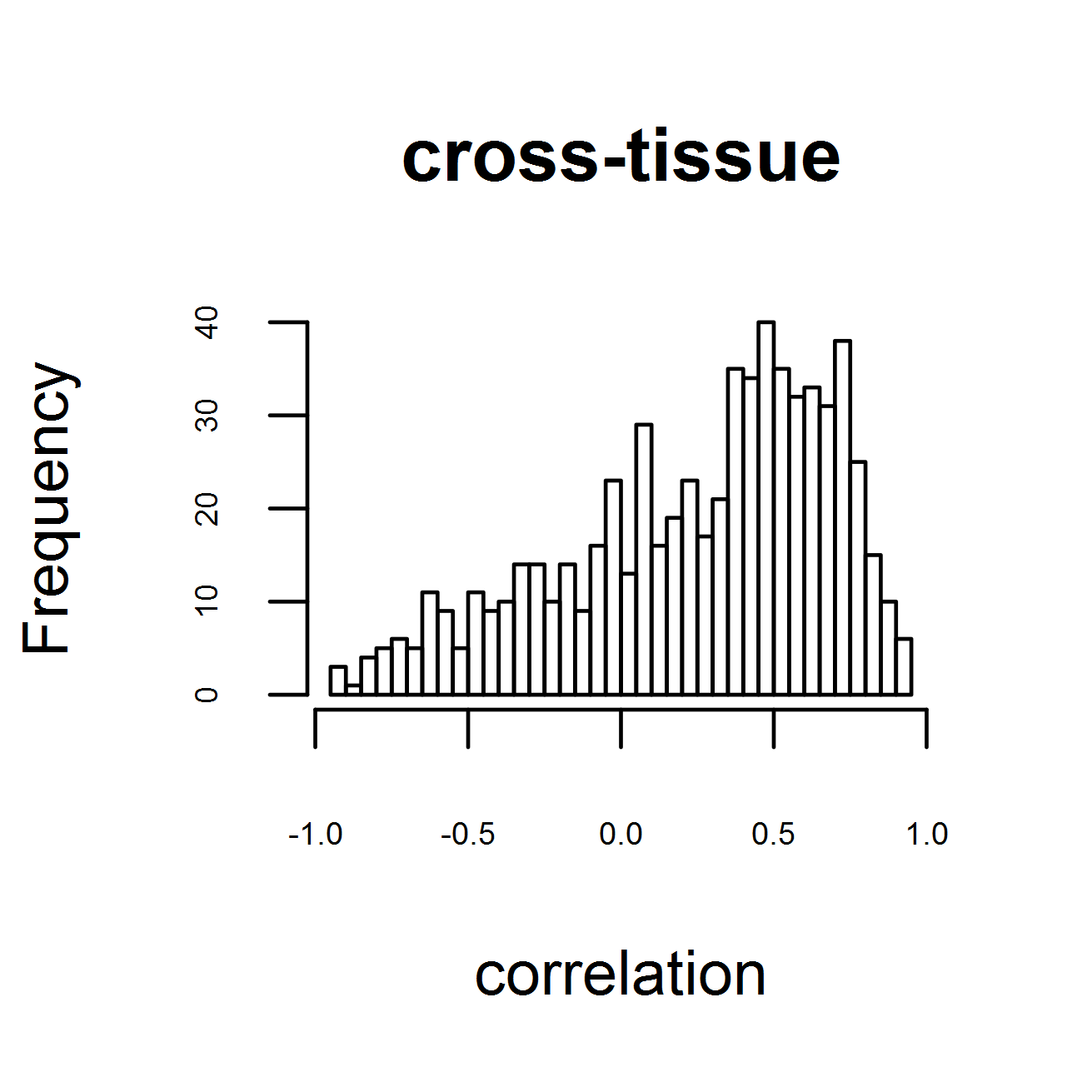

Supplement: Supplementary file 7 — Figure S6 (TIFF 5035 KB) [file 10519_2018_9914_MOESM7_ESM.tiff]
